# Spin Dynamics in Open Quantum Systems: A DLvN-TDDFT Approach

**DOI:** 10.1021/acs.jctc.5c01887

**Published:** 2026-05-26

**Authors:** Kashinath T. Chavan, Oded Hod, Juan E. Peralta

**Affiliations:** † Department of Physics, 5649 Central Michigan University, Mount Pleasant, Michigan 48859, United States; ‡ Department of Physical Chemistry, School of Chemistry, The Raymond and Beverly Sackler Faculty of Exact Sciences, and The Sackler Center for Computational Molecular and Materials Science, 26745 Tel Aviv University, Tel Aviv, 6997801 Israel

## Abstract

We present a spin-uncompensated driven Liouville–von Neumann methodology within the time-dependent density functional theory (DLvN-TDDFT) framework to model collinear electron spin transport in open quantum systems. After introducing and validating the approach, through benchmark simulations of spin-polarized transport in simple molecular junctions, we apply it to a magnetic zigzag graphene nanoribbon junction model under external electric fields. The simulations reveal rich spin-resolved current dynamics, highlighting the DLvN-TDDFT framework as a promising tool for exploring dynamical spintronic phenomena in low-dimensional open quantum systems.

## Graphical abstract

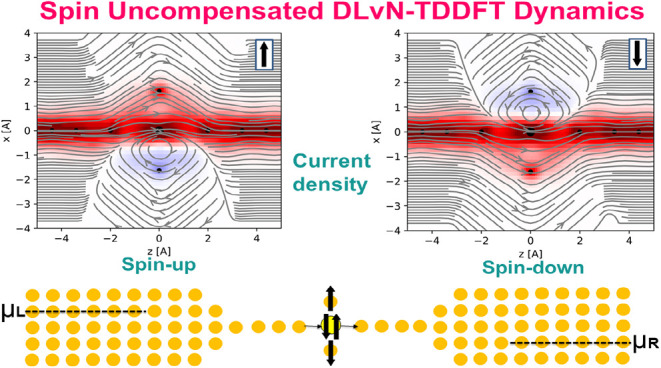

## Introduction

Since the pioneering work of Aviram and Ratner, proposing molecules as electronic rectifiers,^1^ the field of molecular electronics has considerably evolved, offering vast opportunities for electronic component miniaturization, device uniformity, and unique functionalities. Demonstrations of such functionalities include molecular interferometry,^2–7^ and the chirality-induced spin-selectivity (CISS) effect^8–13^ that may be harnessed for advanced technologies.^14–20^ Both examples allow for manipulating the electronic spin degree of freedom, where spin filtering and splitting was demonstrated for multicontact molecular interferometers,^6^ and the CISS effect shows pronounced spin polarization of electrons traversing chiral media, even for weakly spin–orbit coupled channels.^9,12,13^ In this respect, achieving controllable spin transport in molecular devices, will constitute a major milestone toward the realization of nanoscale spintronic devices, allowing to exploit the intrinsic electronic magnetic moment as an additional degree of freedom for information manipulation. Prominent examples of this concept are constantly explored, mainly, giant magneto resistance, tunnel magneto resistance, spin orbit torque, and spin transfer torque.^21–28^ Experiments provide highly valuable information regarding the behavior of single-molecule junction transport.^17,29–32^ Nonetheless, they are limited in their ability to fully decipher the underlying physical mechanisms that are paramount for discovering new dynamical phenomena and for designing and controlling novel devices. Accurate electron dynamics simulations, based on first-principles electronic structure descriptions, provide a central platform for achieving such a level of understanding.

The driven Liouville-von Neumann (DLvN) methodology, developed over the past decade, is a conceptually simple and computationally efficient scheme for simulating electron dynamics in driven open quantum systems.^33–42^ It is particularly useful for the study of transient dynamics in molecular junctions or their response to external time-dependent controls. Following proof-of-concept demonstrations of the approach for simplistic tight-binding and extended Hückel electronic structure descriptions,^39–41,43^ the method was recently extended toward the realm of time-dependent density functional theory (TDDFT).^42,44–47^ This enabled the study of dynamical electronic transport through realistic molecular junction models from first-principles. These implementations, however, could only treat spin-compensated systems, excluding the description of magnetization dynamics and spin transport. To address this limitation, in the present paper we introduce the formalism, implementation, and practical utilization of a collinear spin-uncompensated DLvN-TDDFT simulation approach.

## Methodology

### Collinear Spin-Uncompensated Driven Liouville-von Neumann Formalism

Collinear spin-uncompensated DLvN dynamics requires to solve the two coupled DLvN equations of motion for the spin-resolved one-electron reduced density matrices. In the realm of TDDFT, the DLvN scheme requires frequent back-and-forth transformations between the real-space (site-) and energy (state-) representations of the different sections of the system.^42^ This results from the fact that the construction of the Kohn–Sham (KS) Hamiltonian from the instantaneous density matrix^48,49^ (within the adiabatic approximation) is performed in the site-representation, whereas the open boundary conditions are applied in the state-representation. When the KS molecular orbitals are represented as a linear combination of fixed atom-centered non-orthogonal basis functions, an additional block diagonalization step is required.^40^ A detailed description of the TDDFT-DLvN propagation scheme involving these transformations for spin-compensated systems is provided in ref 42. and Section 1 in the Supporting Information (SI). Here, for completeness, we briefly review the methodology while emphasizing the main modifications required to simulate collinear spin-uncompensated dynamics. The TDDFT-DLvN propagation algorithm involves four major steps, as described below.

#### I Model Definition and Spatial Partitioning

The junction model system is formally divided into three partitions: left lead (marked as L), extended molecule (EM), and right lead (R), as shown in Figure 1. The EM consists of the active molecule, augmented by sufficiently large lead segments that buffer it from the lead sections, where the non-equilibrium boundary conditions are applied. With this partitioning, the spin (σ = ↑, ↓) resolved KS Hamiltonian (***H***_KS_^σ^) and the fixed (for clamped ions) spin independent overlap matrix (***S***) in the non-orthogonal real-space atomic-centered basis, are written as

1 $$H_{\mathrm{KS}}^{\sigma }=\left(\begin{array}{ccc} H_{L}^{\sigma } & V_{L,\mathrm{EM}}^{\sigma } & 0\\ V_{\mathrm{EM},L}^{\sigma } & H_{\mathrm{EM}}^{\sigma } & V_{\mathrm{EM},R}^{\sigma }\\ 0 & V_{R,\mathrm{EM}}^{\sigma } & H_{R}^{\sigma } \end{array}\right)$$

and

2 $$S=\left(\begin{array}{ccc} S_{L} & S_{L,\mathrm{EM}} & 0\\ S_{\mathrm{EM},L} & S_{\mathrm{EM}} & S_{\mathrm{EM},R}\\ 0 & S_{R,\mathrm{EM}} & S_{R} \end{array}\right)$$

respectively. For simplicity we assume that the left and right leads are spatially well-separated such that their real-space coupling (***V***_L,R_^σ^, ***V***_R,L_^σ^) and overlap (***S***_L,R_, ***S***_R,L_) blocks can be neglected, prohibiting direct lead-to-lead tunneling currents.

**Figure 1:**
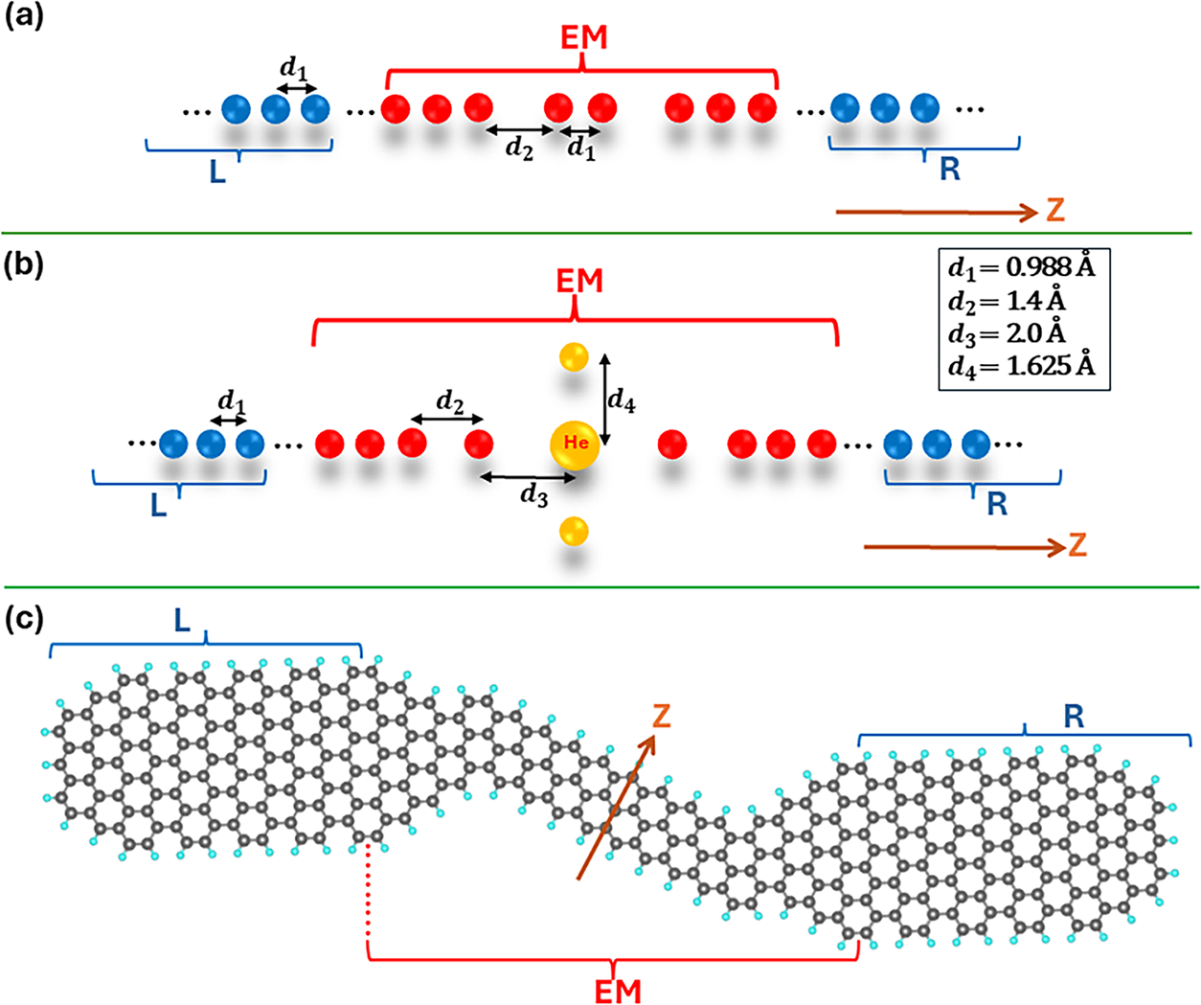
Spatial partitioning of molecular junctions consisting of (a) an H–H molecule bridging two-hydrogen chain lead models, each consisting of 180 hydrogen atoms; (b) a H–He–H linear molecular model bridging two-hydrogen chain lead models, each consisting of 300 hydrogen atoms; and (c) a hydrogen-passivated zigzag graphene nanoribbon segment bridging two armchair graphene nanoribbon lead models. The left lead (L), extended molecule (EM), and right lead (R) partitions for all junctions considered are marked in the figure. The brown arrows mark the Cartesian Z direction.

#### II EM Block Orthogonalization

To ensure that the boundary conditions applied to the lead sections do not directly affect the dynamics in the extended molecule, the atomic-centered basis functions of the EM section are rotated to become orthogonal to those of the leads.^40^ This procedure is performed using a non-unitary transformation matrix (***U***_b_), defined as follows^50^

3 $$U_{b}=\left(\begin{array}{ccc} I_{L} & -S_{L}^{-1}S_{L,\mathrm{EM}} & 0\\ 0 & I_{\mathrm{EM}} & 0\\ 0 & -S_{R}^{-1}S_{R,\mathrm{EM}} & I_{R} \end{array}\right)$$

Here, the ***I***’s and **0**’s are unit and zero matrices of proper dimensions, respectively. The transformation (***U***_b_), performed as follows

4 $$\mathrm{S̃}\equiv U_{b}^{†}{\mathrm{SU}}_{b}=\left(\begin{array}{ccc} S_{L} & 0 & 0\\ 0 & S_{\mathrm{EM}}-S_{\mathrm{EM},L}S_{L}^{-1}S_{L,\mathrm{EM}}-S_{\mathrm{EM},R}S_{R}^{-1}S_{R,\mathrm{EM}} & 0\\ 0 & 0 & S_{R} \end{array}\right)$$

block-diagonalizes the overlap matrix, while retaining the block structure of the KS Hamiltonian matrix

5 $${\mathrm{H̃}}_{\mathrm{KS}}^{\sigma }\equiv U_{b}^{†}H^{\sigma }U_{b}=\left(\begin{array}{ccc} H_{L}^{\sigma } & {Ṽ}_{L,\mathrm{EM}}^{\sigma } & 0\\ {Ṽ}_{\mathrm{EM},L}^{\sigma } & {\mathrm{H̃}}_{\mathrm{EM}}^{\sigma } & {Ṽ}_{\mathrm{EM},R}^{\sigma }\\ 0 & {Ṽ}_{R,\mathrm{EM}}^{\sigma } & H_{R}^{\sigma } \end{array}\right)$$

where, the transformed blocks are

6 $${Ṽ}_{L/R,\mathrm{EM}}^{\sigma }=V_{L/R,\mathrm{EM}}^{\sigma }-H_{L/R}^{\sigma }S_{L/R}^{-1}S_{L/R,\mathrm{EM}}$$

7 $${Ṽ}_{\mathrm{EM},L/R}^{\sigma }=V_{\mathrm{EM},L/R}^{\sigma }-S_{\mathrm{EM},L/R}S_{L/R}^{-1}H_{L/R}^{\sigma }$$

and

8 $${\mathrm{H̃}}_{\mathrm{EM}}^{\sigma }=H_{\mathrm{EM}}^{\sigma }-S_{\mathrm{EM},L}S_{L}^{-1}V_{L,\mathrm{EM}}^{\sigma }-V_{\mathrm{EM},L}^{\sigma }S_{L}^{-1}S_{L,\mathrm{EM}}-S_{\mathrm{EM},R}S_{R}^{-1}V_{R,\mathrm{EM}}^{\sigma }-V_{\mathrm{EM},R}^{\sigma }S_{R}^{-1}S_{R,\mathrm{EM}}+S_{\mathrm{EM},L}S_{L}^{-1}H_{L}^{\sigma }S_{L}^{-1}S_{L,\mathrm{EM}}+S_{\mathrm{EM},R}S_{R}^{-1}H_{R}^{\sigma }S_{R}^{-1}S_{R,\mathrm{EM}}$$

#### III Site-to-State Transformation

Within the DLvN approach, the open boundary conditions are applied in the state representation. For that, the block-orthogonal matrices in the site representation are transformed to the spectral (state) representation using the following unitary transformation matrix

9 $$U^{\sigma }\equiv \left(\begin{array}{ccc} U_{L}^{\sigma } & 0 & 0\\ 0 & U_{\mathrm{EM}}^{\sigma } & 0\\ 0 & 0 & U_{R}^{\sigma } \end{array}\right)$$

where the matrices ***U***_*i*_^σ^ represent the unitary transformations that diagonalize the individual L, R, and EM Hamiltonian blocks ***H* ~**_*i*=L,R,EM_^σ^. Using this transformation, the Kohn–Sham and overlap diagonal matrix blocks in the state representation are given by

10 $${\tilde{\mathrm{H̃}}}_{i}^{\sigma }\;={U_{i}^{\sigma }}^{†}{\mathrm{H̃}}_{i}^{\sigma }U_{i}^{\sigma }$$

and

11 $${\tilde{\mathrm{S̃}}}_{i}={U_{i}^{\sigma }}^{†}{\mathrm{S̃}}_{i}U_{i}^{\sigma }=I_{i}$$

With this, the full overlap matrix becomes the identity and the full KS matrix is

12 $${\tilde{\mathrm{H̃}}}_{\mathrm{KS}}^{\sigma }\;={U^{\sigma }}^{†}{\mathrm{H̃}}_{\mathrm{KS}}^{\sigma }U^{\sigma }=\left(\begin{array}{ccc} {\tilde{\mathrm{H̃}}}_{L}^{\sigma }\; & {\tilde{Ṽ}}_{\mathrm{L,EM}}^{\sigma }\; & 0\\ {\tilde{Ṽ}}_{\mathrm{EM,L}}^{\sigma }\; & {\tilde{\mathrm{H̃}}}_{\mathrm{EM}}^{\sigma }\; & {\tilde{Ṽ}}_{\mathrm{EM,R}}^{\sigma }\;\\ 0 & {\tilde{Ṽ}}_{\mathrm{R,EM}}^{\sigma }\; & {\tilde{\mathrm{H̃}}}_{R}^{\sigma }\; \end{array}\right)$$

and the transformed blocks are, ${\tilde{\mathrm{H̃}}}_{i}^{\sigma }\;={U_{i}^{\sigma }}^{†}{\mathrm{H̃}}_{i}^{\sigma }U_{i}^{\sigma }$ and ${\tilde{Ṽ}}_{i,j}^{\sigma }\;={U_{i}^{\sigma }}^{†}{Ṽ}_{i,j}^{\sigma }U_{j}^{\sigma }$ .

We note that since the transformation matrix ***U*** ^σ^ depends on the KS Hamiltonian and thus on the density matrix, it implicitly depends on time.

#### IV Open Boundary Conditions

The final step involves imposing the boundary conditions using the DLvN equation of motion (see Section 1 in the SI)^36–41,43,51^

13 $${\tilde{\tilde{Ṗ}}}^{\sigma }=-i\left[{\tilde{\mathrm{H̃}}}_{\mathrm{KS}}^{\sigma }\;,{\tilde{\mathrm{P̃}}}^{\sigma }\right]-\Gamma^{\sigma }\left(\begin{array}{ccc} {\tilde{\mathrm{P̃}}}_{L}^{\sigma }-{\tilde{\mathrm{P̃}}}_{L}^{\sigma^{0}} & \frac{1}{2}{\tilde{\mathrm{P̃}}}_{L,\mathrm{EM}}^{\sigma } & {\tilde{\mathrm{P̃}}}_{L,R}^{\sigma }\\ \frac{1}{2}{\tilde{\mathrm{P̃}}}_{\mathrm{EM},L}^{\sigma } & 0 & \frac{1}{2}{\tilde{\mathrm{P̃}}}_{\mathrm{EM},R}^{\sigma }\\ {\tilde{\mathrm{P̃}}}_{R,L}^{\sigma } & \frac{1}{2}{\tilde{\mathrm{P̃}}}_{R,\mathrm{EM}}^{\sigma } & {\tilde{\mathrm{P̃}}}_{R}^{\sigma }-{\tilde{\mathrm{P̃}}}_{R}^{\sigma^{0}} \end{array}\right)$$

Here, ${\tilde{\mathrm{P̃}}}^{\sigma }=(U^{\sigma }{)}^{-1}U_{b}^{-1}P^{\sigma }(U_{b}^{†}{)}^{-1}({U^{\sigma }}^{†}{)}^{-1}$ is the density matrix in the state representation. Note that the left-hand-side of eq 13 accounts for the transformed time derivative of the density matrix in the site representation (***P*** ^σ^) and not for the time derivative of the density matrix in the state representation. This results from the time dependency of the site-to-state transformation matrix and the fact that one does not have an explicit equation of motion for it.^42^ The first term on the right-hand-side of eq 13 provides the unitary dynamics according to the Liouville-von Neumann equation of motion, whereas the open boundary conditions are applied via the second term that drives the lead sections toward the equilibrium state of the corresponding implicit reservoirs at a driving rate Γ^σ^ that represents the thermal relaxation rate in the leads. This term mimics simultaneous source and sink terms that inject bath-equilibrated electrons into the system while depleting outgoing electrons that move toward the leads, taking appropriate care of their coherences.^40^ While the latter can be determined from first-principles,^41^ it is often obtained according to the typical level spacing of the finite lead model (ℏΓ^σ^ ∼ Δ*ϵ*_L/*R*_^σ^) within the Fermi transport window, set by the bias voltage, which should be done with care.^52–54^ Using this Γ^σ^, the density of states (DOS) of the finite lead model are broadened to mimic that of a semi-infinite lead (see Section 2 in the SI). We note that using a uniform driving rate in the state-representation translates to position-dependent Γ, in analogy with the method of complex absorbing potentials.^55,56^

The two diagonal blocks $\left({\tilde{\mathrm{P̃}}}_{L}^{\sigma }-{\tilde{\mathrm{P̃}}}_{L}^{\sigma^{0}}\right)$ and $\left({\tilde{\mathrm{P̃}}}_{R}^{\sigma }-{\tilde{\mathrm{P̃}}}_{R}^{\sigma^{0}}\right)$ drive the lead density matrix blocks $\left({\tilde{\mathrm{P̃}}}_{L/R}^{\sigma }\right)$ toward their diagonal target density matrices

14 $$[{\tilde{\mathrm{P̃}}}_{L/R}^{\sigma^{0}}{]}_{n,m}=\delta_{n,m}{\left[1+e^{\left(\epsilon_{n}^{L/R}-\mu_{L/R}^{\sigma }\right)/\left(k_{B}T_{L/R}^{\sigma }\right)}\right]}^{-1}$$

holding on their diagonal the Fermi–Dirac occupation distribution of the implicit baths, sampled at the lead model state energies. In eq 14, *k*_*B*_ is Boltzmann’s constant, *T*_L/R_^σ^ are the temperatures of the implicit left and right reservoirs, and

15 $$\mu_{L/R}^{\sigma }=\epsilon_{F_{L/R}}^{\sigma }\pm \frac{1}{2}\left|e\right|V^{\sigma }$$

are the corresponding chemical potentials, where ϵ_F_L/R__^σ^ are the lead Fermi energies, *V* ^σ^ is the applied bias voltage, and *e* is the electron charge. Note that in the spin-uncompensated case, one has the flexibility to apply spin-dependent bias voltage, lead temperatures, and driving rate in DLvN-TDDFT simulations.

Since the propagation takes place in the site-representation, after applying the open boundary conditions, the driving term (second term on the RHS of eq 13, referred to here as ***D***, for simplicity) needs to be back-transformed from the state- to the site-representation at every time-step. The back-transformation is performed according to

16 $$D=U_{b}U^{\sigma }{\tilde{\mathrm{D̃}}}^{\sigma }\;{U^{\sigma }}^{†}U_{b}^{†}$$

### Implementation

The DLvN implementation within the TDDFT framework can be broken down into the following steps:

1. A ground-state DFT calculation of the entire finite model system (L + EM +R) is performed from which the KS Hamiltonian, overlap, and density matrices are extracted in the site representation, ordered according to their spatial partitioning.
2. Block orthogonalizations and site-to-state transformations of the KS Hamiltonian (eqs 5 and 12, respectively) and density matrices (eqs S23 and S28, respectively, in the SI) are performed.
3. The target density matrices are constructed (eq 14) and the driving term is built in the state representation.
4. The driving term in eq 13 in the state-representation is back-transformed to the site-representation (eq S39 in the SI) using the transformation of eq 16.
5. The single-particle density matrix is propagated (eq S39 in the SI) using an implicit Euler adaptive time step method and an updated KS Hamiltonian is constructed from it.^42^
6. Return to step 2.

Finally, to evaluate the spin-resolved current flowing through the extended molecule section one should transform to the block-orthogonal representation^42^ (see Section 3 in the SI) and use the relation (in atomic units)

17 Jσ(t)=Im{trEM[P̃EM,Lσ(t)ṼL,EMσ(t)−P̃EM,Rσ(t)ṼR,EMσ(t)]}

In the present study, this spin-uncompensated DLvN propagation scheme has been implemented using a Python^57^ driver code that utilizes a local version of the Gaussian suite of programs^58^ to construct the KS matrix from the instantaneous density matrix at every time step. The computational cost of the DLvN approach can be traced to two main sources: (i) construction of the Kohn–Sham Hamiltonian, and (ii) linear algebra operations associated with the site-to-state transformation. The corresponding scaling with system dimensions is determined by the specific algorithms used to perform these operations. In its current implementation, the method is expected to scale as *O*(*N* ^3^), although lower scaling may be achievable through various algorithmic improvements, which is the subject of future work.

## Results and Discussion

The spin-uncompensated DLvN methodology was first tested for two simple model junctions involving two hydrogen chain leads bridged by either an axially oriented hydrogen molecule or a perpendicularly oriented linear H–He–H molecule (see Figure 1(a and b)). The former serves to compare against our previous spin-compensated calculations, whereas the latter, which is often used to assess the reliability of different methods for evaluating magnetic properties, serves as a simplistic spin-uncompensated bridge model.^59,60^

With an even number of H atoms, the ground state of the all-hydrogen chain is spin-compensated. Figure 2 shows, as a consistency check, the spin uncompensated DLvN dynamics where the same bias is applied to both spin-channels (dashed green and full purple lines), which matches the current of the spin-compensated DLvN dynamics using the same propagation parameters (dotted black line), as expected.^42^

**Figure 2:**
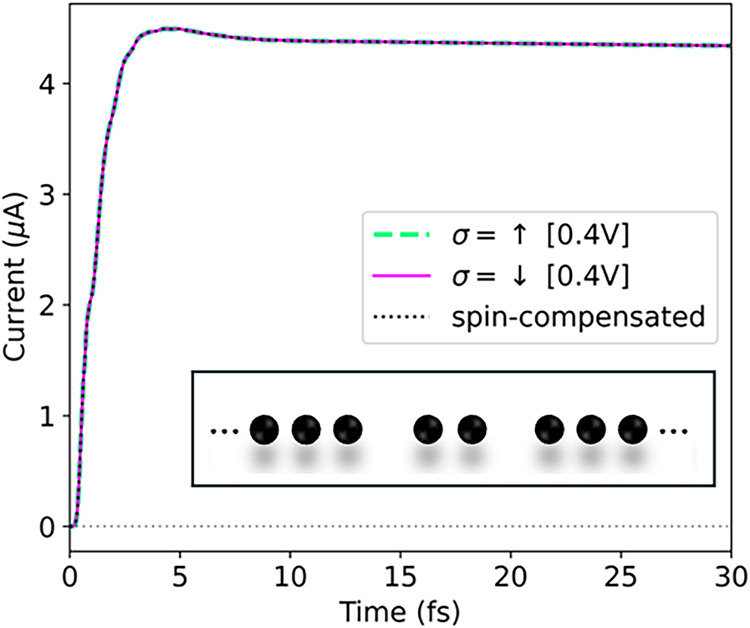
Time-dependent current through an H–H molecule bridging two hydrogen chain leads. The finite model system is composed a total of 380 atoms, with two 180 atom leads, and an extended molecule region of 20 atoms, out of which the central two serve as the active H–H molecule. All interatomic separations are *d*_1_ = 0.988 Å, except that of the central molecule that is separated by *d*_2_ = 1.4 Å from the two extended lead sections (see Figure 1(a)). The PBE density functional approximation^61^ is used in combination with a mixed basis set (STO-3G for the leads and 6-31G** ^62,63^ for the extended molecule) to evaluate the electronic structure of the system, yielding lead midgap energies of ϵ_*F*_ = −4.1012 *eV*. A bias voltage of 0.4 *V* is applied and the electronic temperatures are set to *T*_L/R_^↑↓^ = 315.7 *K* with a driving rate of ℏΓ^↑↓^ = 0.61 eV.^42^ The dashed green (↑-spin) and full purple (↓-spin) lines are the current traces obtained via the spin-uncompensated DLvN simulations, whereas the dotted black line corresponds to spin-compensated DLvN dynamics. The inset depicts the central part of the molecular junction.

Replacing the H–H molecule with a H–He–H bridge yields spin-uncompensated dynamics even for a symmetric spin-bias (where the same bias is applied to both spin channels), with access to the (slightly lower in energy) broken symmetry antiferromagnetic bridge spin configuration and to its ferromagnetic counterpart (see insets of Figure 3(a and b), respectively). Starting from the antiferromagnetic state, applying a symmetric spin-bias yields identical current traces for the two spin-channels (Figure 3(a)). Notably, despite having the same numerical value, the ↑- and ↓-spin-resolved steady-state currents flow through different spatial routes. Apart from the central spin unpolarized path (through the He atom), the ↑-spin electrons detour also through the bottom H bridge atom bearing ↑-spin polarization (Figure 3(c)), whereas the ↓-spin electrons detour through the ↓-spin polarized upper H bridge atom (Figure 3(e)). Starting the dynamics from the ferromagnetic configuration results in an increase of the ↑-spin current and a decrease in the ↓-spin current (Figure 3(b)). This can be rationalized by the fact that the ↑-spin electrons can flow through the central He path and through the two ↑-spin polarized side H channels (see Figure 3(d)), whereas the ↓-spin electrons flow only via the central path (see Figure 3(f)). Namely, in its ferromagnetic spin configuration, the H–He–H molecular junction bears an imbalance in the number of ↑- and ↓-spin transport channels, resulting in net spin current.

**Figure 3:**
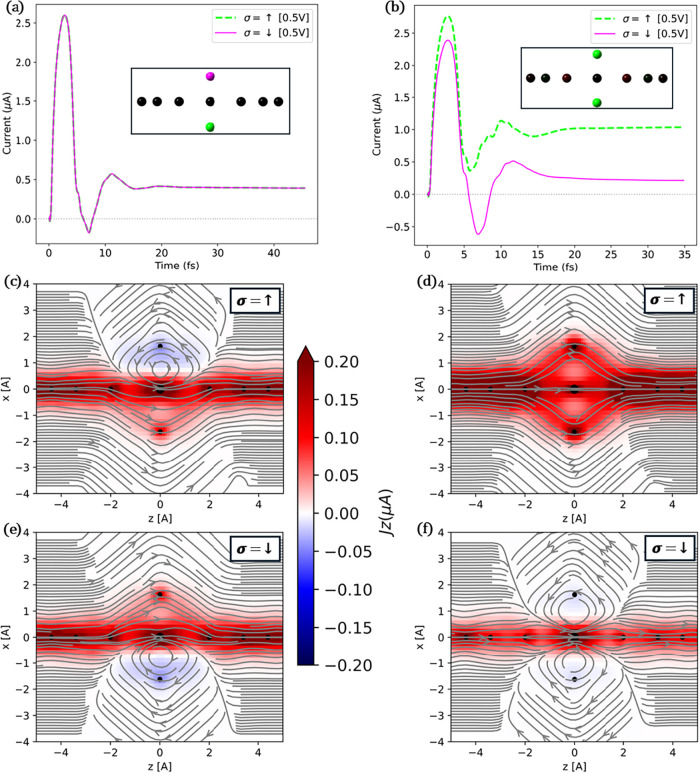
Spin-resolved current traces obtained from the DLvN-TDDFT dynamics of a molecular junction model consisting of two hydrogen chain leads bridged by a perpendicular H–He–H magnetic molecule starting from either the (a) antiferromagnetic or (b) ferromagnetic configuration (see insets for color coded Mülliken spin population maps of the molecular bridge, where green, purple, and black mark Mülliken spin population of 0.977, −0.997 and 0, respectively). (c) and (d) show the spatially resolved heatmaps of the ↑ electrons steady-state current densities in the antiferromagnetic (c) and ferromagnetic (d) configurations. (e) and (f) same as (c) and (d) but for the ↓ electrons. The system consists of a total of 703 atoms with two 300 hydrogen atom leads, two 50 hydrogen atoms lead sections in the extended molecule and the central H–He–H molecular bridge. The H–H bond length is taken to be *d*_1_ = 0.988 Å throughout the leads apart from the last atoms near the molecular bridge, where a somewhat larger distance of *d*_2_ = 1.4 Å is taken to keep the leads mostly spin-compensated. The distance between these H edge atoms and the central He atom of the molecular bridge is taken to be *d*_3_ = 2.0 Å. The He–H distances in the vertical bridge are *d*_4_ = 1.625 Å (see Figure 1(b) and coordinates in the SI). The PBE density functional approximation^61^ is used in combination with 6-31G** ^62,63^ to evaluate the electronic structure of the system, yielding lead midgap energies of ϵ_*F*_ = – 4.1778 *eV*. A bias voltages of 0.5 V was applied with reservoir electronic temperatures of *T*_L/*R*_^↑↓^ = 315.7 *K* and a driving rate of ℏΓ^↑↓^ = 0.26 *eV* (see Section 2 of the SI for details). Sensitivity tests of the current traces toward various calculation parameters are shown in Sections 5–8 of the SI. The absence of sizable ghost current^64,65^ contamination is discussed in Section 9 of the SI.

Typically, the applied bias voltage is considered to be the same for both spin channels. Nonetheless, one can envision situations, involving, e.g., spin polarizers or filters, where different effective biases are applied to the ↑- and ↓-spin channels. In the DLvN approach, this can be modeled by introducing a spin-dependent bias voltage via eq 15. To demonstrate this, we have applied spin-dependent voltages to the ferromagnetic H–He–H junction, such that either only the ↑-spin channel or its ↓-spin counterpart are biased. Figure 4 shows the corresponding spin-dependent current traces for the (a) ↑-biased, and (b) ↓-biased junctions. In both cases, following initial *downstream* transient dynamics, the biased channel reaches a finite steady-state current, as expected. Notably, the nonbiased channel demonstrates a significant *upstream* transient current, eventually vanishing at steady-state. This intriguing phenomenon requires more investigation to unveil its origin and robustness, which we intend to explore in future studies. As may be expected, when only the ↑-spin channel is biased, the steady-state ↓-spin current is nearly zero (see Figure 4(e)) and the ↑-spin electrons flow through both ↑-spin polarized side bridge H atoms and the central spin unpolarized He path (see Figure 4(c)). Biasing only the ↓-spin channel, the ↑-spin current is nearly zero (see Figure 4(d)) and the ↓-spin electrons flow only through the central spin unpolarized path (see Figure 4(f)) resulting in a lower steady-state total current. Similar results are presented in Section 4 of the SI for the antiferromagnetic configuration.

**Figure 4:**
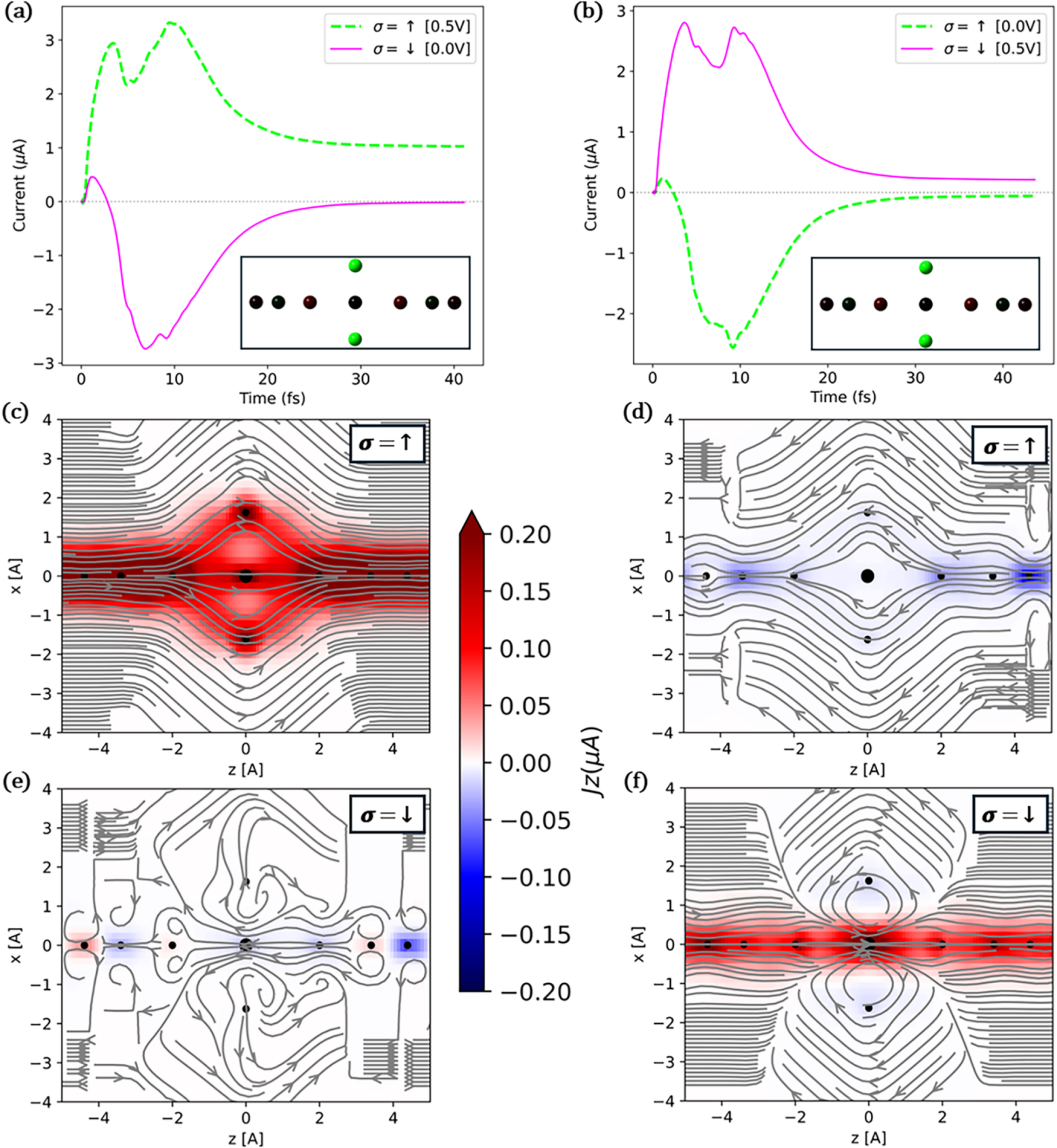
Spin-resolved current traces obtained from the DLvN-TDDFT dynamics of the H–He–H molecular junction model (see Figure 1(b)) starting from the ferromagnetic configuration (see insets of panels (a) and (b) for color coded Mülliken spin populations of the molecular bridge, where green and black mark Mülliken spin populations of 0.977 and 0, respectively). A bias voltage of 0.5 V is applied to (a) only the ↑-spin channel, and (b) only the (↓)-spin channel. (c) and (e) show the spatially resolved heatmaps corresponding to the ↑ and ↓ steady-state current densities for the case where only the ↑-spin channel is biased. (d) and (f) are the same as (c) and (e), respectively, but for the case where only the ↓-spin channel is biased. The junction model parameters are given in the caption of Figure 3.

Following the benchmark calculations on simple model systems, we now turn to demonstrate the developed methodology for a realistic model junction consisting of armchair graphene nanoribbon (GNR) leads, bridged by a zigzag GNR segment (see Figure 1(c)). This forms a spin-polarized junction (see Figure 5(a)), whose magnetic properties can be tuned using an external transversal in-plane electric field.^66,67^

**Figure 5:**
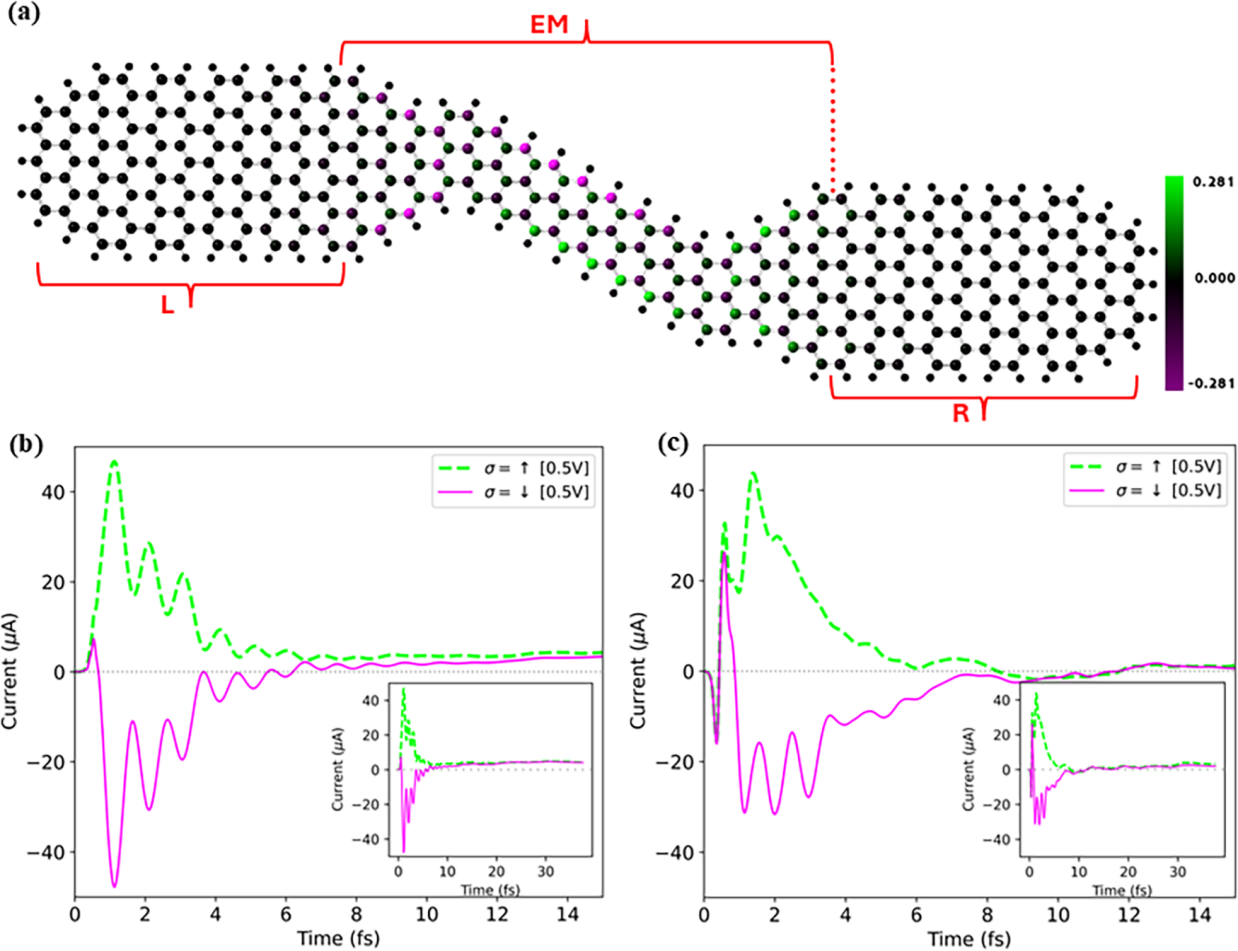
Spin resolved current traces in a GNR-based model junction. (a) Schematic representation of the junction. The system contains of a total of 434 atoms with two 140-atom armchair GNR lead models (marked as L and R) and a 154-atom extended molecule section (marked as EM), consisting of two lead extensions and the central zigzag GNR. All peripheral carbon atoms are hydrogen passivated. Atoms are colored according their initial ground-state Mülliken populations (see color bar). (b) Spin resolved current traces obtained under an applied bias voltage of 0.5 V. (c) Dynamical response of the system to the gradual application of an in-plane electric field of 0.6170 V/Å perpendicular to the zigzag GNR axis that is ramped up using a hyperbolic-tangent function, reaching its full value after ∼ 1.38 fs (see Section 10 of the SI for details). The PBE density functional approximation^61^ is used in conjunction with the STO-3G and the 6-31G** ^62,63^ atom-centered basis sets for the leads and the EM sections, respectively, yielding lead midgap energies of ϵ_*F*_ = −3.7404 *eV*. The electronic temperatures used are *T*_L/R_^↑↓^ = 315.7 *K*, and the driving rate is chosen to be ℏΓ^↑↓^ = 1.00 *eV*, which corresponds to lead states Lorentzian broadening width that yields a density of lead states mimicking that of the corresponding infinite nanoribbon (see Section 2 and Figure S3 of the SI). The insets in panels (b) and (c) present extended spin resolved current traces, whereas the main panels present a zoom-in on the transient stage.

Notably, even when biasing both spin channels, a significant transient *upstream* current is demonstrated for the ↓-spin transport channel (see Figure 5(b)), which nearly mirrors the *downstream* dynamics of the corresponding ↑-spin counterpart. This can be qualitatively rationalized by the fact that when the junction is initially prepared in an antiferromagnetic configuration of the central zigzag GNR section (see Figure 5(a)), spin polarization extends toward the narrowing lead segments in the EM section. Here, the left junction entrance is ↓-spin-polarized, whereas the right exit is ↑-spin-polarized. This creates a Pauli conduction barrier for ↓-spin electrons to flow from left to right, once the bias is switched on. When the ↑-spin electrons reach the exit of the junction, they encounter a similar Pauli conductance barrier resulting in reflections, with a corresponding response of the ↓-spin counterparts. This is manifested as current oscillations in the transient current trace. Eventually, the electrostatic response of the system leads to a steady-state, where the currents of both spin-channels converge to a consistent *downstream* current.

Switching on an electric field perpendicular to the main axis of the central zigzag GNR section (z direction, see Figure 1(c) and SI Figure S13) breaks the transient current mirror symmetry of the two spin-channels (see Figure 5(c)) and splits the steady-state currents (see Figure 5(c), inset). Consistent with the reduction­(increase) of the ↑(↓)-spin gap due to the external electric field (see Figure S14 of the SI), the ↑-current is higher both for the transient stage and near steady-state. This demonstrates that collinear spin-uncompensated DLvN simulations can be employed to study the control over complex phenomena, such as dynamical spin filtering.

## Summary and Conclusions

We present a collinear spin-uncompensated Driven Liouville–von Neumann approach within the time-dependent density functional theory framework to simulate spin transport in open quantum systems. This methodology enables the real-time simulation of space-resolved spin- and electron currents, offering a powerful tool to investigate magnetic molecular junctions under dynamical conditions. To validate the approach, we first apply it to simple model systems, namely H–H and H–He–H molecular junction bridges, demonstrating its capability to capture spin-dependent transport phenomena across various spin configurations and biasing schemes. We then extend the analysis to a more realistic graphene nanoribbon-based junction, where the application of an external electric field induces distinct spin-resolved current dynamics, highlighting the ability of the method to simulate the influence of external perturbations on nanoscale spintronic devices. Taken together, these results establish the DLvN-TDDFT framework as a robust and versatile platform for exploring spin dynamics in open magnetic quantum architectures, paving the way for future studies in molecular spintronics and quantum transport engineering, via external time-dependent controls.

## Supplementary Material



## Data Availability

Link to GitHub repository: https://github.com/ktchavan99/TDDFT_UDLvN.git

## Notes

Driven Liouville von Neumann equation of motion in the realm of spin-uncompensated time-dependent density functional theory; driving rate sensitivity test; total current calculation; current dynamics for the H–He–H junction with antiferromagnetic spin configuration; sensitivity of the transient dynamics of the H–He–H bridged molecular junction toward the driving rate; sensitivity of the transient dynamics of the H–He–H bridged molecular junction toward the driving term switch-on procedure; effect of the explicit lead model dimensions on the transient current traces of the H–He–H bridged molecular junction; sensitivity of the transient dynamics of the H–He–H bridged molecular junction toward the convergence tolerance used in the implicit Euler propagator; estimation of the effect of ghost currents; determination of the electric field strength for the zigzag GNR calculation; cartesian coordinates of the H–H-based molecular junction model; cartesian coordinates of the H–He–H-based molecular junction model; cartesian coordinates of the zigzag GNR-based molecular junction model (PDF)
