# Supplementary material for: Spin Dynamics in Open Quantum Systems: A DLvN-TDDFT Approach
Source: J Chem Theory Comput. 2026 May 26;22(11):5363–72. doi: 10.1021/acs.jctc.5c01887 (PMC13255247; doi:10.1021/acs.jctc.5c01887)
Supplement: Supplementary file 1 [file ct5c01887_si_001.pdf]

Spin Dynamics in Open Quantum Systems: A DLvN-TDDFT Approach

Supporting Information

Kashinath T. Chavan,[1*] Oded Hod,[2,**] Juan E. Peralta,[1,***]

[1]*Department of Physics, Central Michigan University, Mount Pleasant, MI, USA 48859*

[2]*Department of Physical Chemistry, School of Chemistry, The Raymond and Beverly Sackler Faculty of Exact Sciences, and The Sackler Center for Computational Molecular and Materials Science, Tel Aviv University, Tel Aviv, Israel 6997801*

[*] ktchavan99@gmail.com

[**]odedhod@tauex.tau.ac.il

[***]peral1j@cmich.edu

Contents

1. The driven Liouville von Neumann equation of motion in the realm of spin-uncompensated time-dependent density functional theory 2
2. Driving rate sensitivity test 11
3. Total current calculation 13
4. Current dynamics for the H-He-H junction with anti-ferromagnetic spin configuration 24
5. Sensitivity of the transient dynamics of the H-He-H bridged molecular junction towards the driving rate. 25
6. Sensitivity of the transient dynamics of the H-He-H bridged molecular junction towards the driving term switch-on procedure. 26
7. Effect of the explicit lead model dimensions on the transient current traces of the H-He-H bridged molecular junction....................27
8. Sensitivity of the transient dynamics of the H-He-H bridged molecular junction towards the convergence tolerance used in the implicit Euler propagator 28
9. Estimation of the effect of ghost currents 29
10. Determination of the electric field strength for the zigzag GNR calculation 31
11. Cartesian coordinates of the H-H-based molecular junction model 32
12. Cartesian coordinates of the H-He-H-based molecular junction model 39
13. Cartesian coordinates of the zigzag GNR-based molecular junction model 52
14. References 60

1. The driven Liouville von Neumann equation of motion in the realm of spin-uncompensated time-dependent density functional theory

In this section, we provide a detailed formulation of the driven Liouville von Neumann (DLvN) equation of motion (EOM) within the framework of collinear spin-uncompensated time-dependent density functional theory in an atom-centered non-orthogonal basis-set representation. We start from the standard time-dependent Kohn-Sham (KS) equation written for the individual KS orbitals $|\phi_{n\sigma}\rangle$ as follows (atomic units are used throughout):

$$|\dot{\phi}_n^\sigma\rangle = -i\mathcal{H}_{KS}^\sigma|\phi_n^\sigma\rangle. \tag{S1}$$

Here, $\mathcal{H}_{KS}^\sigma$ is the KS Hamiltonian operator and $i = \sqrt{-1}$. n and σ refer to state and spin indices, respectively. Next, we span the KS orbitals within a localized basis-set representation $\{|\chi_\mu\rangle\}$:

$$|\phi_n^\sigma\rangle = \sum_\mu c_\mu^{n\sigma}|\chi_\mu\rangle, \tag{S2}$$

where $c_\mu^{n\sigma}$ is the μth expansion coefficient of KS orbital $|\phi_n^\sigma\rangle$. Plugging Eq. (S2) into Eq. (S1) and assuming that the basis orbitals are constant in time we obtain:

$$\sum_\mu \dot{c}_\mu^{n\sigma}|\chi_\mu\rangle = -i\mathcal{H}_{KS}^\sigma \sum_\mu c_\mu^{n\sigma}|\chi_\mu\rangle. \tag{S3}$$

Multiplying Eq. (S3) by $\langle\chi_\nu|$ we obtain:

$$\sum_\mu \dot{c}_\mu^{n\sigma}\langle\chi_\nu|\chi_\mu\rangle = -i\sum_\mu c_\mu^{n\sigma}\langle\chi_\nu|\mathcal{H}_{KS}^\sigma|\chi_\mu\rangle. \tag{S4}$$

Defining the overlap and KS Hamiltonian matrix elements as $S_{\nu\mu} \equiv \langle\chi_\nu|\chi_\mu\rangle$ and $\mathcal{H}_{KS_{\nu\mu}}^\sigma \equiv \langle\chi_\nu|\mathcal{H}_{KS}^\sigma|\chi_\mu\rangle$, respectively, Eq. (S4) becomes:

$$\sum_\mu S_{\nu\mu}\dot{c}_\mu^{n\sigma} = -i\sum_\mu \mathcal{H}_{KS_{\nu\mu}}^\sigma c_\mu^{n\sigma}. \tag{S5}$$

Since this equation is valid for all values of the indices ν and n it can be written in matrix form as:

$$\boldsymbol{S}\dot{\boldsymbol{C}}^\sigma = -i\boldsymbol{\mathcal{H}}_{KS}^\sigma\boldsymbol{C}^\sigma. \tag{S6}$$

Multiplying by the inverse of the overlap matrix, $\boldsymbol{S}^{-1}$, on the left we obtain:

$$\dot{\boldsymbol{C}}^\sigma = -i\boldsymbol{S}^{-1}\boldsymbol{\mathcal{H}}_{KS}^\sigma\boldsymbol{C}^\sigma. \tag{S7}$$

Accordingly, one can write the EOM for the complex transpose coefficients matrix as follows:

$$\dot{\boldsymbol{C}}^{\sigma\dagger} = [-i\boldsymbol{S}^{-1}\boldsymbol{\mathcal{H}}_{KS}^\sigma\boldsymbol{C}^\sigma]^\dagger = i\boldsymbol{C}^{\sigma\dagger}\boldsymbol{\mathcal{H}}_{KS}^{\sigma\,\dagger}(\boldsymbol{S}^{-1})^\dagger = i\boldsymbol{C}^{\sigma\dagger}\boldsymbol{\mathcal{H}}_{KS}^\sigma(\boldsymbol{S}^\dagger)^{-1} = i\boldsymbol{C}^{\sigma\dagger}\boldsymbol{\mathcal{H}}_{KS}^\sigma\boldsymbol{S}^{-1}, \tag{S8}$$

where we used the relation $(\boldsymbol{S}^{-1})^\dagger = (\boldsymbol{S}^\dagger)^{-1}$ $\left(\boldsymbol{I} = \boldsymbol{I}^\dagger = (\boldsymbol{S}\boldsymbol{S}^{-1})^\dagger = (\boldsymbol{S}^{-1})^\dagger\boldsymbol{S}^\dagger \Rightarrow (\boldsymbol{S}^{-1})^\dagger = (\boldsymbol{S}^\dagger)^{-1}\right)$ and the fact that the overlap and Kohn-Sham matrices are Hermitian, such that $\boldsymbol{S}^\dagger = \boldsymbol{S}$ and $\boldsymbol{\mathcal{H}}_{KS}^\sigma = \boldsymbol{\mathcal{H}}_{KS}^{\sigma\,\dagger}$. The latter relation stems from the fact that the density matrix, upon which $\boldsymbol{\mathcal{H}}_{KS}^\sigma$ depends, is Hermitian by construction (see Eq. (S9) below) and so are all the operators within $\boldsymbol{\mathcal{H}}_{KS}^\sigma$ (kinetic energy,

Hartree, exchange, correlation, and the external potential).

We can now define the single-particle density matrix in the localized basis-set representation as:

$$\boldsymbol{\mathcal{P}}^{\sigma} = \boldsymbol{\mathcal{C}}^{\sigma}\boldsymbol{n}^{\sigma}\boldsymbol{\mathcal{C}}^{\sigma\dagger}, \tag{S9}$$

where $\boldsymbol{n}^{\sigma}$ is a diagonal matrix holding the occupation numbers of the different single-particle states on its diagonal. The time evolution of the density matrix is obtained by its time derivative:

$$\dot{\boldsymbol{\mathcal{P}}}^{\sigma} = \dot{\boldsymbol{\mathcal{C}}}^{\sigma}\boldsymbol{n}^{\sigma}\boldsymbol{\mathcal{C}}^{\sigma\dagger} + \boldsymbol{\mathcal{C}}^{\sigma}\boldsymbol{n}^{\sigma}\dot{\boldsymbol{\mathcal{C}}}^{\sigma\dagger} + \boldsymbol{\mathcal{C}}^{\sigma}\dot{\boldsymbol{n}}^{\sigma}\boldsymbol{\mathcal{C}}^{\sigma\dagger}. \tag{S10}$$

Here, the first two terms on the right-hand-side correspond to pure orbital dynamics, whereas the third term represents the dynamics of the orbital occupations. Inserting Eqs. (S7) and (S8) into Eq. (S10) we obtain:

$$\begin{aligned}\dot{\boldsymbol{\mathcal{P}}}^{\sigma} &= -i\boldsymbol{\mathcal{S}}^{-1}\boldsymbol{\mathcal{H}}_{KS}^{\sigma}\boldsymbol{\mathcal{C}}^{\sigma}\boldsymbol{n}^{\sigma}\boldsymbol{\mathcal{C}}^{\sigma\dagger} + i\boldsymbol{\mathcal{C}}^{\sigma}\boldsymbol{n}^{\sigma}\boldsymbol{\mathcal{C}}^{\sigma\dagger}\boldsymbol{\mathcal{H}}_{KS}^{\sigma}\boldsymbol{\mathcal{S}}^{-1} + \boldsymbol{\mathcal{C}}^{\sigma}\dot{\boldsymbol{n}}^{\sigma}\boldsymbol{\mathcal{C}}^{\sigma\dagger}\\ &= -i\boldsymbol{\mathcal{S}}^{-1}\boldsymbol{\mathcal{H}}_{KS}^{\sigma}\boldsymbol{\mathcal{P}}^{\sigma} + i\boldsymbol{\mathcal{P}}^{\sigma}\boldsymbol{\mathcal{H}}_{KS}^{\sigma}\boldsymbol{\mathcal{S}}^{-1} + \boldsymbol{\mathcal{C}}^{\sigma}\dot{\boldsymbol{n}}^{\sigma}\boldsymbol{\mathcal{C}}^{\sigma\dagger}.\end{aligned} \tag{S11}$$

In microcanonical and canonical time-domain time-dependent density functional theory simulations, the overall number of particles in the system is conserved. In these cases, a customary ansatz is to propagate only the occupied subspace thus setting $\dot{\boldsymbol{n}}^{\sigma} = 0$, assuming that the occupied KS orbital populations do not vary with time and that the virtual orbitals remain unpopulated. The entire dynamics is thus overloaded on the occupied molecular orbital manifold via the corresponding expansion coefficients. This resembles choosing the Schrödinger representation (propagating the wave functions) instead of its Heisenberg counterpart (propagating the number operator). For open systems, however, one can no longer assume that $\dot{\boldsymbol{n}}^{\sigma} = 0$ and an explicit equation of motion should be provided to describe its dynamics. Within the DLvN approach the following EOM ansatz governs this dynamics:

$$\begin{aligned}\boldsymbol{\mathcal{C}}^{\sigma}\dot{\boldsymbol{n}}^{\sigma}\boldsymbol{\mathcal{C}}^{\sigma\dagger} &= -i\boldsymbol{\mathcal{S}}^{-1}\boldsymbol{\mathcal{H}}_{AH}^{\sigma}\boldsymbol{\mathcal{P}}^{\sigma} + i\boldsymbol{\mathcal{P}}^{\sigma}{\boldsymbol{\mathcal{H}}_{AH}^{\sigma}}^{\dagger}\boldsymbol{\mathcal{S}}^{-1} = -i\boldsymbol{\mathcal{S}}^{-1}\boldsymbol{\mathcal{H}}_{AH}^{\sigma}\boldsymbol{\mathcal{P}}^{\sigma} - i\boldsymbol{\mathcal{P}}^{\sigma}\boldsymbol{\mathcal{H}}_{AH}^{\sigma}\boldsymbol{\mathcal{S}}^{-1}\\ &= -i[\boldsymbol{\mathcal{S}}^{-1}\boldsymbol{\mathcal{H}}_{AH}^{\sigma}\boldsymbol{\mathcal{P}}^{\sigma} + \boldsymbol{\mathcal{P}}^{\sigma}\boldsymbol{\mathcal{H}}_{AH}^{\sigma}\boldsymbol{\mathcal{S}}^{-1}].\end{aligned} \tag{S12}$$

Here, $\boldsymbol{\mathcal{H}}_{AH}^{\sigma} = -{\boldsymbol{\mathcal{H}}_{AH}^{\sigma}}^{\dagger}$ is an anti-Hermitian matrix that, in principle, can assume the most general form of $\boldsymbol{\mathcal{H}}_{AH}^{\sigma} = {\boldsymbol{\mathcal{H}}_{AH}^{\sigma}}^{re} - i{\boldsymbol{\mathcal{H}}_{AH}^{\sigma}}^{im}$, where ${\boldsymbol{\mathcal{H}}_{AH}^{\sigma}}^{re}$ is a real anti-symmetric matrix such that $\left({\boldsymbol{\mathcal{H}}_{AH}^{\sigma}}^{re}\right)^{T} = -{\boldsymbol{\mathcal{H}}_{AH}^{\sigma}}^{re}$ and ${\boldsymbol{\mathcal{H}}_{AH}^{\sigma}}^{im}$ is a real symmetric matrix obeying $\left({\boldsymbol{\mathcal{H}}_{AH}^{\sigma}}^{im}\right)^{T} = {\boldsymbol{\mathcal{H}}_{AH}^{\sigma}}^{im}$. To rationalize this choice, we now plug Eq. (S12) in Eq. (S11) to obtain:

$$\dot{\boldsymbol{\mathcal{P}}}^{\sigma} = -i\boldsymbol{\mathcal{S}}^{-1}\boldsymbol{\mathcal{H}}_{KS}^{\sigma}\boldsymbol{\mathcal{P}}^{\sigma} + i\boldsymbol{\mathcal{P}}^{\sigma}\boldsymbol{\mathcal{H}}_{KS}^{\sigma}\boldsymbol{\mathcal{S}}^{-1} - i\boldsymbol{\mathcal{S}}^{-1}\boldsymbol{\mathcal{H}}_{AH}^{\sigma}\boldsymbol{\mathcal{P}}^{\sigma} - i\boldsymbol{\mathcal{P}}^{\sigma}\boldsymbol{\mathcal{H}}_{AH}^{\sigma}\boldsymbol{\mathcal{S}}^{-1} = -i\boldsymbol{\mathcal{S}}^{-1}(\boldsymbol{\mathcal{H}}_{KS}^{\sigma} + \boldsymbol{\mathcal{H}}_{AH}^{\sigma})\boldsymbol{\mathcal{P}}^{\sigma} + i\boldsymbol{\mathcal{P}}^{\sigma}(\boldsymbol{\mathcal{H}}_{KS}^{\sigma} - \boldsymbol{\mathcal{H}}_{AH}^{\sigma})\boldsymbol{\mathcal{S}}^{-1} = -i\boldsymbol{\mathcal{S}}^{-1}(\boldsymbol{\mathcal{H}}_{KS}^{\sigma} + \boldsymbol{\mathcal{H}}_{AH}^{\sigma})\boldsymbol{\mathcal{P}}^{\sigma} + i\boldsymbol{\mathcal{P}}^{\sigma}(\boldsymbol{\mathcal{H}}_{KS}^{\sigma} + \boldsymbol{\mathcal{H}}_{AH}^{\sigma})^{\dagger}\boldsymbol{\mathcal{S}}^{-1}. \tag{S13}$$

This equation assumes the form of a Liouville–von Neumann equation for a microcanonical (or canonical) system but with a general Hamiltonian matrix, $\boldsymbol{\mathcal{H}}_{KS}^{\sigma} + \boldsymbol{\mathcal{H}}_{AH}^{\sigma}$, that is neither Hermitian nor

anti-Hermitian. The latter can be viewed as a dressed Hamiltonian, where we identify $\boldsymbol{\mathcal{H}}_{AH}^{\sigma}$ as a self-energy like term representing the effects of the coupling of the system to an implicit bath. Note, however, that $\boldsymbol{\mathcal{H}}_{AH}^{\sigma}$ is energy independent and hence should be viewed as an approximation of the self-energy within the wide band limit.

To obtain the explicit expression of $\boldsymbol{\mathcal{H}}_{AH}^{\sigma}$ within the DLvN EOM we divide the system into three sections comprising of the left lead, the (extended-)molecule, and the right lead. $\boldsymbol{\mathcal{H}}_{AH}^{\sigma}$ then serves to mimic the effect of coupling of the lead sections to implicit Fermionic baths, characterized by equilibrium Fermi-Dirac distributions with given chemical potentials and electronic temperatures. To this end, we first neglect ${\boldsymbol{\mathcal{H}}_{AH}^{\sigma}}^{re}$, which is equivalent to neglecting the real-part of the implicit baths' self-energies that induce lead level shifts due to the lead/implicit-bath couplings. This approximation becomes valid for sufficiently large lead models, with a relatively uniform and dense manifold of states, such that the level shifts become small with respect to the inter-level spacing. The remaining imaginary part, ${\boldsymbol{\mathcal{H}}_{AH}^{\sigma}}^{im}$, marked for brevity as $\boldsymbol{\Gamma}^{\sigma}$, introduces a finite lifetime (broadening) to the various lead levels due to their coupling to the implicit single-particle states of the reservoir. Hence, within the DLvN approach, the dressed KS Hamiltonian acquires the form:

$$\boldsymbol{\mathcal{H}}_{KS}^{\sigma} \rightarrow \boldsymbol{\mathcal{H}}_{KS}^{\sigma} - i\boldsymbol{\Gamma}^{\sigma}. \tag{S14}$$

In this form, $\boldsymbol{\Gamma}$ can be identified as an imaginary absorbing potential added to the lead sections of the original KS system, serving to absorb outgoing electrons near the system boundaries (thus preventing their back-reflection into the system). Naturally, to avoid complete electronic depletion of the system, complementary emitting potentials should also be introduced for injecting thermalized electrons into the system, as shown below.

Using the dressed Hamiltonian form of Eq. (S14) in Eq. (S13) we obtain:

$$\begin{aligned}\dot{\boldsymbol{\mathcal{P}}}^{\sigma} &= -i\boldsymbol{\mathcal{S}}^{-1}(\boldsymbol{\mathcal{H}}_{KS}^{\sigma} - i\boldsymbol{\Gamma}^{\sigma})\boldsymbol{\mathcal{P}}^{\sigma} + i\boldsymbol{\mathcal{P}}^{\sigma}(\boldsymbol{\mathcal{H}}_{KS}^{\sigma} - i\boldsymbol{\Gamma}^{\sigma})^{\dagger}\boldsymbol{\mathcal{S}}^{-1} \\ &= -i\boldsymbol{\mathcal{S}}^{-1}\boldsymbol{\mathcal{H}}_{KS}^{\sigma}\boldsymbol{\mathcal{P}}^{\sigma} + i\boldsymbol{\mathcal{P}}^{\sigma}{\boldsymbol{\mathcal{H}}_{KS}^{\sigma}}^{\dagger}\boldsymbol{\mathcal{S}}^{-1} - \boldsymbol{\mathcal{S}}^{-1}\boldsymbol{\Gamma}^{\sigma}\boldsymbol{\mathcal{P}}^{\sigma} - \boldsymbol{\mathcal{P}}^{\sigma}{\boldsymbol{\Gamma}^{\sigma}}^{\dagger}\boldsymbol{\mathcal{S}}^{-1} \\ &= -i\boldsymbol{\mathcal{S}}^{-1}\boldsymbol{\mathcal{H}}_{KS}^{\sigma}\boldsymbol{\mathcal{P}}^{\sigma} + i\boldsymbol{\mathcal{P}}^{\sigma}\boldsymbol{\mathcal{H}}_{KS}^{\sigma}\boldsymbol{\mathcal{S}}^{-1} - \boldsymbol{\mathcal{S}}^{-1}\boldsymbol{\Gamma}^{\sigma}\boldsymbol{\mathcal{P}}^{\sigma} - \boldsymbol{\mathcal{P}}^{\sigma}{\boldsymbol{\Gamma}^{\sigma}}^{\dagger}\boldsymbol{\mathcal{S}}^{-1}.\end{aligned} \tag{S15}$$

We can now multiply Eq. (S15) by $\boldsymbol{\mathcal{S}}$ from left and from right to obtain:

$$\boldsymbol{\mathcal{S}}\dot{\boldsymbol{\mathcal{P}}}^{\sigma}\boldsymbol{\mathcal{S}} = -i\boldsymbol{\mathcal{H}}_{KS}^{\sigma}\boldsymbol{\mathcal{P}}^{\sigma}\boldsymbol{\mathcal{S}} + i\boldsymbol{\mathcal{S}}\boldsymbol{\mathcal{P}}^{\sigma}\boldsymbol{\mathcal{H}}_{KS}^{\sigma} - \boldsymbol{\Gamma}^{\sigma}\boldsymbol{\mathcal{P}}^{\sigma}\boldsymbol{\mathcal{S}} - \boldsymbol{\mathcal{S}}\boldsymbol{\mathcal{P}}^{\sigma}{\boldsymbol{\Gamma}^{\sigma}}^{\dagger}. \tag{S16}$$

Next, we introduce a block diagonalization transformation, $\boldsymbol{U}_b$, into Eq. (S16) to nullify the off-diagonal overlap matrix blocks as follows:[1]

$$\left[\left(\boldsymbol{U}_b^{\dagger}\right)^{-1}\boldsymbol{U}_b^{\dagger}\right]\boldsymbol{\mathcal{S}}[\boldsymbol{U}_b\boldsymbol{U}_b^{-1}]\dot{\boldsymbol{\mathcal{P}}}^{\sigma}\left[\left(\boldsymbol{U}_b^{\dagger}\right)^{-1}\boldsymbol{U}_b^{\dagger}\right]\boldsymbol{\mathcal{S}}[\boldsymbol{U}_b\boldsymbol{U}_b^{-1}] =$$

$$= -i\left[\left(\boldsymbol{U}_b^{\dagger}\right)^{-1}\boldsymbol{U}_b^{\dagger}\right]\boldsymbol{\mathcal{H}}_{KS}^{\sigma}[\boldsymbol{U}_b\boldsymbol{U}_b^{-1}]\boldsymbol{\mathcal{P}}^{\sigma}\left[\left(\boldsymbol{U}_b^{\dagger}\right)^{-1}\boldsymbol{U}_b^{\dagger}\right]\boldsymbol{\mathcal{S}}[\boldsymbol{U}_b\boldsymbol{U}_b^{-1}] +$$

$$+i\left[\left(\boldsymbol{U}_b^{\dagger}\right)^{-1}\boldsymbol{U}_b^{\dagger}\right]\boldsymbol{\mathcal{S}}[\boldsymbol{U}_b\boldsymbol{U}_b^{-1}]\boldsymbol{\mathcal{P}}^{\sigma}\left[\left(\boldsymbol{U}_b^{\dagger}\right)^{-1}\boldsymbol{U}_b^{\dagger}\right]\boldsymbol{\mathcal{H}}_{KS}^{\sigma}[\boldsymbol{U}_b\boldsymbol{U}_b^{-1}] - \boldsymbol{\Gamma}^{\sigma}\boldsymbol{\mathcal{P}}^{\sigma}\left[\left(\boldsymbol{U}_b^{\dagger}\right)^{-1}\boldsymbol{U}_b^{\dagger}\right]\boldsymbol{\mathcal{S}}[\boldsymbol{U}_b\boldsymbol{U}_b^{-1}] -$$

$$\left[\left(\boldsymbol{U}_b^{\dagger}\right)^{-1}\boldsymbol{U}_b^{\dagger}\right]\boldsymbol{\mathcal{S}}[\boldsymbol{U}_b\boldsymbol{U}_b^{-1}]\boldsymbol{\mathcal{P}}^{\sigma}\boldsymbol{\Gamma}^{\sigma\dagger}, \tag{S17}$$

where

$$\boldsymbol{U}_b \equiv \begin{pmatrix} \boldsymbol{I}_L & -\boldsymbol{S}_L^{-1}\boldsymbol{S}_{L,EM} & \boldsymbol{0} \\ \boldsymbol{0} & \boldsymbol{I}_{EM} & \boldsymbol{0} \\ \boldsymbol{0} & -\boldsymbol{S}_R^{-1}\boldsymbol{S}_{R,EM} & \boldsymbol{I}_R \end{pmatrix};\ \boldsymbol{U}_b^{-1} = \begin{pmatrix} \boldsymbol{I}_L & \boldsymbol{S}_L^{-1}\boldsymbol{S}_{L,EM} & \boldsymbol{0} \\ \boldsymbol{0} & \boldsymbol{I}_{EM} & \boldsymbol{0} \\ \boldsymbol{0} & \boldsymbol{S}_R^{-1}\boldsymbol{S}_{R,EM} & \boldsymbol{I}_R \end{pmatrix}. \tag{S18}$$

Defining

$$\begin{cases} \widetilde{\boldsymbol{\mathcal{S}}} \equiv \boldsymbol{U}_b^{\dagger}\boldsymbol{\mathcal{S}}\boldsymbol{U}_b \\ \widetilde{\boldsymbol{\mathcal{H}}}_{KS}^{\sigma} \equiv \boldsymbol{U}_b^{\dagger}\boldsymbol{\mathcal{H}}_{KS}^{\sigma}\boldsymbol{U}_b \end{cases} \tag{S19}$$

Eq. (S17) can be rewritten as follows:

$$\left(\boldsymbol{U}_b^{\dagger}\right)^{-1}\widetilde{\boldsymbol{\mathcal{S}}}\boldsymbol{U}_b^{-1}\dot{\boldsymbol{\mathcal{P}}}^{\sigma}\left(\boldsymbol{U}_b^{\dagger}\right)^{-1}\widetilde{\boldsymbol{\mathcal{S}}}\boldsymbol{U}_b^{-1} = -i\left(\boldsymbol{U}_b^{\dagger}\right)^{-1}\widetilde{\boldsymbol{\mathcal{H}}}_{KS}^{\sigma}\boldsymbol{U}_b^{-1}\boldsymbol{\mathcal{P}}^{\sigma}\left(\boldsymbol{U}_b^{\dagger}\right)^{-1}\widetilde{\boldsymbol{\mathcal{S}}}\boldsymbol{U}_b^{-1} +$$

$$i\left(\boldsymbol{U}_b^{\dagger}\right)^{-1}\widetilde{\boldsymbol{\mathcal{S}}}\boldsymbol{U}_b^{-1}\boldsymbol{\mathcal{P}}^{\sigma}\left(\boldsymbol{U}_b^{\dagger}\right)^{-1}\widetilde{\boldsymbol{\mathcal{H}}}_{KS}^{\sigma}\boldsymbol{U}_b^{-1} - \boldsymbol{\Gamma}^{\sigma}\boldsymbol{\mathcal{P}}^{\sigma}\left(\boldsymbol{U}_b^{\dagger}\right)^{-1}\widetilde{\boldsymbol{\mathcal{S}}}\boldsymbol{U}_b^{-1} - \left(\boldsymbol{U}_b^{\dagger}\right)^{-1}\widetilde{\boldsymbol{\mathcal{S}}}\boldsymbol{U}_b^{-1}\boldsymbol{\mathcal{P}}^{\sigma}\boldsymbol{\Gamma}^{\sigma\dagger}. \tag{S20}$$

Next, we multiply Eq. (S20) by $\boldsymbol{U}_b^{\dagger}$ on the left and by $\boldsymbol{U}_b$ on the right, to obtain:

$$\widetilde{\boldsymbol{\mathcal{S}}}\boldsymbol{U}_b^{-1}\dot{\boldsymbol{\mathcal{P}}}^{\sigma}\left(\boldsymbol{U}_b^{\dagger}\right)^{-1}\widetilde{\boldsymbol{\mathcal{S}}} =$$

$$= -i\widetilde{\boldsymbol{\mathcal{H}}}_{KS}^{\sigma}\boldsymbol{U}_b^{-1}\boldsymbol{\mathcal{P}}^{\sigma}\left(\boldsymbol{U}_b^{\dagger}\right)^{-1}\widetilde{\boldsymbol{\mathcal{S}}} + i\widetilde{\boldsymbol{\mathcal{S}}}\boldsymbol{U}_b^{-1}\boldsymbol{\mathcal{P}}^{\sigma}\left(\boldsymbol{U}_b^{\dagger}\right)^{-1}\widetilde{\boldsymbol{\mathcal{H}}}_{KS}^{\sigma} - \boldsymbol{U}_b^{\dagger}\boldsymbol{\Gamma}^{\sigma}\boldsymbol{\mathcal{P}}^{\sigma}\left(\boldsymbol{U}_b^{\dagger}\right)^{-1}\widetilde{\boldsymbol{\mathcal{S}}} - \widetilde{\boldsymbol{\mathcal{S}}}\boldsymbol{U}_b^{-1}\boldsymbol{\mathcal{P}}^{\sigma}\boldsymbol{\Gamma}^{\sigma\dagger}\boldsymbol{U}_b. \tag{S21}$$

Introducing $\boldsymbol{I} = \boldsymbol{U}_b\boldsymbol{U}_b^{-1}$ and $\boldsymbol{I} = \left(\boldsymbol{U}_b^{\dagger}\right)^{-1}\boldsymbol{U}_b^{\dagger}$ in the last two terms, respectively, yields:

$$\widetilde{\boldsymbol{\mathcal{S}}}\boldsymbol{U}_b^{-1}\dot{\boldsymbol{\mathcal{P}}}^{\sigma}\left(\boldsymbol{U}_b^{\dagger}\right)^{-1}\widetilde{\boldsymbol{\mathcal{S}}} = -i\widetilde{\boldsymbol{\mathcal{H}}}_{KS}^{\sigma}\boldsymbol{U}_b^{-1}\boldsymbol{\mathcal{P}}^{\sigma}\left(\boldsymbol{U}_b^{\dagger}\right)^{-1}\widetilde{\boldsymbol{\mathcal{S}}} + i\widetilde{\boldsymbol{\mathcal{S}}}\boldsymbol{U}_b^{-1}\boldsymbol{\mathcal{P}}^{\sigma}\left(\boldsymbol{U}_b^{\dagger}\right)^{-1}\widetilde{\boldsymbol{\mathcal{H}}}_{KS}^{\sigma} - \boldsymbol{U}_b^{\dagger}\boldsymbol{\Gamma}^{\sigma}\boldsymbol{U}_b\boldsymbol{U}_b^{-1}\boldsymbol{\mathcal{P}}^{\sigma}\left(\boldsymbol{U}_b^{\dagger}\right)^{-1}\widetilde{\boldsymbol{\mathcal{S}}} -$$

$$\widetilde{\boldsymbol{\mathcal{S}}}\boldsymbol{U}_b^{-1}\boldsymbol{\mathcal{P}}^{\sigma}\left(\boldsymbol{U}_b^{\dagger}\right)^{-1}\boldsymbol{U}_b^{\dagger}\boldsymbol{\Gamma}^{\sigma\dagger}\boldsymbol{U}_b. \tag{S22}$$

Next, we define:

$$\begin{cases} \widetilde{\boldsymbol{\mathcal{P}}}^{\sigma} \equiv \boldsymbol{U}_b^{-1}\boldsymbol{\mathcal{P}}^{\sigma}\left(\boldsymbol{U}_b^{\dagger}\right)^{-1} \\ \widetilde{\dot{\boldsymbol{\mathcal{P}}}}^{\sigma} \equiv \boldsymbol{U}_b^{-1}\dot{\boldsymbol{\mathcal{P}}}^{\sigma}\left(\boldsymbol{U}_b^{\dagger}\right)^{-1} \\ \widetilde{\boldsymbol{\Gamma}}^{\sigma} \equiv \boldsymbol{U}_b^{\dagger}\boldsymbol{\Gamma}^{\sigma}\boldsymbol{U}_b \end{cases}. \tag{S23}$$

We note that since $\boldsymbol{U}_b$ is a fixed transformation (time-independent within the fixed nuclei Born-Oppenheimer approximation) the relation $\dot{\widetilde{\boldsymbol{\mathcal{P}}}}^{\sigma} = \widetilde{\dot{\boldsymbol{\mathcal{P}}}}^{\sigma}$ holds. With these definitions we obtain:

$$\widetilde{\boldsymbol{\mathcal{S}}}\dot{\widetilde{\boldsymbol{\mathcal{P}}}}^{\sigma}\widetilde{\boldsymbol{\mathcal{S}}} = -i\widetilde{\boldsymbol{\mathcal{H}}}_{KS}^{\sigma}\widetilde{\boldsymbol{\mathcal{P}}}^{\sigma}\widetilde{\boldsymbol{\mathcal{S}}} + i\widetilde{\boldsymbol{\mathcal{S}}}\widetilde{\boldsymbol{\mathcal{P}}}^{\sigma}\widetilde{\boldsymbol{\mathcal{H}}}_{KS}^{\sigma} - \widetilde{\boldsymbol{\Gamma}}^{\sigma}\widetilde{\boldsymbol{\mathcal{P}}}^{\sigma}\widetilde{\boldsymbol{\mathcal{S}}} - \widetilde{\boldsymbol{\mathcal{S}}}\widetilde{\boldsymbol{\mathcal{P}}}^{\sigma}\widetilde{\boldsymbol{\Gamma}}^{\sigma\dagger}, \tag{S24}$$

where we have used the fact that $\widetilde{\boldsymbol{\Gamma}}^{\sigma\dagger} = \left(\boldsymbol{U}_b^{\dagger}\boldsymbol{\Gamma}^{\sigma}\boldsymbol{U}_b\right)^{\dagger} = \boldsymbol{U}_b^{\dagger}\,\boldsymbol{\Gamma}^{\sigma\dagger}\boldsymbol{U}_b$.

Next, we introduce the site-to-state transformation:

$$
\boldsymbol{U}^{\sigma} \equiv \begin{pmatrix} \boldsymbol{U}_L^{\sigma} & \boldsymbol{0} & \boldsymbol{0} \\ \boldsymbol{0} & \boldsymbol{U}_{EM}^{\sigma} & \boldsymbol{0} \\ \boldsymbol{0} & \boldsymbol{0} & \boldsymbol{U}_R^{\sigma} \end{pmatrix}, \tag{S25}
$$

such that $\tilde{\tilde{\boldsymbol{H}}}_{KS_i}^{\sigma} = \boldsymbol{U}_i^{\sigma\dagger}\tilde{\boldsymbol{H}}_{KS_i}^{\sigma}\boldsymbol{U}_i^{\sigma}$ is diagonal and $\boldsymbol{U}_i^{\sigma\dagger}\tilde{\boldsymbol{S}}_i\boldsymbol{U}_i^{\sigma} = \boldsymbol{I}_i$ are unit submatrices of the appropriate dimensions. With this, Eq. (S24) can be rewritten as:

$$
\begin{aligned}
&\left(\boldsymbol{U}^{\sigma\dagger}\right)^{-1}\boldsymbol{U}^{\sigma\dagger}\tilde{\boldsymbol{\mathcal{S}}}\boldsymbol{U}^{\sigma}(\boldsymbol{U}^{\sigma})^{-1}\tilde{\dot{\boldsymbol{\mathcal{P}}}}^{\sigma}\left(\boldsymbol{U}^{\sigma\dagger}\right)^{-1}\boldsymbol{U}^{\sigma\dagger}\tilde{\boldsymbol{\mathcal{S}}}\boldsymbol{U}^{\sigma}(\boldsymbol{U}^{\sigma})^{-1} = \\
&= -i\left(\boldsymbol{U}^{\sigma\dagger}\right)^{-1}\boldsymbol{U}^{\sigma\dagger}\widetilde{\boldsymbol{\mathcal{H}}}_{KS}^{\sigma}\boldsymbol{U}^{\sigma}(\boldsymbol{U}^{\sigma})^{-1}\tilde{\boldsymbol{\mathcal{P}}}^{\sigma}\left(\boldsymbol{U}^{\sigma\dagger}\right)^{-1}\boldsymbol{U}^{\sigma\dagger}\tilde{\boldsymbol{\mathcal{S}}}\boldsymbol{U}^{\sigma}(\boldsymbol{U}^{\sigma})^{-1} + \\
&i\left(\boldsymbol{U}^{\sigma\dagger}\right)^{-1}\boldsymbol{U}^{\sigma\dagger}\tilde{\boldsymbol{\mathcal{S}}}\boldsymbol{U}^{\sigma}(\boldsymbol{U}^{\sigma})^{-1}\tilde{\boldsymbol{\mathcal{P}}}^{\sigma}\left(\boldsymbol{U}^{\sigma\dagger}\right)^{-1}\boldsymbol{U}^{\sigma\dagger}\widetilde{\boldsymbol{\mathcal{H}}}_{KS}^{\sigma}\boldsymbol{U}^{\sigma}(\boldsymbol{U}^{\sigma})^{-1} - \\
&\tilde{\boldsymbol{\Gamma}}^{\sigma}\boldsymbol{U}^{\sigma}(\boldsymbol{U}^{\sigma})^{-1}\tilde{\boldsymbol{\mathcal{P}}}^{\sigma}\left(\boldsymbol{U}^{\sigma\dagger}\right)^{-1}\boldsymbol{U}^{\sigma\dagger}\tilde{\boldsymbol{\mathcal{S}}}\boldsymbol{U}^{\sigma}(\boldsymbol{U}^{\sigma})^{-1} - \left(\boldsymbol{U}^{\sigma\dagger}\right)^{-1}\boldsymbol{U}^{\sigma\dagger}\tilde{\boldsymbol{\mathcal{S}}}\boldsymbol{U}^{\sigma}(\boldsymbol{U}^{\sigma})^{-1}\tilde{\boldsymbol{\mathcal{P}}}^{\sigma}\left(\boldsymbol{U}^{\sigma\dagger}\right)^{-1}\boldsymbol{U}^{\sigma\dagger}\tilde{\boldsymbol{\Gamma}}^{\sigma\dagger}
\end{aligned} \tag{S26}
$$

Using the relation $\boldsymbol{U}^{\sigma\dagger}\tilde{\boldsymbol{\mathcal{S}}}\boldsymbol{U}^{\sigma} = \boldsymbol{I}$ we get:

$$
\begin{aligned}
&\left(\boldsymbol{U}^{\sigma\dagger}\right)^{-1}(\boldsymbol{U}^{\sigma})^{-1}\tilde{\dot{\boldsymbol{\mathcal{P}}}}^{\sigma}\left(\boldsymbol{U}^{\sigma\dagger}\right)^{-1}(\boldsymbol{U}^{\sigma})^{-1} = \\
&= -i\left(\boldsymbol{U}^{\sigma\dagger}\right)^{-1}\boldsymbol{U}^{\sigma\dagger}\widetilde{\boldsymbol{\mathcal{H}}}_{KS}^{\sigma}\boldsymbol{U}^{\sigma}(\boldsymbol{U}^{\sigma})^{-1}\tilde{\boldsymbol{\mathcal{P}}}^{\sigma}\left(\boldsymbol{U}^{\sigma\dagger}\right)^{-1}(\boldsymbol{U}^{\sigma})^{-1} + \\
&i\left(\boldsymbol{U}^{\sigma\dagger}\right)^{-1}(\boldsymbol{U}^{\sigma})^{-1}\tilde{\boldsymbol{\mathcal{P}}}^{\sigma}\left(\boldsymbol{U}^{\sigma\dagger}\right)^{-1}\boldsymbol{U}^{\sigma\dagger}\widetilde{\boldsymbol{\mathcal{H}}}_{KS}^{\sigma}\boldsymbol{U}^{\sigma}(\boldsymbol{U}^{\sigma})^{-1} - \tilde{\boldsymbol{\Gamma}}^{\sigma}\boldsymbol{U}^{\sigma}(\boldsymbol{U}^{\sigma})^{-1}\tilde{\boldsymbol{\mathcal{P}}}^{\sigma}\left(\boldsymbol{U}^{\sigma\dagger}\right)^{-1}(\boldsymbol{U}^{\sigma})^{-1} - \\
&\left(\boldsymbol{U}^{\sigma\dagger}\right)^{-1}(\boldsymbol{U}^{\sigma})^{-1}\tilde{\boldsymbol{\mathcal{P}}}^{\sigma}\left(\boldsymbol{U}^{\sigma\dagger}\right)^{-1}\boldsymbol{U}^{\sigma\dagger}\tilde{\boldsymbol{\Gamma}}^{\sigma\dagger}
\end{aligned} \tag{S27}
$$

Next, we define:

$$
\begin{cases}
\widetilde{\widetilde{\boldsymbol{\mathcal{H}}}}_{KS}^{\sigma} \equiv \boldsymbol{U}^{\sigma\dagger}\widetilde{\boldsymbol{\mathcal{H}}}_{KS}^{\sigma}\boldsymbol{U}^{\sigma} \\
\tilde{\tilde{\boldsymbol{\mathcal{P}}}}^{\sigma} \equiv (\boldsymbol{U}^{\sigma})^{-1}\tilde{\boldsymbol{\mathcal{P}}}^{\sigma}\left(\boldsymbol{U}^{\sigma\dagger}\right)^{-1}, \\
\tilde{\tilde{\dot{\boldsymbol{\mathcal{P}}}}}^{\sigma} \equiv (\boldsymbol{U}^{\sigma})^{-1}\tilde{\dot{\boldsymbol{\mathcal{P}}}}^{\sigma}\left(\boldsymbol{U}^{\sigma\dagger}\right)^{-1}
\end{cases} \tag{S28}
$$

to obtain:

$$
\begin{aligned}
&\left(\boldsymbol{U}^{\sigma\dagger}\right)^{-1}\tilde{\tilde{\dot{\boldsymbol{\mathcal{P}}}}}^{\sigma}(\boldsymbol{U}^{\sigma})^{-1} = -i\left(\boldsymbol{U}^{\sigma\dagger}\right)^{-1}\widetilde{\widetilde{\boldsymbol{\mathcal{H}}}}_{KS}^{\sigma}\tilde{\tilde{\boldsymbol{\mathcal{P}}}}^{\sigma}(\boldsymbol{U}^{\sigma})^{-1} + i\left(\boldsymbol{U}^{\sigma\dagger}\right)^{-1}\tilde{\tilde{\boldsymbol{\mathcal{P}}}}^{\sigma}\widetilde{\widetilde{\boldsymbol{\mathcal{H}}}}_{KS}^{\sigma}(\boldsymbol{U}^{\sigma})^{-1} - \tilde{\boldsymbol{\Gamma}}^{\sigma}\boldsymbol{U}^{\sigma}\tilde{\tilde{\boldsymbol{\mathcal{P}}}}^{\sigma}(\boldsymbol{U}^{\sigma})^{-1} - \\
&\left(\boldsymbol{U}^{\sigma\dagger}\right)^{-1}\tilde{\tilde{\boldsymbol{\mathcal{P}}}}^{\sigma}\boldsymbol{U}^{\sigma\dagger}\tilde{\boldsymbol{\Gamma}}^{\sigma\dagger}.
\end{aligned} \tag{S29}
$$

Multiplying by $\boldsymbol{U}^{\sigma\dagger}$ on the left and $\boldsymbol{U}^{\boldsymbol{\sigma}}$ on the right we arrive at:

$$
\tilde{\tilde{\dot{\boldsymbol{\mathcal{P}}}}}^{\sigma} = -i\widetilde{\widetilde{\boldsymbol{\mathcal{H}}}}_{KS}^{\sigma}\tilde{\tilde{\boldsymbol{\mathcal{P}}}}^{\sigma} + i\tilde{\tilde{\boldsymbol{\mathcal{P}}}}^{\sigma}\widetilde{\widetilde{\boldsymbol{\mathcal{H}}}}_{KS}^{\sigma} - \boldsymbol{U}^{\sigma\dagger}\tilde{\boldsymbol{\Gamma}}^{\sigma}\boldsymbol{U}^{\sigma}\tilde{\tilde{\boldsymbol{\mathcal{P}}}}^{\sigma} - \tilde{\tilde{\boldsymbol{\mathcal{P}}}}^{\sigma}\boldsymbol{U}^{\sigma\dagger}\tilde{\boldsymbol{\Gamma}}^{\sigma\dagger}\boldsymbol{U}^{\sigma}. \tag{S30}
$$

Defining:

$$
\tilde{\tilde{\boldsymbol{\Gamma}}}^{\sigma} \equiv \boldsymbol{U}^{\sigma\dagger}\tilde{\boldsymbol{\Gamma}}^{\sigma}\boldsymbol{U}^{\sigma}, \tag{S31}
$$

we finally obtain:

$$\dot{\tilde{\tilde{\boldsymbol{\mathcal{P}}}}}^{\sigma} = -i\left[\tilde{\tilde{\boldsymbol{\mathcal{H}}}}_{KS}^{\sigma}, \tilde{\tilde{\boldsymbol{\mathcal{P}}}}^{\sigma}\right] - \tilde{\tilde{\boldsymbol{\Gamma}}}^{\sigma}\tilde{\tilde{\boldsymbol{\mathcal{P}}}}^{\sigma} - \tilde{\tilde{\boldsymbol{\mathcal{P}}}}^{\sigma}\tilde{\tilde{\boldsymbol{\Gamma}}}^{\sigma\dagger}, \tag{S32}$$

where $\tilde{\tilde{\boldsymbol{\Gamma}}}^{\sigma\dagger} = \left(\boldsymbol{U}^{\sigma\dagger}\tilde{\boldsymbol{\Gamma}}^{\sigma}\boldsymbol{U}^{\sigma}\right)^{\dagger} = \boldsymbol{U}^{\sigma\dagger}\tilde{\boldsymbol{\Gamma}}^{\sigma\dagger}\boldsymbol{U}^{\sigma}$. In its simplest form $\tilde{\tilde{\boldsymbol{\Gamma}}}^{\sigma}$ is written as:

$$\tilde{\tilde{\boldsymbol{\Gamma}}}^{\sigma} = \tilde{\tilde{\boldsymbol{\Gamma}}}^{\sigma\dagger} = \gamma^{\sigma}\begin{pmatrix} \boldsymbol{I}_L & \boldsymbol{0} & \boldsymbol{0} \\ \boldsymbol{0} & \boldsymbol{0} & \boldsymbol{0} \\ \boldsymbol{0} & \boldsymbol{0} & \boldsymbol{I}_R \end{pmatrix}, \tag{S33}$$

which represents uniform broadening of all left and right lead levels. Hence, the last two terms in Eq. (S32) can be written as:

$$-\gamma^{\sigma}\begin{pmatrix} \boldsymbol{I}_L & \boldsymbol{0} & \boldsymbol{0} \\ \boldsymbol{0} & \boldsymbol{0} & \boldsymbol{0} \\ \boldsymbol{0} & \boldsymbol{0} & \boldsymbol{I}_R \end{pmatrix}\tilde{\tilde{\boldsymbol{\mathcal{P}}}}^{\sigma} - \gamma^{\sigma}\tilde{\tilde{\boldsymbol{\mathcal{P}}}}^{\sigma}\begin{pmatrix} \boldsymbol{I}_L & \boldsymbol{0} & \boldsymbol{0} \\ \boldsymbol{0} & \boldsymbol{0} & \boldsymbol{0} \\ \boldsymbol{0} & \boldsymbol{0} & \boldsymbol{I}_R \end{pmatrix} =$$

$$= -\gamma^{\sigma}\begin{pmatrix} \boldsymbol{I}_L & \boldsymbol{0} & \boldsymbol{0} \\ \boldsymbol{0} & \boldsymbol{0} & \boldsymbol{0} \\ \boldsymbol{0} & \boldsymbol{0} & \boldsymbol{I}_R \end{pmatrix}\begin{pmatrix} \tilde{\tilde{\boldsymbol{\mathcal{P}}}}_{L}^{\sigma} & \tilde{\tilde{\boldsymbol{\mathcal{P}}}}_{L,EM}^{\sigma} & \tilde{\tilde{\boldsymbol{\mathcal{P}}}}_{LR}^{\sigma} \\ \tilde{\tilde{\boldsymbol{\mathcal{P}}}}_{EM,L}^{\sigma} & \tilde{\tilde{\boldsymbol{\mathcal{P}}}}_{EM}^{\sigma} & \tilde{\tilde{\boldsymbol{\mathcal{P}}}}_{EM,R}^{\sigma} \\ \tilde{\tilde{\boldsymbol{\mathcal{P}}}}_{RL}^{\sigma} & \tilde{\tilde{\boldsymbol{\mathcal{P}}}}_{R,EM}^{\sigma} & \tilde{\tilde{\boldsymbol{\mathcal{P}}}}_{R}^{\sigma} \end{pmatrix} - \gamma^{\sigma}\begin{pmatrix} \tilde{\tilde{\boldsymbol{\mathcal{P}}}}_{L}^{\sigma} & \tilde{\tilde{\boldsymbol{\mathcal{P}}}}_{L,EM}^{\sigma} & \tilde{\tilde{\boldsymbol{\mathcal{P}}}}_{LR}^{\sigma} \\ \tilde{\tilde{\boldsymbol{\mathcal{P}}}}_{EM,L}^{\sigma} & \tilde{\tilde{\boldsymbol{\mathcal{P}}}}_{EM}^{\sigma} & \tilde{\tilde{\boldsymbol{\mathcal{P}}}}_{EM,R}^{\sigma} \\ \tilde{\tilde{\boldsymbol{\mathcal{P}}}}_{RL}^{\sigma} & \tilde{\tilde{\boldsymbol{\mathcal{P}}}}_{R,EM}^{\sigma} & \tilde{\tilde{\boldsymbol{\mathcal{P}}}}_{R}^{\sigma} \end{pmatrix}\begin{pmatrix} \boldsymbol{I}_L & \boldsymbol{0} & \boldsymbol{0} \\ \boldsymbol{0} & \boldsymbol{0} & \boldsymbol{0} \\ \boldsymbol{0} & \boldsymbol{0} & \boldsymbol{I}_R \end{pmatrix} =$$

$$= -\gamma^{\sigma}\begin{pmatrix} \tilde{\tilde{\boldsymbol{\mathcal{P}}}}_{L}^{\sigma} & \tilde{\tilde{\boldsymbol{\mathcal{P}}}}_{L,EM}^{\sigma} & \tilde{\tilde{\boldsymbol{\mathcal{P}}}}_{LR}^{\sigma} \\ \boldsymbol{0} & \boldsymbol{0} & \boldsymbol{0} \\ \tilde{\tilde{\boldsymbol{\mathcal{P}}}}_{RL}^{\sigma} & \tilde{\tilde{\boldsymbol{\mathcal{P}}}}_{R,EM}^{\sigma} & \tilde{\tilde{\boldsymbol{\mathcal{P}}}}_{R}^{\sigma} \end{pmatrix} - \gamma^{\sigma}\begin{pmatrix} \tilde{\tilde{\boldsymbol{\mathcal{P}}}}_{L}^{\sigma} & \boldsymbol{0} & \tilde{\tilde{\boldsymbol{\mathcal{P}}}}_{LR}^{\sigma} \\ \tilde{\tilde{\boldsymbol{\mathcal{P}}}}_{EM,L}^{\sigma} & \boldsymbol{0} & \tilde{\tilde{\boldsymbol{\mathcal{P}}}}_{EM,R}^{\sigma} \\ \tilde{\tilde{\boldsymbol{\mathcal{P}}}}_{RL}^{\sigma} & \boldsymbol{0} & \tilde{\tilde{\boldsymbol{\mathcal{P}}}}_{R}^{\sigma} \end{pmatrix} = -\gamma^{\sigma}\begin{pmatrix} 2\tilde{\tilde{\boldsymbol{\mathcal{P}}}}_{L}^{\sigma} & \tilde{\tilde{\boldsymbol{\mathcal{P}}}}_{L,EM}^{\sigma} & 2\tilde{\tilde{\boldsymbol{\mathcal{P}}}}_{LR}^{\sigma} \\ \tilde{\tilde{\boldsymbol{\mathcal{P}}}}_{EM,L}^{\sigma} & \boldsymbol{0} & \tilde{\tilde{\boldsymbol{\mathcal{P}}}}_{EM,R}^{\sigma} \\ 2\tilde{\tilde{\boldsymbol{\mathcal{P}}}}_{RL}^{\sigma} & \tilde{\tilde{\boldsymbol{\mathcal{P}}}}_{R,EM}^{\sigma} & 2\tilde{\tilde{\boldsymbol{\mathcal{P}}}}_{R}^{\sigma} \end{pmatrix} =$$

$$= -2\gamma^{\sigma}\begin{pmatrix} \tilde{\tilde{\boldsymbol{\mathcal{P}}}}_{L\,L}^{\sigma} & \frac{1}{2}\tilde{\tilde{\boldsymbol{\mathcal{P}}}}_{L,EM}^{\sigma} & \tilde{\tilde{\boldsymbol{\mathcal{P}}}}_{LR}^{\sigma} \\ \frac{1}{2}\tilde{\tilde{\boldsymbol{\mathcal{P}}}}_{EM,L}^{\sigma} & \boldsymbol{0} & \frac{1}{2}\tilde{\tilde{\boldsymbol{\mathcal{P}}}}_{EM,R}^{\sigma} \\ \tilde{\tilde{\boldsymbol{\mathcal{P}}}}_{RL}^{\sigma} & \frac{1}{2}\tilde{\tilde{\boldsymbol{\mathcal{P}}}}_{R,EM}^{\sigma} & \tilde{\tilde{\boldsymbol{\mathcal{P}}}}_{R}^{\sigma} \end{pmatrix}. \tag{S34}$$

The source term is then obtained by considering σ-electrons that travel from the implicit reservoirs toward the left or right leads. This is accounted for by introducing the target density matrix blocks $\tilde{\tilde{\boldsymbol{\mathcal{P}}}}_{L}^{\sigma\,0}$ and $\tilde{\tilde{\boldsymbol{\mathcal{P}}}}_{R}^{\sigma\,0}$, which are diagonal in the state representation, and hold on their diagonal the equilibrium Fermi-Dirac distributions of the implicit left and right reservoirs, $f_{L/R}\left(\varepsilon_i^{L/R^{\sigma}}, \mu_{L/R}^{\sigma}\right) = \left[e^{\left(\varepsilon_i^{L/R^{\sigma}} - \mu_{L/R}^{\sigma}\right)/\left(k_B T_{L/R}^{\sigma}\right)} + 1\right]^{-1}$, where k_B is the Boltzmann constant, $T_{L/R}^{\sigma}$ is the electronic temperature, $\varepsilon_i^{L/R^{\sigma}}$ is the ith eigenvalue of the L/R lead, and $\mu_{L/R}^{\sigma}$ is the corresponding chemical potential.

Upon reaching the reservoir/lead interface electrons are absorbed at a rate of $2\gamma^{\sigma}$ and are injected into the system at the same rate. This can be described by the following term, which is obtained from Eq. (S34) by replacing the evolving density matrix with the equilibrium density matrix of the isolated system sections and inverting the sign of γ^{σ} to account for electron injection into the system, rather than their

absorption, at the system boundaries:

$$\gamma^{\sigma}\begin{pmatrix}\boldsymbol{I}_L & \mathbf{0} & \mathbf{0}\\ \mathbf{0} & \mathbf{0} & \mathbf{0}\\ \mathbf{0} & \mathbf{0} & \boldsymbol{I}_R\end{pmatrix}\begin{pmatrix}{\tilde{\tilde{\boldsymbol{\mathcal{P}}}}_L^{\sigma}}^0 & \mathbf{0} & \mathbf{0}\\ \mathbf{0} & {\tilde{\tilde{\boldsymbol{\mathcal{P}}}}_{EM}^{\sigma}}^0 & \mathbf{0}\\ \mathbf{0} & \mathbf{0} & {\tilde{\tilde{\boldsymbol{\mathcal{P}}}}_R^{\sigma}}^0\end{pmatrix}+\gamma^{\sigma}\begin{pmatrix}{\tilde{\tilde{\boldsymbol{\mathcal{P}}}}_L^{\sigma}}^0 & \mathbf{0} & \mathbf{0}\\ \mathbf{0} & {\tilde{\tilde{\boldsymbol{\mathcal{P}}}}_{EM}^{\sigma}}^0 & \mathbf{0}\\ \mathbf{0} & \mathbf{0} & {\tilde{\tilde{\boldsymbol{\mathcal{P}}}}_R^{\sigma}}^0\end{pmatrix}\begin{pmatrix}\boldsymbol{I}_L & \mathbf{0} & \mathbf{0}\\ \mathbf{0} & \mathbf{0} & \mathbf{0}\\ \mathbf{0} & \mathbf{0} & \boldsymbol{I}_R\end{pmatrix}=$$

$$=\gamma^{\sigma}\begin{pmatrix}{\tilde{\tilde{\boldsymbol{\mathcal{P}}}}_L^{\sigma}}^0 & \mathbf{0} & \mathbf{0}\\ \mathbf{0} & \mathbf{0} & \mathbf{0}\\ \mathbf{0} & \mathbf{0} & {\tilde{\tilde{\boldsymbol{\mathcal{P}}}}_R^{\sigma}}^0\end{pmatrix}+\gamma^{\sigma}\begin{pmatrix}{\tilde{\tilde{\boldsymbol{\mathcal{P}}}}_L^{\sigma}}^0 & \mathbf{0} & \mathbf{0}\\ \mathbf{0} & \mathbf{0} & \mathbf{0}\\ \mathbf{0} & \mathbf{0} & {\tilde{\tilde{\boldsymbol{\mathcal{P}}}}_R^{\sigma}}^0\end{pmatrix}=2\gamma^{\sigma}\begin{pmatrix}{\tilde{\tilde{\boldsymbol{\mathcal{P}}}}_L^{\sigma}}^0 & \mathbf{0} & \mathbf{0}\\ \mathbf{0} & \mathbf{0} & \mathbf{0}\\ \mathbf{0} & \mathbf{0} & {\tilde{\tilde{\boldsymbol{\mathcal{P}}}}_L^{\sigma}}^0\end{pmatrix}. \tag{S35}$$

This term drives the system at the lead sections towards the equilibrium state of leads that are coupled to the corresponding external implicit reservoirs and decoupled from the extended molecule section Inserting the expressions of Eqs. (S34) and (S35) into Eq. (S32) and defining $\Gamma^{\sigma} \equiv 2\gamma^{\sigma}$ we obtain:

$$\tilde{\tilde{\dot{\boldsymbol{\mathcal{P}}}}}^{\sigma}=-i\left[\tilde{\tilde{\boldsymbol{\mathcal{H}}}}_{KS}^{\sigma},\tilde{\tilde{\boldsymbol{\mathcal{P}}}}^{\sigma}\right]-\Gamma^{\sigma}\begin{pmatrix}\tilde{\tilde{\boldsymbol{\mathcal{P}}}}_L^{\sigma}-{\tilde{\tilde{\boldsymbol{\mathcal{P}}}}_L^{\sigma}}^0 & \frac{1}{2}\tilde{\tilde{\boldsymbol{\mathcal{P}}}}_{L,EM}^{\sigma} & \tilde{\tilde{\boldsymbol{\mathcal{P}}}}_{LR}^{\sigma}\\ \frac{1}{2}\tilde{\tilde{\boldsymbol{\mathcal{P}}}}_{EM,L}^{\sigma} & \mathbf{0} & \frac{1}{2}\tilde{\tilde{\boldsymbol{\mathcal{P}}}}_{EM,R}^{\sigma}\\ \tilde{\tilde{\boldsymbol{\mathcal{P}}}}_{RL}^{\sigma} & \frac{1}{2}\tilde{\tilde{\boldsymbol{\mathcal{P}}}}_{R,EM}^{\sigma} & \tilde{\tilde{\boldsymbol{\mathcal{P}}}}_R^{\sigma}-{\tilde{\tilde{\boldsymbol{\mathcal{P}}}}_R^{\sigma}}^0\end{pmatrix}. \tag{S36}$$

Note that within the realm of spin-uncompensated TDDFT, $\tilde{\tilde{\dot{\boldsymbol{\mathcal{P}}}}}^{\sigma}$, which is the state representation of $\dot{\boldsymbol{\mathcal{P}}}^{\sigma}$, is not the time derivative of $\tilde{\tilde{\boldsymbol{\mathcal{P}}}}^{\sigma}$, namely $\tilde{\tilde{\dot{\boldsymbol{\mathcal{P}}}}}^{\sigma} \neq \dot{\tilde{\tilde{\boldsymbol{\mathcal{P}}}}}^{\sigma}$. This results from the fact that the KS Hamiltonian matrix has implicit time-dependence via its dependence on the density matrix and hence the $\boldsymbol{U}^{\sigma}$ transformation matrix varies with time as well. Since $\tilde{\tilde{\boldsymbol{\mathcal{P}}}}^{\sigma} \equiv (\boldsymbol{U}^{\sigma})^{-1}\tilde{\boldsymbol{\mathcal{P}}}^{\sigma}\left(\boldsymbol{U}^{\sigma\dagger}\right)^{-1}$ (see Eq. (S28)), its time derivative, $\dot{\tilde{\tilde{\boldsymbol{\mathcal{P}}}}}^{\sigma}$, should include the time derivative of $\boldsymbol{U}^{\sigma}$. Lacking an explicit equation of motion for $\boldsymbol{U}^{\sigma}$, we are thus forced to perform the propagation step in the site representation. To this end, we use Eqs. (S19), (S23), and (S28) within Eq. (S36) as follows:

$$(\boldsymbol{U}^{\sigma})^{-1}\boldsymbol{U}_b^{-1}\dot{\boldsymbol{\mathcal{P}}}^{\sigma}\left(\boldsymbol{U}_b^{\dagger}\right)^{-1}\left(\boldsymbol{U}^{\sigma\dagger}\right)^{-1}= \tag{S37}$$

$$=-i\left[\boldsymbol{U}^{\sigma\dagger}\boldsymbol{U}_b^{\dagger}\boldsymbol{\mathcal{H}}_{KS}^{\sigma}\boldsymbol{U}_b\boldsymbol{U}^{\sigma},(\boldsymbol{U}^{\sigma})^{-1}\boldsymbol{U}_b^{-1}\boldsymbol{\mathcal{P}}^{\sigma}\left(\boldsymbol{U}_b^{\dagger}\right)^{-1}\left(\boldsymbol{U}^{\sigma\dagger}\right)^{-1}\right]-\Gamma^{\sigma}\begin{pmatrix}\tilde{\tilde{\boldsymbol{\mathcal{P}}}}_L^{\sigma}-{\tilde{\tilde{\boldsymbol{\mathcal{P}}}}_L^{\sigma}}^0 & \frac{1}{2}\tilde{\tilde{\boldsymbol{\mathcal{P}}}}_{L,EM}^{\sigma} & \tilde{\tilde{\boldsymbol{\mathcal{P}}}}_{LR}^{\sigma}\\ \frac{1}{2}\tilde{\tilde{\boldsymbol{\mathcal{P}}}}_{EM,L}^{\sigma} & \mathbf{0} & \frac{1}{2}\tilde{\tilde{\boldsymbol{\mathcal{P}}}}_{EM,R}^{\sigma}\\ \tilde{\tilde{\boldsymbol{\mathcal{P}}}}_{RL}^{\sigma} & \frac{1}{2}\tilde{\tilde{\boldsymbol{\mathcal{P}}}}_{R,EM}^{\sigma} & \tilde{\tilde{\boldsymbol{\mathcal{P}}}}_R^{\sigma}-{\tilde{\tilde{\boldsymbol{\mathcal{P}}}}_R^{\sigma}}^0\end{pmatrix}=$$

$$=-i\left[\boldsymbol{U}^{\sigma\dagger}\boldsymbol{U}_b^{\dagger}\boldsymbol{\mathcal{H}}_{KS}^{\sigma}\boldsymbol{U}_b\boldsymbol{U}^{\sigma}(\boldsymbol{U}^{\sigma})^{-1}\boldsymbol{U}_b^{-1}\boldsymbol{\mathcal{P}}^{\sigma}\left(\boldsymbol{U}_b^{\dagger}\right)^{-1}\left(\boldsymbol{U}^{\sigma\dagger}\right)^{-1}-(\boldsymbol{U}^{\sigma})^{-1}\boldsymbol{U}_b^{-1}\boldsymbol{\mathcal{P}}^{\sigma}\left(\boldsymbol{U}_b^{\dagger}\right)^{-1}\left(\boldsymbol{U}^{\sigma\dagger}\right)^{-1}\boldsymbol{U}^{\sigma\dagger}\boldsymbol{U}_b^{\dagger}\boldsymbol{\mathcal{H}}_{KS}^{\sigma}\boldsymbol{U}_b\boldsymbol{U}^{\sigma}\right]$$

$$-\Gamma^{\sigma}\begin{pmatrix} \widetilde{\widetilde{\boldsymbol{\mathcal{P}}}}_{L}^{\sigma}-\widetilde{\widetilde{\boldsymbol{\mathcal{P}}}}_{L}^{\sigma^{0}} & \frac{1}{2}\widetilde{\widetilde{\boldsymbol{\mathcal{P}}}}_{L,EM}^{\sigma} & \widetilde{\widetilde{\boldsymbol{\mathcal{P}}}}_{LR}^{\sigma} \\ \frac{1}{2}\widetilde{\widetilde{\boldsymbol{\mathcal{P}}}}_{EM,L}^{\sigma} & \mathbf{0} & \frac{1}{2}\widetilde{\widetilde{\boldsymbol{\mathcal{P}}}}_{EM,R}^{\sigma} \\ \widetilde{\widetilde{\boldsymbol{\mathcal{P}}}}_{RL}^{\sigma} & \frac{1}{2}\widetilde{\widetilde{\boldsymbol{\mathcal{P}}}}_{R,EM}^{\sigma} & \widetilde{\widetilde{\boldsymbol{\mathcal{P}}}}_{R}^{\sigma}-\widetilde{\widetilde{\boldsymbol{\mathcal{P}}}}_{R}^{\sigma^{0}} \end{pmatrix}=$$

$$=-i\boldsymbol{U}^{\sigma\dagger}\boldsymbol{U}_{b}^{\dagger}\boldsymbol{\mathcal{H}}_{KS}^{\sigma}\boldsymbol{\mathcal{P}}^{\sigma}\left(\boldsymbol{U}_{b}^{\dagger}\right)^{-1}\left(\boldsymbol{U}^{\sigma\dagger}\right)^{-1}+i(\boldsymbol{U}^{\sigma})^{-1}\boldsymbol{U}_{b}^{-1}\boldsymbol{\mathcal{P}}^{\sigma}\boldsymbol{\mathcal{H}}_{KS}^{\sigma}\boldsymbol{U}_{b}\boldsymbol{U}^{\sigma}-\Gamma^{\sigma}\begin{pmatrix} \widetilde{\widetilde{\boldsymbol{\mathcal{P}}}}_{L}^{\sigma}-\widetilde{\widetilde{\boldsymbol{\mathcal{P}}}}_{L}^{\sigma^{0}} & \frac{1}{2}\widetilde{\widetilde{\boldsymbol{\mathcal{P}}}}_{L,EM}^{\sigma} & \widetilde{\widetilde{\boldsymbol{\mathcal{P}}}}_{LR}^{\sigma} \\ \frac{1}{2}\widetilde{\widetilde{\boldsymbol{\mathcal{P}}}}_{EM,L}^{\sigma} & \mathbf{0} & \frac{1}{2}\widetilde{\widetilde{\boldsymbol{\mathcal{P}}}}_{EM,R}^{\sigma} \\ \widetilde{\widetilde{\boldsymbol{\mathcal{P}}}}_{RL}^{\sigma} & \frac{1}{2}\widetilde{\widetilde{\boldsymbol{\mathcal{P}}}}_{R,EM}^{\sigma} & \widetilde{\widetilde{\boldsymbol{\mathcal{P}}}}_{R}^{\sigma}-\widetilde{\widetilde{\boldsymbol{\mathcal{P}}}}_{R}^{\sigma^{0}} \end{pmatrix}.$$

Multiplying by $\boldsymbol{U}_{b}\boldsymbol{U}^{\sigma}$ on the left and $\boldsymbol{U}^{\sigma\dagger}\boldsymbol{U}_{b}^{\dagger}$ on the right we obtain:

$$\dot{\boldsymbol{\mathcal{P}}}^{\sigma}=-i\boldsymbol{U}_{b}\boldsymbol{U}^{\sigma}\boldsymbol{U}^{\sigma\dagger}\boldsymbol{U}_{b}^{\dagger}\boldsymbol{\mathcal{H}}_{KS}^{\sigma}\boldsymbol{\mathcal{P}}^{\sigma}+i\boldsymbol{\mathcal{P}}^{\sigma}\boldsymbol{\mathcal{H}}_{KS}^{\sigma}\boldsymbol{U}_{b}\boldsymbol{U}^{\sigma}\boldsymbol{U}^{\sigma\dagger}\boldsymbol{U}_{b}^{\dagger}-\Gamma^{\sigma}\boldsymbol{U}_{b}\boldsymbol{U}^{\sigma}\begin{pmatrix} \widetilde{\widetilde{\boldsymbol{\mathcal{P}}}}_{L}^{\sigma}-\widetilde{\widetilde{\boldsymbol{\mathcal{P}}}}_{L}^{\sigma^{0}} & \frac{1}{2}\widetilde{\widetilde{\boldsymbol{\mathcal{P}}}}_{L,EM}^{\sigma} & \widetilde{\widetilde{\boldsymbol{\mathcal{P}}}}_{LR}^{\sigma} \\ \frac{1}{2}\widetilde{\widetilde{\boldsymbol{\mathcal{P}}}}_{EM,L}^{\sigma} & \mathbf{0} & \frac{1}{2}\widetilde{\widetilde{\boldsymbol{\mathcal{P}}}}_{EM,R}^{\sigma} \\ \widetilde{\widetilde{\boldsymbol{\mathcal{P}}}}_{RL}^{\sigma} & \frac{1}{2}\widetilde{\widetilde{\boldsymbol{\mathcal{P}}}}_{R,EM}^{\sigma} & \widetilde{\widetilde{\boldsymbol{\mathcal{P}}}}_{R}^{\sigma}-\widetilde{\widetilde{\boldsymbol{\mathcal{P}}}}_{R}^{\sigma^{0}} \end{pmatrix}\boldsymbol{U}^{\sigma\dagger}\boldsymbol{U}_{b}^{\dagger}. \quad \text{(S38)}$$

Since, by construction, the transformation $\boldsymbol{U}^{\sigma}$ obeys the relation $\boldsymbol{U}^{\sigma\dagger}\widetilde{\boldsymbol{\mathcal{S}}}\boldsymbol{U}^{\sigma}=\boldsymbol{I}$, we may write $\widetilde{\boldsymbol{\mathcal{S}}}=\left(\boldsymbol{U}^{\sigma\dagger}\right)^{-1}\boldsymbol{U}^{\sigma-1}=\left(\boldsymbol{U}^{\sigma}\boldsymbol{U}^{\sigma\dagger}\right)^{-1}$, such that $\boldsymbol{U}^{\sigma}\boldsymbol{U}^{\sigma\dagger}=\widetilde{\boldsymbol{\mathcal{S}}}^{-1}$. Similarly, from Eq. (S19) we have $\widetilde{\boldsymbol{\mathcal{S}}}\equiv\boldsymbol{U}_{b}^{\dagger}\boldsymbol{\mathcal{S}}\boldsymbol{U}_{b}$. We may therefore write $\widetilde{\boldsymbol{\mathcal{S}}}^{-1}=\left(\boldsymbol{U}_{b}^{\dagger}\boldsymbol{\mathcal{S}}\boldsymbol{U}_{b}\right)^{-1}=\boldsymbol{U}_{b}^{-1}\boldsymbol{\mathcal{S}}^{-1}\left(\boldsymbol{U}_{b}^{\dagger}\right)^{-1}$. Solving for $\boldsymbol{\mathcal{S}}^{-1}$ we obtain $\boldsymbol{\mathcal{S}}^{-1}=\boldsymbol{U}_{b}\widetilde{\boldsymbol{\mathcal{S}}}^{-1}\boldsymbol{U}_{b}^{\dagger}$. Hence, we obtain $\boldsymbol{U}_{b}\boldsymbol{U}^{\sigma}\boldsymbol{U}^{\sigma\dagger}\boldsymbol{U}_{b}^{\dagger}=\boldsymbol{U}_{b}\widetilde{\boldsymbol{\mathcal{S}}}^{-1}\boldsymbol{U}_{b}^{\dagger}=\boldsymbol{\mathcal{S}}^{-1}$ such that:

$$\dot{\boldsymbol{\mathcal{P}}}^{\sigma}=-i\boldsymbol{\mathcal{S}}^{-1}\boldsymbol{\mathcal{H}}_{KS}^{\sigma}\boldsymbol{\mathcal{P}}^{\sigma}+i\boldsymbol{\mathcal{P}}^{\sigma}\boldsymbol{\mathcal{H}}_{KS}^{\sigma}\boldsymbol{\mathcal{S}}^{-1}-\Gamma^{\sigma}\boldsymbol{U}_{b}\boldsymbol{U}^{\sigma}\begin{pmatrix} \widetilde{\widetilde{\boldsymbol{\mathcal{P}}}}_{L}^{\sigma}-\widetilde{\widetilde{\boldsymbol{\mathcal{P}}}}_{L}^{\sigma^{0}} & \frac{1}{2}\widetilde{\widetilde{\boldsymbol{\mathcal{P}}}}_{L,EM}^{\sigma} & \widetilde{\widetilde{\boldsymbol{\mathcal{P}}}}_{LR}^{\sigma} \\ \frac{1}{2}\widetilde{\widetilde{\boldsymbol{\mathcal{P}}}}_{EM,L}^{\sigma} & \mathbf{0} & \frac{1}{2}\widetilde{\widetilde{\boldsymbol{\mathcal{P}}}}_{EM,R}^{\sigma} \\ \widetilde{\widetilde{\boldsymbol{\mathcal{P}}}}_{RL}^{\sigma} & \frac{1}{2}\widetilde{\widetilde{\boldsymbol{\mathcal{P}}}}_{R,EM}^{\sigma} & \widetilde{\widetilde{\boldsymbol{\mathcal{P}}}}_{R}^{\sigma}-\widetilde{\widetilde{\boldsymbol{\mathcal{P}}}}_{R}^{\sigma^{0}} \end{pmatrix}\boldsymbol{U}^{\sigma\dagger}\boldsymbol{U}_{b}^{\dagger}. \quad \text{(S39)}$$

This is the collinear spin-uncompensated DLvN EOM—our working equation—given in the site representation, which we use for the time propagation.

In practice, the propagation is performed as follows:

1. Construct a junction model with predefined leads and extended molecule sections.
2. Perform a ground state calculation to obtain the overlap matrix, $\boldsymbol{\mathcal{S}}$, and the initial Kohn-Sham Hamiltonian, $\boldsymbol{\mathcal{H}}_{KS}^{\sigma}$, and density, $\boldsymbol{\mathcal{P}}^{\sigma}$, matrices in the site representation.
3. Build the matrix transformation $\boldsymbol{U}_{b}$ (Eq. (S18)).
4. Transform $\boldsymbol{\mathcal{H}}_{KS}^{\sigma}\rightarrow\widetilde{\boldsymbol{\mathcal{H}}}_{KS}^{\sigma}$ from the site representation to the block diagonal basis (Eq. (S19)).

5. Calculate $\boldsymbol{U}^{\sigma}_{L/EM/R,}$ and $\boldsymbol{\varepsilon}_{L/EM/R}$ by solving the generalized eigenvalue equations for $\widetilde{\boldsymbol{\mathcal{H}}}^{\sigma}_{KS_{L/EM/R}}$ and $\tilde{\boldsymbol{S}}_{L/EM/R}$, and transform $\widetilde{\boldsymbol{\mathcal{H}}}^{\sigma}_{KS} \rightarrow \widetilde{\widetilde{\boldsymbol{\mathcal{H}}}}^{\sigma}_{KS}$ from the block diagonal basis to the state representation (Eq. (S28)).
6. Construct the $\widetilde{\widetilde{\boldsymbol{\mathcal{P}}}}^{\sigma}_{L}{}^{0}$ and $\widetilde{\widetilde{\boldsymbol{\mathcal{P}}}}^{\sigma}_{R}{}^{0}$ blocks using the left and right lead model eigenstates, $\boldsymbol{\varepsilon}_{L/R}$, obtained in step 5 above (see text above Eq. (S35)).
7. Propagate $\boldsymbol{\mathcal{P}}^{\sigma}$ (Eq. (S39)).
8. Construct the new $\boldsymbol{\mathcal{H}}^{\sigma}_{KS}$ from the new $\boldsymbol{\mathcal{P}}^{\sigma}$**.**
9. If the time has not exceeded the maximal time, return to step 4.

2. Driving rate sensitivity test

The driving rate, Γ^{σ}, appearing in Eq. (13) of the main text can, in principle, be determined from the self-energy of the semi-infinite lead models.[2] Nonetheless, in our current implementation it is treated as a tunable parameter, chosen such that the density of states (DOS) of the finite lead model fits that of the corresponding semi-infinite lead. To determine the value to be used in the dynamical simulations, we broaden the discrete energy levels of the finite lead models with Lorentzian functions of different widths and adopt a Lorentzian width parameter that provides a density of states that represents well that of a semi-infinite system (not too narrow to provide a discrete spectrum and not too wide to artificially wash out the electronic structure features of the lead) as our Γ^{σ} value for the time-dependent calculations.[3–5] DOS fitting for H chain leads of different lengths and choices of atomic basis sets are shown in Fig. S1, demonstrating good agreement between the semi-infinite and finite-broadened cases, especially within the Fermi transport region set by the bias voltage and electronic temperatures (see insets).

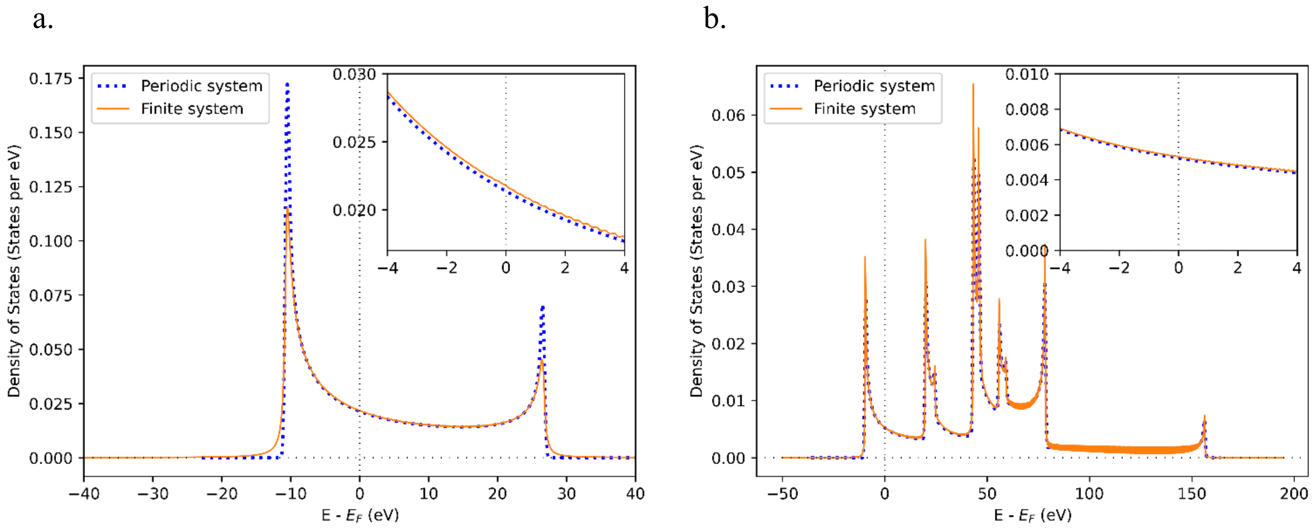

Figure S1: Comparison of the Lorentzian-broadened density of states for the semi-infinite (blue dotted line) and finite (orange solid line) hydrogen chain lead model. Panel (a) shows results obtained for a 180 atom chain at the PBE/STO-3G level of theory with Lorentzian broadening of $\hbar\Gamma^{\sigma} = 0.61\ eV$. Panel (b) shows results obtained for a 300-atom chain at the PBE/6-31G(d, p) level of theory with Lorentzian broadening of $\hbar\Gamma^{\sigma} = 0.2638\ eV$. The DOS of the infinite system was extracted from the band structure obtained from the periodic boundary conditions calculation of the corresponding unit-cell.

The broadened finite lead density of states for the graphitic junction model depicted in Fig. 1(c) of the main text appears in Fig. S2. The adopted broadening of $\hbar\Gamma^{\sigma} = 1.00$ eV, used in the main text, provides satisfactory agreement with the density of states of the corresponding infinite armchair graphene nanoribbon (see Fig. S3).

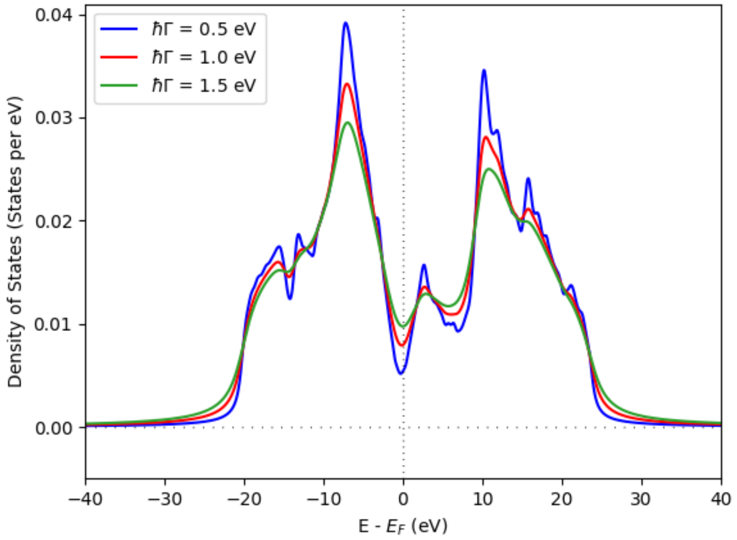

Figure S2: Lorentzian broadened density of states, calculated at the PBE/STO-3G level of theory, around the HOMO-LUMO gap of the hydrogen passivated 176 carbon atoms armchair graphene nanoribbon segment, used as lead model in the zigzag graphene nanoribbon junction (Fig. 1(c) in the main text), for different values of the Lorentzian width, $\hbar\Gamma$.

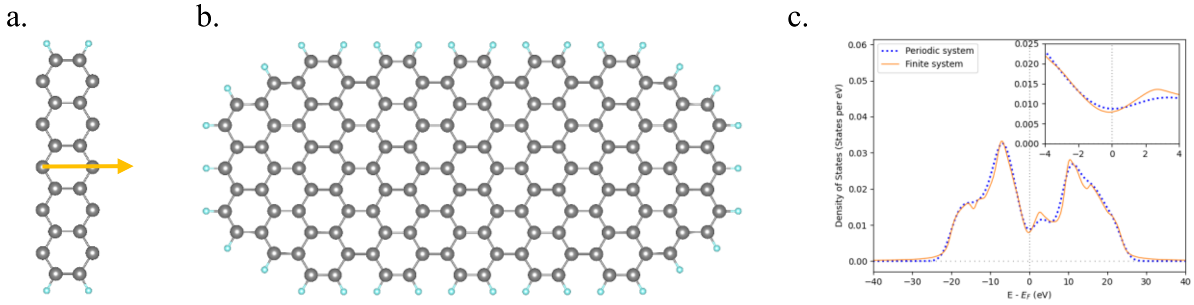

Figure S3: The geometries of the (a) infinite and (b) finite hydrogen passivated armchair GNRs, and (c) the comparison of their Lorentzian-broadened density of states in blue dotted line, and orange solid line, respectively. For the finite lead model calculation a broadening factor of $\hbar\Gamma^{\sigma} = 1.00$ eV was used.

3. Total current calculation

Within the DLvN scheme the construction of the Kohn-Sham Hamiltonian and the propagation are performed in the site (atomic basis) representation (Eq. (S39)), whereas the boundary conditions are applied in the state representation. For calculating the electronic current flowing through the extended molecule section, however, one must transform to the block-diagonal representation. The reason for this is that in the site representation the off-diagonal overlap blocks mix the EM and driven lead section bases, making it unfeasible to isolate the pure EM current contribution. In the state representation, where this problem is remedied, we lack an equation for $\dot{\tilde{\tilde{\boldsymbol{\mathcal{P}}}}}^{\sigma}$, having instead an equation for $\tilde{\tilde{\dot{\boldsymbol{\mathcal{P}}}}}^{\sigma}$. This, in turn, prohibits the actual current calculation. In the block-diagonal basis, these two problems are eliminated as, on the one hand the off-diagonal overlap blocks vanish and on the other hand there is an explicit equation of motion for $\dot{\tilde{\boldsymbol{\mathcal{P}}}}^{\sigma}$, as demonstrated below.

We are interested in calculating the instantaneous particle current flowing between the L and R driven lead sections through the EM region. This can be obtained from the expression for the time derivative of the particle number in this region, $\dot{N}_{EM}^{\sigma}$. To this end, the relation $N^{\sigma}(t) = tr[\boldsymbol{\mathcal{P}}^{\sigma}(t)\boldsymbol{S}]$ can be used, where N^{σ} is the instantaneous number of σ electrons in the explicit junction model considered, $\boldsymbol{S}$ is the atomic orbitals overlap matrix, and $\boldsymbol{\mathcal{P}}^{\sigma}(t)$ is the single-particle density matrix at time t. Note that this expression holds also for the case of fractional occupations encountered in our out-of-equilibrium calculations (see SI section 6 in Oz et. al[6]). Care should be taken, however, when taking the partial trace of this expression to obtain the number of electrons in the EM section, as the density matrix is complex Hermitian. In this case we have,

$$[tr_{EM}(\boldsymbol{\mathcal{P}}^{\sigma}\boldsymbol{S})]^* = [\sum_{\nu\in EM}\sum_{\lambda}\boldsymbol{\mathcal{P}}^{\sigma}{}_{\nu\lambda}\boldsymbol{S}_{\lambda\nu}]^* = \sum_{\nu\in EM}\sum_{\lambda}\boldsymbol{\mathcal{P}}^{\sigma*}{}_{\nu\lambda}\boldsymbol{S}^*_{\lambda\nu} = \sum_{\nu\in EM}\sum_{\lambda}\boldsymbol{\mathcal{P}}^{\sigma}{}_{\lambda\nu}\boldsymbol{S}_{\lambda\nu} =$$
$$= \sum_{\nu\in EM}\sum_{\lambda}\boldsymbol{\mathcal{P}}^{\sigma}{}_{\lambda\nu}\boldsymbol{S}_{\nu\lambda} = \sum_{\nu\in EM}\sum_{\lambda}\boldsymbol{S}_{\nu\lambda}\boldsymbol{\mathcal{P}}^{\sigma}{}_{\lambda\nu} = tr_{EM}(\boldsymbol{S}\boldsymbol{\mathcal{P}}^{\sigma}), \tag{S40}$$

and

$$[tr_{EM}(\boldsymbol{S}\boldsymbol{\mathcal{P}}^{\sigma})]^* = \left[\sum_{\nu\in EM}\sum_{\lambda}\boldsymbol{S}_{\nu\lambda}\boldsymbol{\mathcal{P}}^{\sigma}{}_{\lambda\nu}\right]^* = \sum_{\nu\in EM}\sum_{\lambda}\boldsymbol{S}^*_{\nu\lambda}\boldsymbol{\mathcal{P}}^{*\sigma}_{\lambda\nu} = \sum_{\nu\in EM}\sum_{\lambda}\boldsymbol{S}_{\nu\lambda}\boldsymbol{\mathcal{P}}^{\sigma}_{\nu\lambda} = \sum_{\nu\in EM}\sum_{\lambda}\boldsymbol{S}_{\lambda\nu}\boldsymbol{\mathcal{P}}^{\sigma}_{\nu\lambda} =$$
$$= \sum_{\nu\in EM}\sum_{\lambda}\boldsymbol{\mathcal{P}}^{\sigma}_{\nu\lambda}\boldsymbol{S}_{\lambda\nu} = tr_{EM}(\boldsymbol{\mathcal{P}}^{\sigma}\boldsymbol{S}), \tag{S41}$$

where we used the fact that $\boldsymbol{\mathcal{P}}^{\sigma}$ is Hermitian and $\boldsymbol{S}$ is real and symmetric. We, therefore, see that $tr_{EM}(\boldsymbol{\mathcal{P}}^{\sigma}\boldsymbol{S})$ is not necessarily real and therefore cannot represent the particle number in the EM section. This can be remedied by using the Löwdin symmetric version of the trace formula:

$$N = tr\left(\boldsymbol{S}^{\frac{1}{2}}\boldsymbol{\mathcal{P}}^{\sigma}\boldsymbol{S}^{\frac{1}{2}}\right), \tag{S42}$$

whose partial trace is real:

$$\left[tr_{EM}\left(\boldsymbol{S}^{\frac{1}{2}}\boldsymbol{\mathcal{P}}^{\sigma}\boldsymbol{S}^{\frac{1}{2}}\right)\right]^{*} = \left[\sum_{\mu\in EM}\sum_{\nu}\sum_{\lambda}\boldsymbol{S}^{\frac{1}{2}}_{\mu\nu}\boldsymbol{\mathcal{P}}^{\sigma}_{\nu\lambda}\boldsymbol{S}^{\frac{1}{2}}_{\lambda\mu}\right]^{*} = \sum_{\mu\in EM}\sum_{\nu}\sum_{\lambda}\boldsymbol{S}^{\frac{1}{2}\,*}_{\mu\nu}\,\boldsymbol{\mathcal{P}}^{*\sigma}_{\nu\lambda}\boldsymbol{S}^{\frac{1}{2}\,*}_{\lambda\mu} =$$

$$= \sum_{\mu\in EM}\sum_{\nu}\sum_{\lambda}\boldsymbol{S}^{\frac{1}{2}}_{\mu\nu}\boldsymbol{\mathcal{P}}^{\sigma}_{\lambda\nu}\boldsymbol{S}^{\frac{1}{2}}_{\lambda\mu} = \sum_{\mu\in EM}\sum_{\nu}\sum_{\lambda}\boldsymbol{S}^{\frac{1}{2}}_{\lambda\mu}\boldsymbol{\mathcal{P}}^{\sigma}_{\lambda\nu}\boldsymbol{S}^{\frac{1}{2}}_{\mu\nu} = tr_{EM}\left(\boldsymbol{S}^{\frac{1}{2}}\boldsymbol{\mathcal{P}}^{\sigma}\boldsymbol{S}^{\frac{1}{2}}\right). \tag{S43}$$

The full trace obeys the cyclic property so we can write:

$$N = tr\left(\boldsymbol{S}^{\frac{1}{2}}\boldsymbol{\mathcal{P}}^{\sigma}\boldsymbol{S}^{\frac{1}{2}}\right) = tr(\boldsymbol{\mathcal{P}}^{\sigma}\boldsymbol{S}) = tr(\boldsymbol{S}\boldsymbol{\mathcal{P}}^{\sigma}) = \frac{1}{2}[tr(\boldsymbol{\mathcal{P}}^{\sigma}\boldsymbol{S}) + tr(\boldsymbol{S}\boldsymbol{\mathcal{P}}^{\sigma})]. \tag{S44}$$

The reason for introducing the last term in Eq. (S44) is that per Eqs. (S40) and (S41) its partial trace over the EM section is real-valued. This expression can be transformed to the block diagonal representation using the transformations of Eqs. (S18) and (S19) in SI section 1:

$$N^{\sigma}(t) = \frac{1}{2}[tr(\boldsymbol{\mathcal{P}}^{\sigma}\boldsymbol{S}) + tr(\boldsymbol{S}\boldsymbol{\mathcal{P}}^{\sigma})] =$$

$$= \frac{1}{2}\left[tr\left(\boldsymbol{U}_b\tilde{\boldsymbol{\mathcal{P}}}^{\sigma}(t)\boldsymbol{U}_b^{\dagger}\left(\boldsymbol{U}_b^{\dagger}\right)^{-1}\tilde{\boldsymbol{S}}\boldsymbol{U}_b^{-1}\right) + tr\left(\left(\boldsymbol{U}_b^{\dagger}\right)^{-1}\tilde{\boldsymbol{S}}\boldsymbol{U}_b^{-1}\boldsymbol{U}_b\tilde{\boldsymbol{\mathcal{P}}}^{\sigma}(t)\boldsymbol{U}_b^{\dagger}\right)\right] = \tag{S45}$$

$$= \frac{1}{2}\left[tr\left(\boldsymbol{U}_b\tilde{\boldsymbol{\mathcal{P}}}^{\sigma}(t)\tilde{\boldsymbol{S}}\boldsymbol{U}_b^{-1}\right) + tr\left(\left(\boldsymbol{U}_b^{\dagger}\right)^{-1}\tilde{\boldsymbol{S}}\tilde{\boldsymbol{\mathcal{P}}}^{\sigma}(t)\boldsymbol{U}_b^{\dagger}\right)\right] = \frac{1}{2}\left[tr\left(\tilde{\boldsymbol{\mathcal{P}}}^{\sigma}(t)\tilde{\boldsymbol{S}}\boldsymbol{U}_b^{-1}\boldsymbol{U}_b\right) +\right.$$

$$\left. tr\left(\tilde{\boldsymbol{S}}\tilde{\boldsymbol{\mathcal{P}}}^{\sigma}(t)\boldsymbol{U}_b^{\dagger}\left(\boldsymbol{U}_b^{\dagger}\right)^{-1}\right)\right] =$$

$$= \frac{1}{2}\left[tr\left(\tilde{\boldsymbol{\mathcal{P}}}^{\sigma}(t)\tilde{\boldsymbol{S}}\right) + tr\left(\tilde{\boldsymbol{S}}\tilde{\boldsymbol{\mathcal{P}}}^{\sigma}(t)\right)\right].$$

Since the block diagonalization transformation only rotates the EM basis to make it diagonal to the L and R bases without modifying the latter (while readjusting the L/EM and R/EM Kohn-Sham Hamiltonian coupling blocks), the partial sums over the L, EM, and R indices retain their spatial interpretation as belonging to the corresponding system sections. We can, therefore, write the full trace as the sum of partial traces over the separate system sections:

$$N^{\sigma}(t) = \frac{1}{2}\left[tr\left(\tilde{\boldsymbol{\mathcal{P}}}^{\sigma}(t)\tilde{\boldsymbol{S}}\right) + tr\left(\tilde{\boldsymbol{S}}\tilde{\boldsymbol{\mathcal{P}}}^{\sigma}(t)\right)\right] = \tag{S46}$$

$$= \frac{1}{2}\left\{\left[tr_L\left(\tilde{\boldsymbol{\mathcal{P}}}^{\sigma}(t)\tilde{\boldsymbol{S}}\right) + tr_L\left(\tilde{\boldsymbol{S}}\tilde{\boldsymbol{\mathcal{P}}}^{\sigma}(t)\right)\right] + \left[tr_{EM}\left(\tilde{\boldsymbol{\mathcal{P}}}^{\sigma}(t)\tilde{\boldsymbol{S}}\right) + tr_{EM}\left(\tilde{\boldsymbol{S}}\tilde{\boldsymbol{\mathcal{P}}}^{\sigma}(t)\right)\right] + \left[tr_R\left(\tilde{\boldsymbol{\mathcal{P}}}^{\sigma}(t)\tilde{\boldsymbol{S}}\right) + tr_R\left(\tilde{\boldsymbol{S}}\tilde{\boldsymbol{\mathcal{P}}}^{\sigma}(t)\right)\right]\right\},$$

where we identify:

$$N^{\sigma}{}_{\alpha=L,EM,R}(t) = \frac{1}{2}\left[tr_{\alpha}\left(\tilde{\boldsymbol{\mathcal{P}}}^{\sigma}(t)\tilde{\boldsymbol{S}}\right) + tr_{\alpha}\left(\tilde{\boldsymbol{S}}\tilde{\boldsymbol{\mathcal{P}}}^{\sigma}(t)\right)\right] = \frac{1}{2}tr_{\alpha}\left[\tilde{\boldsymbol{\mathcal{P}}}^{\sigma}(t)\tilde{\boldsymbol{S}} + \tilde{\boldsymbol{S}}\tilde{\boldsymbol{\mathcal{P}}}^{\sigma}(t)\right] \tag{S47}$$

as the instantaneous number of electrons in the different sections.

Since $\tilde{\boldsymbol{S}}$ is time independent (for fixed nuclei positions) we may express the temporal change in the

number of particles in the EM section as:

$$\dot{N}^{\sigma}{}_{EM} = \frac{1}{2} tr_{EM}\left(\dot{\widetilde{\boldsymbol{\mathcal{P}}}}^{\sigma}\widetilde{\boldsymbol{\mathcal{S}}} + \widetilde{\boldsymbol{\mathcal{S}}}\dot{\widetilde{\boldsymbol{\mathcal{P}}}}^{\sigma}\right). \tag{S48}$$

As explained above, to obtain an expression for the average total current flowing through the EM section we now need to write the DLvN EOM for $\widetilde{\boldsymbol{\mathcal{P}}}^{\sigma}$ in the block diagonal basis. This can be achieved by transforming the DLvN EOM from the state representation, where the boundary conditions are readily applied, to the block diagonal basis. The DLvN EOM in the state representation is given by:

$$\dot{\widetilde{\widetilde{\boldsymbol{\mathcal{P}}}}}^{\sigma} = -i\left[\widetilde{\widetilde{\boldsymbol{\mathcal{H}}}}^{\sigma}_{KS}, \widetilde{\widetilde{\boldsymbol{\mathcal{P}}}}^{\sigma}\right] - \Gamma^{\sigma}\begin{pmatrix} \widetilde{\widetilde{\boldsymbol{\mathcal{P}}}}^{\sigma}_{L} - \widetilde{\widetilde{\boldsymbol{\mathcal{P}}}}^{0}_{L}{}^{\sigma} & \frac{1}{2}\widetilde{\widetilde{\boldsymbol{\mathcal{P}}}}^{\sigma}_{L,EM} & \widetilde{\widetilde{\boldsymbol{\mathcal{P}}}}^{\sigma}_{LR} \\ \frac{1}{2}\widetilde{\widetilde{\boldsymbol{\mathcal{P}}}}^{\sigma}_{EM,L} & \mathbf{0} & \frac{1}{2}\widetilde{\widetilde{\boldsymbol{\mathcal{P}}}}^{\sigma}_{EM,R} \\ \widetilde{\widetilde{\boldsymbol{\mathcal{P}}}}^{\sigma}_{RL} & \frac{1}{2}\widetilde{\widetilde{\boldsymbol{\mathcal{P}}}}^{\sigma}_{R,EM} & \widetilde{\widetilde{\boldsymbol{\mathcal{P}}}}^{\sigma}_{R} - \widetilde{\widetilde{\boldsymbol{\mathcal{P}}}}^{0}_{R}{}^{\sigma} \end{pmatrix}. \tag{S49}$$

Using the back transformation from the state- to the block diagonal representation (the inverse transformation of Eq. (S28) of SI section 1), and the fact that $\boldsymbol{U}_b$ is time-independent (see Eq. (S18) of SI section 1) so that $\dot{\widetilde{\boldsymbol{\mathcal{P}}}}^{\sigma} = \frac{d}{dt}\left[\boldsymbol{U}_b^{-1}\boldsymbol{\mathcal{P}}^{\sigma}\left(\boldsymbol{U}_b^{\dagger}\right)^{-1}\right] = \boldsymbol{U}_b^{-1}\dot{\boldsymbol{\mathcal{P}}}^{\sigma}\left(\boldsymbol{U}_b^{\dagger}\right)^{-1} = \widetilde{\dot{\boldsymbol{\mathcal{P}}}}^{\sigma}$, we may write:

$$\dot{\widetilde{\boldsymbol{\mathcal{P}}}}^{\sigma} = \widetilde{\dot{\boldsymbol{\mathcal{P}}}}^{\sigma} = \boldsymbol{U}^{\sigma}\dot{\widetilde{\widetilde{\boldsymbol{\mathcal{P}}}}}^{\sigma}\boldsymbol{U}^{\sigma\dagger} = -i\boldsymbol{U}^{\sigma}\left[\widetilde{\widetilde{\boldsymbol{\mathcal{H}}}}^{\sigma}_{KS}, \widetilde{\widetilde{\boldsymbol{\mathcal{P}}}}^{\sigma}\right]\boldsymbol{U}^{\sigma\dagger} - \Gamma^{\sigma}\boldsymbol{U}^{\sigma}\begin{pmatrix} \widetilde{\widetilde{\boldsymbol{\mathcal{P}}}}^{\sigma}_{L} - \widetilde{\widetilde{\boldsymbol{\mathcal{P}}}}^{0}_{L}{}^{\sigma} & \frac{1}{2}\widetilde{\widetilde{\boldsymbol{\mathcal{P}}}}^{\sigma}_{L,EM} & \widetilde{\widetilde{\boldsymbol{\mathcal{P}}}}^{\sigma}_{LR} \\ \frac{1}{2}\widetilde{\widetilde{\boldsymbol{\mathcal{P}}}}^{\sigma}_{EM,L} & \mathbf{0} & \frac{1}{2}\widetilde{\widetilde{\boldsymbol{\mathcal{P}}}}^{\sigma}_{EM,R} \\ \widetilde{\widetilde{\boldsymbol{\mathcal{P}}}}^{\sigma}_{RL} & \frac{1}{2}\widetilde{\widetilde{\boldsymbol{\mathcal{P}}}}^{\sigma}_{R,EM} & \widetilde{\widetilde{\boldsymbol{\mathcal{P}}}}^{\sigma}_{R} - \widetilde{\widetilde{\boldsymbol{\mathcal{P}}}}^{0}_{R}{}^{\sigma} \end{pmatrix}\boldsymbol{U}^{\sigma\dagger}. \tag{S50}$$

We shall first transform the driving term on the right-hand side from the state- to the block diagonal representation. To this end, we rewrite it in the following form:

$$-\Gamma^{\sigma}\boldsymbol{U}^{\sigma}\begin{pmatrix} \widetilde{\widetilde{\boldsymbol{\mathcal{P}}}}^{\sigma}_{L} - \widetilde{\widetilde{\boldsymbol{\mathcal{P}}}}^{0}_{L}{}^{\sigma} & \frac{1}{2}\widetilde{\widetilde{\boldsymbol{\mathcal{P}}}}^{\sigma}_{L,EM} & \widetilde{\widetilde{\boldsymbol{\mathcal{P}}}}^{\sigma}_{LR} \\ \frac{1}{2}\widetilde{\widetilde{\boldsymbol{\mathcal{P}}}}^{\sigma}_{EM,L} & \mathbf{0} & \frac{1}{2}\widetilde{\widetilde{\boldsymbol{\mathcal{P}}}}^{\sigma}_{EM,R} \\ \widetilde{\widetilde{\boldsymbol{\mathcal{P}}}}^{\sigma}_{RL} & \frac{1}{2}\widetilde{\widetilde{\boldsymbol{\mathcal{P}}}}^{\sigma}_{R,EM} & \widetilde{\widetilde{\boldsymbol{\mathcal{P}}}}^{\sigma}_{R} - \widetilde{\widetilde{\boldsymbol{\mathcal{P}}}}^{0}_{R}{}^{\sigma} \end{pmatrix}\boldsymbol{U}^{\sigma\dagger} = -\frac{\Gamma^{\sigma}}{2}\boldsymbol{U}^{\sigma}\begin{pmatrix} \boldsymbol{I}_L & \mathbf{0} & \mathbf{0} \\ \mathbf{0} & \mathbf{0} & \mathbf{0} \\ \mathbf{0} & \mathbf{0} & \boldsymbol{I}_R \end{pmatrix}\widetilde{\widetilde{\boldsymbol{\mathcal{P}}}}^{\sigma}\boldsymbol{U}^{\sigma\dagger} -$$

$$-\frac{\Gamma^{\sigma}}{2}\boldsymbol{U}^{\sigma}\widetilde{\widetilde{\boldsymbol{\mathcal{P}}}}^{\sigma}\begin{pmatrix} \boldsymbol{I}_L & \mathbf{0} & \mathbf{0} \\ \mathbf{0} & \mathbf{0} & \mathbf{0} \\ \mathbf{0} & \mathbf{0} & \boldsymbol{I}_R \end{pmatrix}\boldsymbol{U}^{\sigma\dagger} + \frac{\Gamma^{\sigma}}{2}\boldsymbol{U}^{\sigma}\begin{pmatrix} \boldsymbol{I}_L & \mathbf{0} & \mathbf{0} \\ \mathbf{0} & \mathbf{0} & \mathbf{0} \\ \mathbf{0} & \mathbf{0} & \boldsymbol{I}_R \end{pmatrix}\begin{pmatrix} \widetilde{\widetilde{\boldsymbol{\mathcal{P}}}}^{0}_{L}{}^{\sigma} & \mathbf{0} & \mathbf{0} \\ \mathbf{0} & \widetilde{\widetilde{\boldsymbol{\mathcal{P}}}}^{0}_{EM}{}^{\sigma} & \mathbf{0} \\ \mathbf{0} & \mathbf{0} & \widetilde{\widetilde{\boldsymbol{\mathcal{P}}}}^{0}_{R}{}^{\sigma} \end{pmatrix}\boldsymbol{U}^{\sigma\dagger} +$$

$$+\frac{\Gamma^{\sigma}}{2}\boldsymbol{U}^{\sigma}\begin{pmatrix} \widetilde{\widetilde{\boldsymbol{\mathcal{P}}}}^{0}_{L}{}^{\sigma} & \mathbf{0} & \mathbf{0} \\ \mathbf{0} & \widetilde{\widetilde{\boldsymbol{\mathcal{P}}}}^{0}_{EM}{}^{\sigma} & \mathbf{0} \\ \mathbf{0} & \mathbf{0} & \widetilde{\widetilde{\boldsymbol{\mathcal{P}}}}^{0}_{R}{}^{\sigma} \end{pmatrix}\begin{pmatrix} \boldsymbol{I}_L & \mathbf{0} & \mathbf{0} \\ \mathbf{0} & \mathbf{0} & \mathbf{0} \\ \mathbf{0} & \mathbf{0} & \boldsymbol{I}_R \end{pmatrix}\boldsymbol{U}^{\sigma\dagger}. \tag{S51}$$

The first term on the right-hand-side in Eq. (S51) reads:

$$\boldsymbol{U}^{\sigma}\begin{pmatrix}\boldsymbol{I}_L & \mathbf{0} & \mathbf{0}\\ \mathbf{0} & \mathbf{0} & \mathbf{0}\\ \mathbf{0} & \mathbf{0} & \boldsymbol{I}_R\end{pmatrix}\widetilde{\widetilde{\boldsymbol{\mathcal{P}}}}^{\sigma}\boldsymbol{U}^{\sigma\dagger} = \boldsymbol{U}^{\sigma}\begin{pmatrix}\boldsymbol{I}_L & \mathbf{0} & \mathbf{0}\\ \mathbf{0} & \mathbf{0} & \mathbf{0}\\ \mathbf{0} & \mathbf{0} & \boldsymbol{I}_R\end{pmatrix}(\boldsymbol{U}^{\sigma})^{-\mathbf{1}}\boldsymbol{U}^{\sigma}\widetilde{\widetilde{\boldsymbol{\mathcal{P}}}}^{\sigma}\boldsymbol{U}^{\sigma\dagger} =$$

$$= \begin{pmatrix}\boldsymbol{U}_{\boldsymbol{L}}^{\sigma} & \mathbf{0} & \mathbf{0}\\ \mathbf{0} & \boldsymbol{U}_{\boldsymbol{EM}}^{\sigma} & \mathbf{0}\\ \mathbf{0} & \mathbf{0} & \boldsymbol{U}_{\boldsymbol{R}}^{\sigma}\end{pmatrix}\begin{pmatrix}\boldsymbol{I}_L & \mathbf{0} & \mathbf{0}\\ \mathbf{0} & \mathbf{0} & \mathbf{0}\\ \mathbf{0} & \mathbf{0} & \boldsymbol{I}_R\end{pmatrix}\begin{pmatrix}(\boldsymbol{U}_{\boldsymbol{L}}^{\sigma})^{-1} & \mathbf{0} & \mathbf{0}\\ \mathbf{0} & (\boldsymbol{U}_{\boldsymbol{EM}}^{\sigma})^{-1} & \mathbf{0}\\ \mathbf{0} & \mathbf{0} & (\boldsymbol{U}_{\boldsymbol{R}}^{\sigma})^{-1}\end{pmatrix}\widetilde{\boldsymbol{\mathcal{P}}}^{\sigma} = \tag{S52}$$

$$= \begin{pmatrix}\boldsymbol{U}_{\boldsymbol{L}}^{\sigma} & \mathbf{0} & \mathbf{0}\\ \mathbf{0} & \boldsymbol{U}_{\boldsymbol{EM}}^{\sigma} & \mathbf{0}\\ \mathbf{0} & \mathbf{0} & \boldsymbol{U}_{\boldsymbol{R}}^{\sigma}\end{pmatrix}\begin{pmatrix}(\boldsymbol{U}_{\boldsymbol{L}}^{\sigma})^{-1} & \mathbf{0} & \mathbf{0}\\ \mathbf{0} & \mathbf{0} & \mathbf{0}\\ \mathbf{0} & \mathbf{0} & (\boldsymbol{U}_{\boldsymbol{R}}^{\sigma})^{-1}\end{pmatrix}\widetilde{\boldsymbol{\mathcal{P}}}^{\sigma} = \begin{pmatrix}\boldsymbol{I}_L & \mathbf{0} & \mathbf{0}\\ \mathbf{0} & \mathbf{0} & \mathbf{0}\\ \mathbf{0} & \mathbf{0} & \boldsymbol{I}_R\end{pmatrix}\widetilde{\boldsymbol{\mathcal{P}}}^{\sigma} =$$

$$= \begin{pmatrix}\boldsymbol{I}_L & \mathbf{0} & \mathbf{0}\\ \mathbf{0} & \mathbf{0} & \mathbf{0}\\ \mathbf{0} & \mathbf{0} & \boldsymbol{I}_R\end{pmatrix}\begin{pmatrix}\widetilde{\boldsymbol{\mathcal{P}}}_L^{\sigma} & \widetilde{\boldsymbol{\mathcal{P}}}_{L,EM}^{\sigma} & \widetilde{\boldsymbol{\mathcal{P}}}_{L,R}^{\sigma}\\ \widetilde{\boldsymbol{\mathcal{P}}}_{EM,L}^{\sigma} & \widetilde{\boldsymbol{\mathcal{P}}}_{EM}^{\sigma} & \widetilde{\boldsymbol{\mathcal{P}}}_{EM,R}^{\sigma}\\ \widetilde{\boldsymbol{\mathcal{P}}}_{R,L}^{\sigma} & \widetilde{\boldsymbol{\mathcal{P}}}_{R,EM}^{\sigma} & \widetilde{\boldsymbol{\mathcal{P}}}_R^{\sigma}\end{pmatrix} = \begin{pmatrix}\widetilde{\boldsymbol{\mathcal{P}}}_L^{\sigma} & \widetilde{\boldsymbol{\mathcal{P}}}_{L,EM}^{\sigma} & \widetilde{\boldsymbol{\mathcal{P}}}_{L,R}^{\sigma}\\ \mathbf{0} & \mathbf{0} & \mathbf{0}\\ \widetilde{\boldsymbol{\mathcal{P}}}_{R,L}^{\sigma} & \widetilde{\boldsymbol{\mathcal{P}}}_{R,EM}^{\sigma} & \widetilde{\boldsymbol{\mathcal{P}}}_R^{\sigma}\end{pmatrix}.$$

Similarly, the second term on the right-hand-side reads:

$$\boldsymbol{U}^{\sigma}\widetilde{\widetilde{\boldsymbol{\mathcal{P}}}}^{\sigma}\begin{pmatrix}\boldsymbol{I}_L & \mathbf{0} & \mathbf{0}\\ \mathbf{0} & \mathbf{0} & \mathbf{0}\\ \mathbf{0} & \mathbf{0} & \boldsymbol{I}_R\end{pmatrix}\boldsymbol{U}^{\sigma\dagger} = \boldsymbol{U}^{\sigma}\widetilde{\widetilde{\boldsymbol{\mathcal{P}}}}^{\sigma}\boldsymbol{U}^{\sigma\dagger}\left(\boldsymbol{U}^{\sigma\dagger}\right)^{-1}\begin{pmatrix}\boldsymbol{I}_L & \mathbf{0} & \mathbf{0}\\ \mathbf{0} & \mathbf{0} & \mathbf{0}\\ \mathbf{0} & \mathbf{0} & \boldsymbol{I}_R\end{pmatrix}\boldsymbol{U}^{\sigma\dagger} =$$

$$= \widetilde{\boldsymbol{\mathcal{P}}}^{\sigma}\begin{pmatrix}\left(\boldsymbol{U}^{\sigma\dagger}_{L}\right)^{-1} & \mathbf{0} & \mathbf{0}\\ \mathbf{0} & \left(\boldsymbol{U}^{\sigma\dagger}_{EM}\right)^{-1} & \mathbf{0}\\ \mathbf{0} & \mathbf{0} & \left(\boldsymbol{U}^{\sigma\dagger}_{R}\right)^{-1}\end{pmatrix}\begin{pmatrix}\boldsymbol{I}_L & \mathbf{0} & \mathbf{0}\\ \mathbf{0} & \mathbf{0} & \mathbf{0}\\ \mathbf{0} & \mathbf{0} & \boldsymbol{I}_R\end{pmatrix}\begin{pmatrix}\boldsymbol{U}^{\sigma\dagger}_{L} & \mathbf{0} & \mathbf{0}\\ \mathbf{0} & \boldsymbol{U}^{\sigma\dagger}_{EM} & \mathbf{0}\\ \mathbf{0} & \mathbf{0} & \boldsymbol{U}^{\sigma\dagger}_{R}\end{pmatrix} = \tag{S53}$$

$$= \widetilde{\boldsymbol{\mathcal{P}}}^{\sigma}\begin{pmatrix}\left(\boldsymbol{U}^{\sigma\dagger}_{L}\right)^{-1} & \mathbf{0} & \mathbf{0}\\ \mathbf{0} & \left(\boldsymbol{U}^{\sigma\dagger}_{EM}\right)^{-1} & \mathbf{0}\\ \mathbf{0} & \mathbf{0} & \left(\boldsymbol{U}^{\sigma\dagger}_{R}\right)^{-1}\end{pmatrix}\begin{pmatrix}\boldsymbol{U}^{\sigma\dagger}_{L} & \mathbf{0} & \mathbf{0}\\ \mathbf{0} & \mathbf{0} & \mathbf{0}\\ \mathbf{0} & \mathbf{0} & \boldsymbol{U}^{\sigma\dagger}_{R}\end{pmatrix} = \widetilde{\boldsymbol{\mathcal{P}}}^{\sigma}\begin{pmatrix}\boldsymbol{I}_L & \mathbf{0} & \mathbf{0}\\ \mathbf{0} & \mathbf{0} & \mathbf{0}\\ \mathbf{0} & \mathbf{0} & \boldsymbol{I}_R\end{pmatrix} =$$

$$= \begin{pmatrix}\widetilde{\boldsymbol{\mathcal{P}}}_L^{\sigma} & \widetilde{\boldsymbol{\mathcal{P}}}_{L,EM}^{\sigma} & \widetilde{\boldsymbol{\mathcal{P}}}_{L,R}^{\sigma}\\ \widetilde{\boldsymbol{\mathcal{P}}}_{EM,L}^{\sigma} & \widetilde{\boldsymbol{\mathcal{P}}}_{EM}^{\sigma} & \widetilde{\boldsymbol{\mathcal{P}}}_{EM,R}^{\sigma}\\ \widetilde{\boldsymbol{\mathcal{P}}}_{R,L}^{\sigma} & \widetilde{\boldsymbol{\mathcal{P}}}_{R,EM}^{\sigma} & \widetilde{\boldsymbol{\mathcal{P}}}_R^{\sigma}\end{pmatrix}\begin{pmatrix}\boldsymbol{I}_L & \mathbf{0} & \mathbf{0}\\ \mathbf{0} & \mathbf{0} & \mathbf{0}\\ \mathbf{0} & \mathbf{0} & \boldsymbol{I}_R\end{pmatrix} = \begin{pmatrix}\widetilde{\boldsymbol{\mathcal{P}}}_L^{\sigma} & \mathbf{0} & \widetilde{\boldsymbol{\mathcal{P}}}_{L,R}^{\sigma}\\ \widetilde{\boldsymbol{\mathcal{P}}}_{EM,L}^{\sigma} & \mathbf{0} & \widetilde{\boldsymbol{\mathcal{P}}}_{EM,R}^{\sigma}\\ \widetilde{\boldsymbol{\mathcal{P}}}_{R,L}^{\sigma} & \mathbf{0} & \widetilde{\boldsymbol{\mathcal{P}}}_R^{\sigma}\end{pmatrix}.$$

The third term gives:

$$\boldsymbol{U}^{\sigma}\begin{pmatrix}\boldsymbol{I}_L & \mathbf{0} & \mathbf{0}\\ \mathbf{0} & \mathbf{0} & \mathbf{0}\\ \mathbf{0} & \mathbf{0} & \boldsymbol{I}_R\end{pmatrix}\begin{pmatrix}\widetilde{\widetilde{\boldsymbol{\mathcal{P}}}}{}_L^{0}{}^{\sigma} & \mathbf{0} & \mathbf{0}\\ \mathbf{0} & \widetilde{\widetilde{\boldsymbol{\mathcal{P}}}}{}_{EM}^{0}{}^{\sigma} & \mathbf{0}\\ \mathbf{0} & \mathbf{0} & \widetilde{\widetilde{\boldsymbol{\mathcal{P}}}}{}_R^{0}{}^{\sigma}\end{pmatrix}\boldsymbol{U}^{\sigma\dagger} =$$

$$= \begin{pmatrix}\boldsymbol{U}_{\boldsymbol{L}}^{\sigma} & \mathbf{0} & \mathbf{0}\\ \mathbf{0} & \boldsymbol{U}_{\boldsymbol{EM}}^{\sigma} & \mathbf{0}\\ \mathbf{0} & \mathbf{0} & \boldsymbol{U}_{\boldsymbol{R}}^{\sigma}\end{pmatrix}\begin{pmatrix}\widetilde{\widetilde{\boldsymbol{\mathcal{P}}}}{}_L^{0}{}^{\sigma} & \mathbf{0} & \mathbf{0}\\ \mathbf{0} & \mathbf{0} & \mathbf{0}\\ \mathbf{0} & \mathbf{0} & \widetilde{\widetilde{\boldsymbol{\mathcal{P}}}}{}_R^{0}{}^{\sigma}\end{pmatrix}\begin{pmatrix}\boldsymbol{U}^{\sigma\dagger}_{L} & \mathbf{0} & \mathbf{0}\\ \mathbf{0} & \boldsymbol{U}^{\sigma\dagger}_{EM} & \mathbf{0}\\ \mathbf{0} & \mathbf{0} & \boldsymbol{U}^{\sigma\dagger}_{R}\end{pmatrix} =$$

$$
=\begin{pmatrix} \boldsymbol{U}_{\boldsymbol{L}}^{\sigma} & \boldsymbol{0} & \boldsymbol{0} \\ \boldsymbol{0} & \boldsymbol{U}_{\boldsymbol{EM}}^{\sigma} & \boldsymbol{0} \\ \boldsymbol{0} & \boldsymbol{0} & \boldsymbol{U}_{\boldsymbol{R}}^{\sigma} \end{pmatrix}\begin{pmatrix} \tilde{\tilde{\boldsymbol{\mathcal{P}}}}{}_{L}^{0\,\sigma}\boldsymbol{U}^{\sigma\dagger}_{L} & \boldsymbol{0} & \boldsymbol{0} \\ \boldsymbol{0} & \boldsymbol{0} & \boldsymbol{0} \\ \boldsymbol{0} & \boldsymbol{0} & \tilde{\tilde{\boldsymbol{\mathcal{P}}}}{}_{R}^{0\,\sigma}\boldsymbol{U}^{\sigma\dagger}_{R} \end{pmatrix}=\begin{pmatrix} \boldsymbol{U}_{\boldsymbol{L}}^{\sigma}\tilde{\tilde{\boldsymbol{\mathcal{P}}}}{}_{L}^{0\,\sigma}\boldsymbol{U}^{\sigma\dagger}_{L} & \boldsymbol{0} & \boldsymbol{0} \\ \boldsymbol{0} & \boldsymbol{0} & \boldsymbol{0} \\ \boldsymbol{0} & \boldsymbol{0} & \boldsymbol{U}_{\boldsymbol{R}}^{\sigma}\tilde{\tilde{\boldsymbol{\mathcal{P}}}}{}_{R}^{0\,\sigma}\boldsymbol{U}^{\sigma\dagger}_{R} \end{pmatrix}\equiv\begin{pmatrix} \tilde{\boldsymbol{\mathcal{P}}}{}_{L}^{0\,\sigma} & \boldsymbol{0} & \boldsymbol{0} \\ \boldsymbol{0} & \boldsymbol{0} & \boldsymbol{0} \\ \boldsymbol{0} & \boldsymbol{0} & \tilde{\boldsymbol{\mathcal{P}}}{}_{R}^{0\,\sigma} \end{pmatrix}, \tag{S54}
$$

and the fourth term gives:

$$
\boldsymbol{U}^{\sigma}\begin{pmatrix} \tilde{\tilde{\boldsymbol{\mathcal{P}}}}{}_{L}^{0\,\sigma} & \boldsymbol{0} & \boldsymbol{0} \\ \boldsymbol{0} & \tilde{\tilde{\boldsymbol{\mathcal{P}}}}{}_{EM}^{0\,\sigma} & \boldsymbol{0} \\ \boldsymbol{0} & \boldsymbol{0} & \tilde{\tilde{\boldsymbol{\mathcal{P}}}}{}_{R}^{0\,\sigma} \end{pmatrix}\begin{pmatrix} \boldsymbol{I}_{L} & \boldsymbol{0} & \boldsymbol{0} \\ \boldsymbol{0} & \boldsymbol{0} & \boldsymbol{0} \\ \boldsymbol{0} & \boldsymbol{0} & \boldsymbol{I}_{R} \end{pmatrix}\boldsymbol{U}^{\sigma\dagger}=
$$

$$
=\begin{pmatrix} \boldsymbol{U}_{\boldsymbol{L}}^{\sigma} & \boldsymbol{0} & \boldsymbol{0} \\ \boldsymbol{0} & \boldsymbol{U}_{\boldsymbol{EM}}^{\sigma} & \boldsymbol{0} \\ \boldsymbol{0} & \boldsymbol{0} & \boldsymbol{U}_{\boldsymbol{R}}^{\sigma} \end{pmatrix}\begin{pmatrix} \tilde{\tilde{\boldsymbol{\mathcal{P}}}}{}_{L}^{0\,\sigma} & \boldsymbol{0} & \boldsymbol{0} \\ \boldsymbol{0} & \boldsymbol{0} & \boldsymbol{0} \\ \boldsymbol{0} & \boldsymbol{0} & \tilde{\tilde{\boldsymbol{\mathcal{P}}}}{}_{R}^{0\,\sigma} \end{pmatrix}\begin{pmatrix} \boldsymbol{U}^{\sigma\dagger}_{L} & \boldsymbol{0} & \boldsymbol{0} \\ \boldsymbol{0} & \boldsymbol{U}^{\sigma\dagger}_{EM} & \boldsymbol{0} \\ \boldsymbol{0} & \boldsymbol{0} & \boldsymbol{U}^{\sigma\dagger}_{R} \end{pmatrix}=
$$

$$
=\begin{pmatrix} \boldsymbol{U}_{\boldsymbol{L}}^{\sigma} & \boldsymbol{0} & \boldsymbol{0} \\ \boldsymbol{0} & \boldsymbol{U}_{\boldsymbol{EM}}^{\sigma} & \boldsymbol{0} \\ \boldsymbol{0} & \boldsymbol{0} & \boldsymbol{U}_{\boldsymbol{R}}^{\sigma} \end{pmatrix}\begin{pmatrix} \tilde{\tilde{\boldsymbol{\mathcal{P}}}}{}_{L}^{0\,\sigma}\boldsymbol{U}^{\sigma\dagger}_{L} & \boldsymbol{0} & \boldsymbol{0} \\ \boldsymbol{0} & \boldsymbol{0} & \boldsymbol{0} \\ \boldsymbol{0} & \boldsymbol{0} & \tilde{\tilde{\boldsymbol{\mathcal{P}}}}{}_{R}^{0\,\sigma}\boldsymbol{U}^{\sigma\dagger}_{R} \end{pmatrix}=\begin{pmatrix} \boldsymbol{U}_{\boldsymbol{L}}^{\sigma}\tilde{\tilde{\boldsymbol{\mathcal{P}}}}{}_{L}^{0\,\sigma}\boldsymbol{U}^{\sigma\dagger}_{L} & \boldsymbol{0} & \boldsymbol{0} \\ \boldsymbol{0} & \boldsymbol{0} & \boldsymbol{0} \\ \boldsymbol{0} & \boldsymbol{0} & \boldsymbol{U}_{\boldsymbol{R}}^{\sigma}\tilde{\tilde{\boldsymbol{\mathcal{P}}}}{}_{R}^{0\,\sigma}\boldsymbol{U}^{\sigma\dagger}_{R} \end{pmatrix}\equiv
$$

$$
\equiv\begin{pmatrix} \tilde{\boldsymbol{\mathcal{P}}}{}_{L}^{0\,\sigma} & \boldsymbol{0} & \boldsymbol{0} \\ \boldsymbol{0} & \boldsymbol{0} & \boldsymbol{0} \\ \boldsymbol{0} & \boldsymbol{0} & \tilde{\boldsymbol{\mathcal{P}}}{}_{R}^{0\,\sigma} \end{pmatrix}. \tag{S55}
$$

Summing all four contributions to the driving term we thus obtain:

$$
-\Gamma^{\sigma}\boldsymbol{U}^{\sigma}\begin{pmatrix} \tilde{\tilde{\boldsymbol{\mathcal{P}}}}{}_{L}^{\sigma}-\tilde{\tilde{\boldsymbol{\mathcal{P}}}}{}_{L}^{0\,\sigma} & \frac{1}{2}\tilde{\tilde{\boldsymbol{\mathcal{P}}}}{}_{L,EM}^{\sigma} & \tilde{\tilde{\boldsymbol{\mathcal{P}}}}{}_{LR}^{\sigma} \\ \frac{1}{2}\tilde{\tilde{\boldsymbol{\mathcal{P}}}}{}_{EM,L}^{\sigma} & \boldsymbol{0} & \frac{1}{2}\tilde{\tilde{\boldsymbol{\mathcal{P}}}}{}_{EM,R}^{\sigma} \\ \tilde{\tilde{\boldsymbol{\mathcal{P}}}}{}_{RL}^{\sigma} & \frac{1}{2}\tilde{\tilde{\boldsymbol{\mathcal{P}}}}{}_{R,EM}^{\sigma} & \tilde{\tilde{\boldsymbol{\mathcal{P}}}}{}_{R}^{\sigma}-\tilde{\tilde{\boldsymbol{\mathcal{P}}}}{}_{R}^{0\,\sigma} \end{pmatrix}\boldsymbol{U}^{\sigma\dagger}=
$$

$$
=-\frac{\Gamma^{\sigma}}{2}\begin{pmatrix} \tilde{\boldsymbol{\mathcal{P}}}{}_{L}^{\sigma} & \tilde{\boldsymbol{\mathcal{P}}}{}_{L,EM}^{\sigma} & \tilde{\boldsymbol{\mathcal{P}}}{}_{LR}^{\sigma} \\ \boldsymbol{0} & \boldsymbol{0} & \boldsymbol{0} \\ \tilde{\boldsymbol{\mathcal{P}}}{}_{R,L}^{\sigma} & \tilde{\boldsymbol{\mathcal{P}}}{}_{R,EM}^{\sigma} & \tilde{\boldsymbol{\mathcal{P}}}{}_{R}^{\sigma} \end{pmatrix}-\frac{\Gamma^{\sigma}}{2}\begin{pmatrix} \tilde{\boldsymbol{\mathcal{P}}}{}_{L}^{\sigma} & \boldsymbol{0} & \tilde{\boldsymbol{\mathcal{P}}}{}_{L,R}^{\sigma} \\ \tilde{\boldsymbol{\mathcal{P}}}{}_{EM,L}^{\sigma} & \boldsymbol{0} & \tilde{\boldsymbol{\mathcal{P}}}{}_{EM,R}^{\sigma} \\ \tilde{\boldsymbol{\mathcal{P}}}{}_{R,L}^{\sigma} & \boldsymbol{0} & \tilde{\boldsymbol{\mathcal{P}}}{}_{R}^{\sigma} \end{pmatrix}+\frac{\Gamma^{\sigma}}{2}\begin{pmatrix} \tilde{\boldsymbol{\mathcal{P}}}{}_{L}^{0\,\sigma} & \boldsymbol{0} & \boldsymbol{0} \\ \boldsymbol{0} & \boldsymbol{0} & \boldsymbol{0} \\ \boldsymbol{0} & \boldsymbol{0} & \tilde{\boldsymbol{\mathcal{P}}}{}_{R}^{0\,\sigma} \end{pmatrix}+\frac{\Gamma^{\sigma}}{2}\begin{pmatrix} \tilde{\boldsymbol{\mathcal{P}}}{}_{L}^{0\,\sigma} & \boldsymbol{0} & \boldsymbol{0} \\ \boldsymbol{0} & \boldsymbol{0} & \boldsymbol{0} \\ \boldsymbol{0} & \boldsymbol{0} & \tilde{\boldsymbol{\mathcal{P}}}{}_{R}^{0\,\sigma} \end{pmatrix}=
$$

$$
=-\Gamma^{\sigma}\begin{pmatrix} \tilde{\boldsymbol{\mathcal{P}}}{}_{L}^{\sigma}-\tilde{\boldsymbol{\mathcal{P}}}{}_{L}^{0\,\sigma} & \frac{1}{2}\tilde{\boldsymbol{\mathcal{P}}}{}_{L,EM}^{\sigma} & \tilde{\boldsymbol{\mathcal{P}}}{}_{L,R}^{\sigma} \\ \frac{1}{2}\tilde{\boldsymbol{\mathcal{P}}}{}_{EM,L}^{\sigma} & \boldsymbol{0} & \frac{1}{2}\tilde{\boldsymbol{\mathcal{P}}}{}_{EM,R}^{\sigma} \\ \tilde{\boldsymbol{\mathcal{P}}}{}_{R,L}^{\sigma} & \frac{1}{2}\tilde{\boldsymbol{\mathcal{P}}}{}_{R,EM}^{\sigma} & \tilde{\boldsymbol{\mathcal{P}}}{}_{R}^{\sigma}-\tilde{\boldsymbol{\mathcal{P}}}{}_{R}^{0\,\sigma} \end{pmatrix}. \tag{S56}
$$

We now turn to treat the commutator term in Eq. (S50):

$$
\boldsymbol{U}^{\sigma}\left[\tilde{\tilde{\boldsymbol{\mathcal{H}}}}{}_{KS}^{\sigma},\tilde{\tilde{\boldsymbol{\mathcal{P}}}}{}^{\sigma}\right]\boldsymbol{U}^{\sigma\dagger}=\boldsymbol{U}^{\sigma}\tilde{\tilde{\boldsymbol{\mathcal{H}}}}{}_{KS}^{\sigma}\tilde{\tilde{\boldsymbol{\mathcal{P}}}}{}^{\sigma}\boldsymbol{U}^{\sigma\dagger}-\boldsymbol{U}^{\sigma}\tilde{\tilde{\boldsymbol{\mathcal{P}}}}{}^{\sigma}\tilde{\tilde{\boldsymbol{\mathcal{H}}}}{}_{KS}^{\sigma}\boldsymbol{U}^{\sigma\dagger}=
$$

$$
=\boldsymbol{U}^{\sigma}\boldsymbol{U}^{\sigma\dagger}\tilde{\boldsymbol{\mathcal{H}}}{}_{KS}^{\sigma}\boldsymbol{U}^{\sigma}(\boldsymbol{U}^{\sigma})^{-1}\tilde{\boldsymbol{\mathcal{P}}}{}^{\sigma}\left(\boldsymbol{U}^{\sigma\dagger}\right)^{-1}\boldsymbol{U}^{\sigma\dagger}-\boldsymbol{U}^{\sigma}(\boldsymbol{U}^{\sigma})^{-1}\tilde{\boldsymbol{\mathcal{P}}}{}^{\sigma}\left(\boldsymbol{U}^{\sigma\dagger}\right)^{-1}\boldsymbol{U}^{\sigma\dagger}\tilde{\boldsymbol{\mathcal{H}}}{}_{KS}^{\sigma}\boldsymbol{U}^{\sigma}\boldsymbol{U}^{\sigma\dagger}=
$$

$$
=\boldsymbol{U}^{\sigma}\boldsymbol{U}^{\sigma\dagger}\tilde{\boldsymbol{\mathcal{H}}}{}_{KS}^{\sigma}\tilde{\boldsymbol{\mathcal{P}}}{}^{\sigma}-\tilde{\boldsymbol{\mathcal{P}}}{}^{\sigma}\tilde{\boldsymbol{\mathcal{H}}}{}_{KS}^{\sigma}\boldsymbol{U}^{\sigma}\boldsymbol{U}^{\sigma\dagger}. \tag{S57}
$$

Since $\boldsymbol{U}^{\sigma}$ obeys the relation $\boldsymbol{U}^{\sigma\dagger}\tilde{\boldsymbol{\mathcal{S}}}\boldsymbol{U}^{\sigma}=\boldsymbol{I}$, we may write $\tilde{\boldsymbol{\mathcal{S}}}=\left(\boldsymbol{U}^{\sigma\dagger}\right)^{-1}(\boldsymbol{U}^{\sigma})^{-1}=\left(\boldsymbol{U}^{\sigma}\boldsymbol{U}^{\sigma\dagger}\right)^{-1}$, such that $\boldsymbol{U}^{\sigma}\boldsymbol{U}^{\sigma\dagger}=\tilde{\boldsymbol{\mathcal{S}}}^{-1}$. Therefore, we have:

$$\boldsymbol{U}^{\sigma}\left[\tilde{\tilde{\boldsymbol{\mathcal{H}}}}_{KS}^{\sigma},\tilde{\tilde{\boldsymbol{\mathcal{P}}}}^{\sigma}\right]\boldsymbol{U}^{\sigma\dagger}=\tilde{\boldsymbol{\mathcal{S}}}^{-1}\tilde{\boldsymbol{\mathcal{H}}}_{KS}^{\sigma}\tilde{\boldsymbol{\mathcal{P}}}^{\sigma}-\tilde{\boldsymbol{\mathcal{P}}}^{\sigma}\tilde{\boldsymbol{\mathcal{H}}}_{KS}^{\sigma}\tilde{\boldsymbol{\mathcal{S}}}^{-1}. \tag{S58}$$

Collecting the terms of $\dot{\tilde{\boldsymbol{\mathcal{P}}}}^{\sigma}$ (Eqs. (S50), (S56), and (S58)) we therefore obtain:

$$\dot{\tilde{\boldsymbol{\mathcal{P}}}}^{\sigma}=-i\left(\tilde{\boldsymbol{\mathcal{S}}}^{-1}\tilde{\boldsymbol{\mathcal{H}}}_{KS}^{\sigma}\tilde{\boldsymbol{\mathcal{P}}}^{\sigma}-\tilde{\boldsymbol{\mathcal{P}}}^{\sigma}\tilde{\boldsymbol{\mathcal{H}}}_{KS}^{\sigma}\tilde{\boldsymbol{\mathcal{S}}}^{-1}\right)-\Gamma^{\sigma}\begin{pmatrix}\tilde{\boldsymbol{\mathcal{P}}}_{L}^{\sigma}-\tilde{\boldsymbol{\mathcal{P}}}_{L}^{0^{\sigma}} & \frac{1}{2}\tilde{\boldsymbol{\mathcal{P}}}_{L,EM}^{\sigma} & \tilde{\boldsymbol{\mathcal{P}}}_{L,R}^{\sigma}\\ \frac{1}{2}\tilde{\boldsymbol{\mathcal{P}}}_{EM,L}^{\sigma} & \boldsymbol{0} & \frac{1}{2}\tilde{\boldsymbol{\mathcal{P}}}_{EM,R}^{\sigma}\\ \tilde{\boldsymbol{\mathcal{P}}}_{R,L}^{\sigma} & \frac{1}{2}\tilde{\boldsymbol{\mathcal{P}}}_{R,EM}^{\sigma} & \tilde{\boldsymbol{\mathcal{P}}}_{R}^{\sigma}-\tilde{\boldsymbol{\mathcal{P}}}_{R}^{0^{\sigma}}\end{pmatrix}. \tag{S59}$$

Substituting this in Eq. (S48) for the time derivative of the particle number in the EM section yields:

$$\begin{aligned}&\dot{N}^{\sigma}{}_{EM}=\frac{1}{2}tr_{EM}\left(\dot{\tilde{\boldsymbol{\mathcal{P}}}}^{\sigma}\tilde{\boldsymbol{\mathcal{S}}}+\tilde{\boldsymbol{\mathcal{S}}}\dot{\tilde{\boldsymbol{\mathcal{P}}}}^{\sigma}\right)=\\&=-\frac{i}{2}tr_{EM}\left[\left(\tilde{\boldsymbol{\mathcal{S}}}^{-1}\tilde{\boldsymbol{\mathcal{H}}}_{KS}^{\sigma}\tilde{\boldsymbol{\mathcal{P}}}^{\sigma}-\tilde{\boldsymbol{\mathcal{P}}}^{\sigma}\tilde{\boldsymbol{\mathcal{H}}}_{KS}^{\sigma}\tilde{\boldsymbol{\mathcal{S}}}^{-1}\right)\tilde{\boldsymbol{\mathcal{S}}}+\tilde{\boldsymbol{\mathcal{S}}}\left(\tilde{\boldsymbol{\mathcal{S}}}^{-1}\tilde{\boldsymbol{\mathcal{H}}}_{KS}^{\sigma}\tilde{\boldsymbol{\mathcal{P}}}^{\sigma}-\tilde{\boldsymbol{\mathcal{P}}}^{\sigma}\tilde{\boldsymbol{\mathcal{H}}}_{KS}^{\sigma}\tilde{\boldsymbol{\mathcal{S}}}^{-1}\right)\right]-\\&-\frac{\Gamma^{\sigma}}{2}tr_{EM}\left[\begin{pmatrix}\tilde{\boldsymbol{\mathcal{P}}}_{L}^{\sigma}-\tilde{\boldsymbol{\mathcal{P}}}_{L}^{0^{\sigma}} & \frac{1}{2}\tilde{\boldsymbol{\mathcal{P}}}_{L,EM}^{\sigma} & \tilde{\boldsymbol{\mathcal{P}}}_{L,R}^{\sigma}\\ \frac{1}{2}\tilde{\boldsymbol{\mathcal{P}}}_{EM,L}^{\sigma} & \boldsymbol{0} & \frac{1}{2}\tilde{\boldsymbol{\mathcal{P}}}_{EM,R}^{\sigma}\\ \tilde{\boldsymbol{\mathcal{P}}}_{R,L}^{\sigma} & \frac{1}{2}\tilde{\boldsymbol{\mathcal{P}}}_{R,EM}^{\sigma} & \tilde{\boldsymbol{\mathcal{P}}}_{R}^{\sigma}-\tilde{\boldsymbol{\mathcal{P}}}_{R}^{0^{\sigma}}\end{pmatrix}\tilde{\boldsymbol{\mathcal{S}}}+\tilde{\boldsymbol{\mathcal{S}}}\begin{pmatrix}\tilde{\boldsymbol{\mathcal{P}}}_{L}^{\sigma}-\tilde{\boldsymbol{\mathcal{P}}}_{L}^{0^{\sigma}} & \frac{1}{2}\tilde{\boldsymbol{\mathcal{P}}}_{L,EM}^{\sigma} & \tilde{\boldsymbol{\mathcal{P}}}_{L,R}^{\sigma}\\ \frac{1}{2}\tilde{\boldsymbol{\mathcal{P}}}_{EM,L}^{\sigma} & \boldsymbol{0} & \frac{1}{2}\tilde{\boldsymbol{\mathcal{P}}}_{EM,R}^{\sigma}\\ \tilde{\boldsymbol{\mathcal{P}}}_{R,L}^{\sigma} & \frac{1}{2}\tilde{\boldsymbol{\mathcal{P}}}_{R,EM}^{\sigma} & \tilde{\boldsymbol{\mathcal{P}}}_{R}^{\sigma}-\tilde{\boldsymbol{\mathcal{P}}}_{R}^{0^{\sigma}}\end{pmatrix}\right].\end{aligned} \tag{S60}$$

The overlap matrix in the block diagonal basis assumes the following form:

$$\begin{aligned}&\tilde{\boldsymbol{\mathcal{S}}}\equiv\boldsymbol{U}_{b}^{\dagger}\boldsymbol{\mathcal{S}}\boldsymbol{U}_{b}=\\&=\begin{pmatrix}\boldsymbol{I}_{L} & \boldsymbol{0} & \boldsymbol{0}\\ -\boldsymbol{S}_{EM,L}\boldsymbol{S}_{L}^{-1} & \boldsymbol{I}_{EM} & -\boldsymbol{S}_{EM,R}\boldsymbol{S}_{R}^{-1}\\ \boldsymbol{0} & \boldsymbol{0} & \boldsymbol{I}_{R}\end{pmatrix}\begin{pmatrix}\boldsymbol{S}_{L} & \boldsymbol{S}_{L,EM} & \boldsymbol{0}\\ \boldsymbol{S}_{EM,L} & \boldsymbol{S}_{EM} & \boldsymbol{S}_{EM,R}\\ \boldsymbol{0} & \boldsymbol{S}_{R,EM} & \boldsymbol{S}_{R}\end{pmatrix}\begin{pmatrix}\boldsymbol{I}_{L} & -\boldsymbol{S}_{L}^{-1}\boldsymbol{S}_{L,EM} & \boldsymbol{0}\\ \boldsymbol{0} & \boldsymbol{I}_{EM} & \boldsymbol{0}\\ \boldsymbol{0} & -\boldsymbol{S}_{R}^{-1}\boldsymbol{S}_{R,EM} & \boldsymbol{I}_{R}\end{pmatrix}=\\&=\begin{pmatrix}\boldsymbol{I}_{L} & \boldsymbol{0} & \boldsymbol{0}\\ -\boldsymbol{S}_{EM,L}\boldsymbol{S}_{L}^{-1} & \boldsymbol{I}_{EM} & -\boldsymbol{S}_{EM,R}\boldsymbol{S}_{R}^{-1}\\ \boldsymbol{0} & \boldsymbol{0} & \boldsymbol{I}_{R}\end{pmatrix}\begin{pmatrix}\boldsymbol{S}_{L} & \boldsymbol{0} & \boldsymbol{0}\\ \boldsymbol{S}_{EM,L} & -\boldsymbol{S}_{EM,L}\boldsymbol{S}_{L}^{-1}\boldsymbol{S}_{L,EM}+\boldsymbol{S}_{EM}-\boldsymbol{S}_{EM,R}\boldsymbol{S}_{R}^{-1}\boldsymbol{S}_{R,EM} & \boldsymbol{S}_{EM,R}\\ \boldsymbol{0} & \boldsymbol{0} & \boldsymbol{S}_{R}\end{pmatrix}=\\&=\begin{pmatrix}\boldsymbol{S}_{L} & \boldsymbol{0} & \boldsymbol{0}\\ \boldsymbol{0} & -\boldsymbol{S}_{EM,L}\boldsymbol{S}_{L}^{-1}\boldsymbol{S}_{L,EM}+\boldsymbol{S}_{EM}-\boldsymbol{S}_{EM,R}\boldsymbol{S}_{R}^{-1}\boldsymbol{S}_{R,EM} & \boldsymbol{0}\\ \boldsymbol{0} & \boldsymbol{0} & \boldsymbol{S}_{R}\end{pmatrix}=\\&=\begin{pmatrix}\boldsymbol{S}_{L} & \boldsymbol{0} & \boldsymbol{0}\\ \boldsymbol{0} & \tilde{\boldsymbol{S}}_{EM} & \boldsymbol{0}\\ \boldsymbol{0} & \boldsymbol{0} & \boldsymbol{S}_{R}\end{pmatrix},\end{aligned} \tag{S61}$$

where we have defined $\tilde{\boldsymbol{S}}_{EM}\equiv\boldsymbol{S}_{EM}-\boldsymbol{S}_{EM,L}\boldsymbol{S}_{L}^{-1}\boldsymbol{S}_{L,EM}-\boldsymbol{S}_{EM,R}\boldsymbol{S}_{R}^{-1}\boldsymbol{S}_{R,EM}$. We can now use this to evaluate the different terms appearing in Eq. (S60). Starting from the driving term contributions we have:

$$\begin{pmatrix} \widetilde{\boldsymbol{\mathcal{P}}}_L^\sigma - \widetilde{\boldsymbol{\mathcal{P}}}_L^{0\,\sigma} & \frac{1}{2}\widetilde{\boldsymbol{\mathcal{P}}}_{L,EM}^\sigma & \widetilde{\boldsymbol{\mathcal{P}}}_{L,R}^\sigma \\ \frac{1}{2}\widetilde{\boldsymbol{\mathcal{P}}}_{EM,L}^\sigma & \mathbf{0} & \frac{1}{2}\widetilde{\boldsymbol{\mathcal{P}}}_{EM,R}^\sigma \\ \widetilde{\boldsymbol{\mathcal{P}}}_{R,L}^\sigma & \frac{1}{2}\widetilde{\boldsymbol{\mathcal{P}}}_{R,EM}^\sigma & \widetilde{\boldsymbol{\mathcal{P}}}_R^\sigma - \widetilde{\boldsymbol{\mathcal{P}}}_R^{0\,\sigma} \end{pmatrix} \widetilde{\boldsymbol{\mathcal{S}}} = \begin{pmatrix} \widetilde{\boldsymbol{\mathcal{P}}}_L^\sigma - \widetilde{\boldsymbol{\mathcal{P}}}_L^{0\,\sigma} & \frac{1}{2}\widetilde{\boldsymbol{\mathcal{P}}}_{L,EM}^\sigma & \widetilde{\boldsymbol{\mathcal{P}}}_{L,R}^\sigma \\ \frac{1}{2}\widetilde{\boldsymbol{\mathcal{P}}}_{EM,L}^\sigma & \mathbf{0} & \frac{1}{2}\widetilde{\boldsymbol{\mathcal{P}}}_{EM,R}^\sigma \\ \widetilde{\boldsymbol{\mathcal{P}}}_{R,L}^\sigma & \frac{1}{2}\widetilde{\boldsymbol{\mathcal{P}}}_{R,EM}^\sigma & \widetilde{\boldsymbol{\mathcal{P}}}_R^\sigma - \widetilde{\boldsymbol{\mathcal{P}}}_R^{0\,\sigma} \end{pmatrix} \begin{pmatrix} \boldsymbol{S}_L & \mathbf{0} & \mathbf{0} \\ \mathbf{0} & \widetilde{\boldsymbol{S}}_{EM} & \mathbf{0} \\ \mathbf{0} & \mathbf{0} & \boldsymbol{S}_R \end{pmatrix} =$$

$$= \begin{pmatrix} \left(\widetilde{\boldsymbol{\mathcal{P}}}_L^\sigma - \widetilde{\boldsymbol{\mathcal{P}}}_L^{0\,\sigma}\right)\boldsymbol{S}_L & \frac{1}{2}\widetilde{\boldsymbol{\mathcal{P}}}_{L,EM}^\sigma \widetilde{\boldsymbol{S}}_{EM} & \widetilde{\boldsymbol{\mathcal{P}}}_{L,R}^\sigma \boldsymbol{S}_R \\ \frac{1}{2}\widetilde{\boldsymbol{\mathcal{P}}}_{EM,L}^\sigma \boldsymbol{S}_L & \mathbf{0} & \frac{1}{2}\widetilde{\boldsymbol{\mathcal{P}}}_{EM,R}^\sigma \boldsymbol{S}_R \\ \widetilde{\boldsymbol{\mathcal{P}}}_{R,L}^\sigma \boldsymbol{S}_L & \frac{1}{2}\widetilde{\boldsymbol{\mathcal{P}}}_{R,EM}^\sigma \widetilde{\boldsymbol{S}}_{EM} & \left(\widetilde{\boldsymbol{\mathcal{P}}}_R^\sigma - \widetilde{\boldsymbol{\mathcal{P}}}_R^{0\,\sigma}\right)\boldsymbol{S}_R \end{pmatrix}, \tag{S62}$$

and

$$\widetilde{\boldsymbol{\mathcal{S}}} \begin{pmatrix} \widetilde{\boldsymbol{\mathcal{P}}}_L^\sigma - \widetilde{\boldsymbol{\mathcal{P}}}_L^{0\,\sigma} & \frac{1}{2}\widetilde{\boldsymbol{\mathcal{P}}}_{L,EM}^\sigma & \widetilde{\boldsymbol{\mathcal{P}}}_{L,R}^\sigma \\ \frac{1}{2}\widetilde{\boldsymbol{\mathcal{P}}}_{EM,L}^\sigma & \mathbf{0} & \frac{1}{2}\widetilde{\boldsymbol{\mathcal{P}}}_{EM,R}^\sigma \\ \widetilde{\boldsymbol{\mathcal{P}}}_{R,L}^\sigma & \frac{1}{2}\widetilde{\boldsymbol{\mathcal{P}}}_{R,EM}^\sigma & \widetilde{\boldsymbol{\mathcal{P}}}_R^\sigma - \widetilde{\boldsymbol{\mathcal{P}}}_R^{0\,\sigma} \end{pmatrix} = \begin{pmatrix} \boldsymbol{S}_L & \mathbf{0} & \mathbf{0} \\ \mathbf{0} & \widetilde{\boldsymbol{S}}_{EM} & \mathbf{0} \\ \mathbf{0} & \mathbf{0} & \boldsymbol{S}_R \end{pmatrix} \begin{pmatrix} \widetilde{\boldsymbol{\mathcal{P}}}_L^\sigma - \widetilde{\boldsymbol{\mathcal{P}}}_L^{0\,\sigma} & \frac{1}{2}\widetilde{\boldsymbol{\mathcal{P}}}_{L,EM}^\sigma & \widetilde{\boldsymbol{\mathcal{P}}}_{L,R}^\sigma \\ \frac{1}{2}\widetilde{\boldsymbol{\mathcal{P}}}_{EM,L}^\sigma & \mathbf{0} & \frac{1}{2}\widetilde{\boldsymbol{\mathcal{P}}}_{EM,R}^\sigma \\ \widetilde{\boldsymbol{\mathcal{P}}}_{R,L}^\sigma & \frac{1}{2}\widetilde{\boldsymbol{\mathcal{P}}}_{R,EM}^\sigma & \widetilde{\boldsymbol{\mathcal{P}}}_R^\sigma - \widetilde{\boldsymbol{\mathcal{P}}}_R^{0\,\sigma} \end{pmatrix} =$$

$$= \begin{pmatrix} \boldsymbol{S}_L\left(\widetilde{\boldsymbol{\mathcal{P}}}_L^\sigma - \widetilde{\boldsymbol{\mathcal{P}}}_L^{0\,\sigma}\right) & \frac{1}{2}\widetilde{\boldsymbol{S}}_L \widetilde{\boldsymbol{\mathcal{P}}}_{L,EM}^\sigma & \boldsymbol{S}_L \widetilde{\boldsymbol{\mathcal{P}}}_{L,R}^\sigma \\ \frac{1}{2}\boldsymbol{S}_{EM} \widetilde{\boldsymbol{\mathcal{P}}}_{EM,L}^\sigma & \mathbf{0} & \frac{1}{2}\boldsymbol{S}_{EM} \widetilde{\boldsymbol{\mathcal{P}}}_{EM,R}^\sigma \\ \boldsymbol{S}_R \widetilde{\boldsymbol{\mathcal{P}}}_{R,L}^\sigma & \frac{1}{2}\widetilde{\boldsymbol{S}}_R \widetilde{\boldsymbol{\mathcal{P}}}_{R,EM}^\sigma & \boldsymbol{S}_R\left(\widetilde{\boldsymbol{\mathcal{P}}}_R^\sigma - \widetilde{\boldsymbol{\mathcal{P}}}_R^{0\,\sigma}\right) \end{pmatrix}. \tag{S63}$$

Altogether, the driving term contribution to $\dot{N}_{EM}$ in Eq. (S60) is given by (Eqs. (S62) and (S63)):

$$-\frac{\Gamma}{2} tr_{EM}\left[\begin{pmatrix} \widetilde{\boldsymbol{\mathcal{P}}}_L^\sigma - \widetilde{\boldsymbol{\mathcal{P}}}_L^{0\,\sigma} & \frac{1}{2}\widetilde{\boldsymbol{\mathcal{P}}}_{L,EM}^\sigma & \widetilde{\boldsymbol{\mathcal{P}}}_{L,R}^\sigma \\ \frac{1}{2}\widetilde{\boldsymbol{\mathcal{P}}}_{EM,L}^\sigma & \mathbf{0} & \frac{1}{2}\widetilde{\boldsymbol{\mathcal{P}}}_{EM,R}^\sigma \\ \widetilde{\boldsymbol{\mathcal{P}}}_{R,L}^\sigma & \frac{1}{2}\widetilde{\boldsymbol{\mathcal{P}}}_{R,EM}^\sigma & \widetilde{\boldsymbol{\mathcal{P}}}_R^\sigma - \widetilde{\boldsymbol{\mathcal{P}}}_R^{0\,\sigma} \end{pmatrix} \widetilde{\boldsymbol{\mathcal{S}}} + \widetilde{\boldsymbol{\mathcal{S}}} \begin{pmatrix} \widetilde{\boldsymbol{\mathcal{P}}}_L^\sigma - \widetilde{\boldsymbol{\mathcal{P}}}_L^{0\,\sigma} & \frac{1}{2}\widetilde{\boldsymbol{\mathcal{P}}}_{L,EM}^\sigma & \widetilde{\boldsymbol{\mathcal{P}}}_{L,R}^\sigma \\ \frac{1}{2}\widetilde{\boldsymbol{\mathcal{P}}}_{EM,L}^\sigma & \mathbf{0} & \frac{1}{2}\widetilde{\boldsymbol{\mathcal{P}}}_{EM,R}^\sigma \\ \widetilde{\boldsymbol{\mathcal{P}}}_{R,L}^\sigma & \frac{1}{2}\widetilde{\boldsymbol{\mathcal{P}}}_{R,EM}^\sigma & \widetilde{\boldsymbol{\mathcal{P}}}_R^\sigma - \widetilde{\boldsymbol{\mathcal{P}}}_R^{0\,\sigma} \end{pmatrix} \right] =$$

$$-\frac{\Gamma}{2} tr_{EM}\left[\begin{pmatrix} \left(\widetilde{\boldsymbol{\mathcal{P}}}_L^\sigma - \widetilde{\boldsymbol{\mathcal{P}}}_L^{0\,\sigma}\right)\boldsymbol{S}_L + \boldsymbol{S}_L\left(\widetilde{\boldsymbol{\mathcal{P}}}_L^\sigma - \widetilde{\boldsymbol{\mathcal{P}}}_L^{0\,\sigma}\right) & \frac{1}{2}\left(\widetilde{\boldsymbol{\mathcal{P}}}_{L,EM}^\sigma \widetilde{\boldsymbol{S}}_{EM} + \widetilde{\boldsymbol{S}}_L \widetilde{\boldsymbol{\mathcal{P}}}_{L,EM}^\sigma\right) & \widetilde{\boldsymbol{\mathcal{P}}}_{L,R}^\sigma \boldsymbol{S}_R + \boldsymbol{S}_L \widetilde{\boldsymbol{\mathcal{P}}}_{L,R}^\sigma \\ \frac{1}{2}\left(\widetilde{\boldsymbol{\mathcal{P}}}_{EM,L}^\sigma \boldsymbol{S}_L + \boldsymbol{S}_{EM} \widetilde{\boldsymbol{\mathcal{P}}}_{EM,L}^\sigma\right) & \mathbf{0} & \frac{1}{2}\left(\widetilde{\boldsymbol{\mathcal{P}}}_{EM,R}^\sigma \boldsymbol{S}_R + \boldsymbol{S}_{EM} \widetilde{\boldsymbol{\mathcal{P}}}_{EM,R}^\sigma\right) \\ \widetilde{\boldsymbol{\mathcal{P}}}_{R,L}^\sigma \boldsymbol{S}_L + \boldsymbol{S}_R \widetilde{\boldsymbol{\mathcal{P}}}_{R,L}^\sigma & \frac{1}{2}\left(\widetilde{\boldsymbol{\mathcal{P}}}_{R,EM}^\sigma \widetilde{\boldsymbol{S}}_{EM} + \widetilde{\boldsymbol{S}}_R \widetilde{\boldsymbol{\mathcal{P}}}_{R,EM}^\sigma\right) & \left(\widetilde{\boldsymbol{\mathcal{P}}}_R^\sigma - \widetilde{\boldsymbol{\mathcal{P}}}_R^{0\,\sigma}\right)\boldsymbol{S}_R + \boldsymbol{S}_R\left(\widetilde{\boldsymbol{\mathcal{P}}}_R^\sigma - \widetilde{\boldsymbol{\mathcal{P}}}_R^{0\,\sigma}\right) \end{pmatrix} \right]$$

$$= -\frac{\Gamma}{2} tr_{EM} \begin{pmatrix} \left[\left(\widetilde{\boldsymbol{\mathcal{P}}}_L^\sigma - \widetilde{\boldsymbol{\mathcal{P}}}_L^{0\,\sigma}\right), \boldsymbol{S}_L\right]_+ & \frac{1}{2}\left(\widetilde{\boldsymbol{\mathcal{P}}}_{L,EM}^\sigma \widetilde{\boldsymbol{S}}_{EM} + \widetilde{\boldsymbol{S}}_L \widetilde{\boldsymbol{\mathcal{P}}}_{L,EM}^\sigma\right) & \widetilde{\boldsymbol{\mathcal{P}}}_{L,R}^\sigma \boldsymbol{S}_R + \boldsymbol{S}_L \widetilde{\boldsymbol{\mathcal{P}}}_{L,R}^\sigma \\ \frac{1}{2}\left(\widetilde{\boldsymbol{\mathcal{P}}}_{EM,L}^\sigma \boldsymbol{S}_L + \boldsymbol{S}_{EM} \widetilde{\boldsymbol{\mathcal{P}}}_{EM,L}^\sigma\right) & \mathbf{0} & \frac{1}{2}\left(\widetilde{\boldsymbol{\mathcal{P}}}_{EM,R}^\sigma \boldsymbol{S}_R + \boldsymbol{S}_{EM} \widetilde{\boldsymbol{\mathcal{P}}}_{EM,R}^\sigma\right) \\ \widetilde{\boldsymbol{\mathcal{P}}}_{R,L}^\sigma \boldsymbol{S}_L + \boldsymbol{S}_R \widetilde{\boldsymbol{\mathcal{P}}}_{R,L}^\sigma & \frac{1}{2}\left(\widetilde{\boldsymbol{\mathcal{P}}}_{R,EM}^\sigma \widetilde{\boldsymbol{S}}_{EM} + \widetilde{\boldsymbol{S}}_R \widetilde{\boldsymbol{\mathcal{P}}}_{R,EM}^\sigma\right) & \left[\left(\widetilde{\boldsymbol{\mathcal{P}}}_R^\sigma - \widetilde{\boldsymbol{\mathcal{P}}}_R^{0\,\sigma}\right), \boldsymbol{S}_R\right]_+ \end{pmatrix} \tag{S64}$$

$$= 0,$$

where $[\boldsymbol{A}, \boldsymbol{B}]_+ = \boldsymbol{AB} + \boldsymbol{BA}$ is the anticommutator. We therefore see that the driving term does not contribute to the expression of the total instantaneous current flowing through the EM section, as expected.

Finally, we evaluate the contribution of the first term on the right-hand side of Eq. (S60):

$$-\frac{i}{2}tr_{EM}\left[\left(\tilde{\boldsymbol{\mathcal{S}}}^{-1}\widetilde{\boldsymbol{\mathcal{H}}}_{KS}^{\sigma}\widetilde{\boldsymbol{\mathcal{P}}}^{\sigma}-\widetilde{\boldsymbol{\mathcal{P}}}^{\sigma}\widetilde{\boldsymbol{\mathcal{H}}}_{KS}^{\sigma}\tilde{\boldsymbol{\mathcal{S}}}^{-1}\right)\tilde{\boldsymbol{\mathcal{S}}}+\tilde{\boldsymbol{\mathcal{S}}}\left(\tilde{\boldsymbol{\mathcal{S}}}^{-1}\widetilde{\boldsymbol{\mathcal{H}}}_{KS}^{\sigma}\widetilde{\boldsymbol{\mathcal{P}}}^{\sigma}-\widetilde{\boldsymbol{\mathcal{P}}}^{\sigma}\widetilde{\boldsymbol{\mathcal{H}}}_{KS}^{\sigma}\tilde{\boldsymbol{\mathcal{S}}}^{-1}\right)\right]=$$

$$=-\frac{i}{2}tr_{EM}\left[\tilde{\boldsymbol{\mathcal{S}}}^{-1}\widetilde{\boldsymbol{\mathcal{H}}}_{KS}^{\sigma}\widetilde{\boldsymbol{\mathcal{P}}}^{\sigma}\tilde{\boldsymbol{\mathcal{S}}}-\tilde{\boldsymbol{\mathcal{S}}}\widetilde{\boldsymbol{\mathcal{P}}}^{\sigma}\widetilde{\boldsymbol{\mathcal{H}}}_{KS}^{\sigma}\tilde{\boldsymbol{\mathcal{S}}}^{-1}+\widetilde{\boldsymbol{\mathcal{H}}}_{KS}^{\sigma}\widetilde{\boldsymbol{\mathcal{P}}}^{\sigma}-\widetilde{\boldsymbol{\mathcal{P}}}^{\sigma}\widetilde{\boldsymbol{\mathcal{H}}}_{KS}^{\sigma}\right]. \tag{S65}$$

The first term reads:

$$\tilde{\boldsymbol{\mathcal{S}}}^{-1}\widetilde{\boldsymbol{\mathcal{H}}}_{KS}^{\sigma}\widetilde{\boldsymbol{\mathcal{P}}}^{\sigma}\tilde{\boldsymbol{\mathcal{S}}}=$$

$$=\begin{pmatrix}\boldsymbol{S}_L^{-1} & \boldsymbol{0} & \boldsymbol{0}\\ \boldsymbol{0} & \tilde{\boldsymbol{S}}_{EM}^{-1} & \boldsymbol{0}\\ \boldsymbol{0} & \boldsymbol{0} & \boldsymbol{S}_R^{-1}\end{pmatrix}\begin{pmatrix}\boldsymbol{H}_L^{\sigma} & \widetilde{\boldsymbol{V}}_{L,EM}^{\sigma} & \boldsymbol{0}\\ \widetilde{\boldsymbol{V}}_{EM,L}^{\sigma} & \widetilde{\boldsymbol{H}}_{EM}^{\sigma} & \widetilde{\boldsymbol{V}}_{EM,R}^{\sigma}\\ \boldsymbol{0} & \widetilde{\boldsymbol{V}}_{R,EM}^{\sigma} & \boldsymbol{H}_R^{\sigma}\end{pmatrix}\begin{pmatrix}\widetilde{\boldsymbol{\mathcal{P}}}_{L\,L}^{\sigma} & \widetilde{\boldsymbol{\mathcal{P}}}_{L,EM}^{\sigma} & \widetilde{\boldsymbol{\mathcal{P}}}_{LR}^{\sigma}\\ \widetilde{\boldsymbol{\mathcal{P}}}_{EM,L}^{\sigma} & \widetilde{\boldsymbol{\mathcal{P}}}_{EM}^{\sigma} & \widetilde{\boldsymbol{\mathcal{P}}}_{EM,R}^{\sigma}\\ \widetilde{\boldsymbol{\mathcal{P}}}_{R,L}^{\sigma} & \widetilde{\boldsymbol{\mathcal{P}}}_{R,EM}^{\sigma} & \widetilde{\boldsymbol{\mathcal{P}}}_{R}^{\sigma}\end{pmatrix}\begin{pmatrix}\boldsymbol{S}_L & \boldsymbol{0} & \boldsymbol{0}\\ \boldsymbol{0} & \tilde{\boldsymbol{S}}_{EM} & \boldsymbol{0}\\ \boldsymbol{0} & \boldsymbol{0} & \boldsymbol{S}_R\end{pmatrix}=$$

$$=\begin{pmatrix}\boldsymbol{S}_L^{-1}\boldsymbol{H}_L^{\sigma} & \boldsymbol{S}_L^{-1}\widetilde{\boldsymbol{V}}_{L,EM}^{\sigma} & \boldsymbol{0}\\ \tilde{\boldsymbol{S}}_{EM}^{-1}\widetilde{\boldsymbol{V}}_{EM,L}^{\sigma} & \tilde{\boldsymbol{S}}_{EM}^{-1}\widetilde{\boldsymbol{H}}_{EM}^{\sigma} & \tilde{\boldsymbol{S}}_{EM}^{-1}\widetilde{\boldsymbol{V}}_{EM,R}^{\sigma}\\ \boldsymbol{0} & \boldsymbol{S}_R^{-1}\widetilde{\boldsymbol{V}}_{R,EM}^{\sigma} & \boldsymbol{S}_R^{-1}\boldsymbol{H}_R^{\sigma}\end{pmatrix}\begin{pmatrix}\widetilde{\boldsymbol{\mathcal{P}}}_{L}^{\sigma}\boldsymbol{S}_L & \widetilde{\boldsymbol{\mathcal{P}}}_{L,EM}^{\sigma}\tilde{\boldsymbol{S}}_{EM} & \widetilde{\boldsymbol{\mathcal{P}}}_{LR}^{\sigma}\boldsymbol{S}_R\\ \widetilde{\boldsymbol{\mathcal{P}}}_{EM,L}^{\sigma}\boldsymbol{S}_L & \widetilde{\boldsymbol{\mathcal{P}}}_{EM}^{\sigma}\tilde{\boldsymbol{S}}_{EM} & \widetilde{\boldsymbol{\mathcal{P}}}_{EM,R}^{\sigma}\boldsymbol{S}_R\\ \widetilde{\boldsymbol{\mathcal{P}}}_{R,L}^{\sigma}\boldsymbol{S}_L & \widetilde{\boldsymbol{\mathcal{P}}}_{R,EM}^{\sigma}\tilde{\boldsymbol{S}}_{EM} & \widetilde{\boldsymbol{\mathcal{P}}}_{R}^{\sigma}\boldsymbol{S}_R\end{pmatrix}= \tag{S66}$$

$$=\begin{pmatrix}\boldsymbol{S}_L^{-1}\boldsymbol{H}_L^{\sigma}\widetilde{\boldsymbol{\mathcal{P}}}_L^{\sigma}\boldsymbol{S}_L+\boldsymbol{S}_L^{-1}\widetilde{\boldsymbol{V}}_{L,EM}^{\sigma}\widetilde{\boldsymbol{\mathcal{P}}}_{EM,L}^{\sigma}\boldsymbol{S}_L & \boldsymbol{S}_L^{-1}\boldsymbol{H}_L^{\sigma}\widetilde{\boldsymbol{\mathcal{P}}}_{L,EM}^{\sigma}\tilde{\boldsymbol{S}}_{EM}+\boldsymbol{S}_L^{-1}\widetilde{\boldsymbol{V}}_{L,EM}^{\sigma}\widetilde{\boldsymbol{\mathcal{P}}}_{EM}^{\sigma}\tilde{\boldsymbol{S}}_{EM} & \boldsymbol{S}_L^{-1}\boldsymbol{H}_L^{\sigma}\widetilde{\boldsymbol{\mathcal{P}}}_{LR}^{\sigma}\boldsymbol{S}_R+\boldsymbol{S}_L^{-1}\widetilde{\boldsymbol{V}}_{L,EM}^{\sigma}\widetilde{\boldsymbol{\mathcal{P}}}_{EM,R}^{\sigma}\boldsymbol{S}_R\\ \tilde{\boldsymbol{S}}_{EM}^{-1}\widetilde{\boldsymbol{V}}_{EM,L}^{\sigma}\widetilde{\boldsymbol{\mathcal{P}}}_L^{\sigma}\boldsymbol{S}_L+\tilde{\boldsymbol{S}}_{EM}^{-1}\widetilde{\boldsymbol{H}}_{EM}^{\sigma}\widetilde{\boldsymbol{\mathcal{P}}}_{EM,L}^{\sigma}\boldsymbol{S}_L+\tilde{\boldsymbol{S}}_{EM}^{-1}\widetilde{\boldsymbol{V}}_{EM,R}^{\sigma}\widetilde{\boldsymbol{\mathcal{P}}}_{R,L}^{\sigma}\boldsymbol{S}_L & \tilde{\boldsymbol{S}}_{EM}^{-1}\widetilde{\boldsymbol{V}}_{EM,L}^{\sigma}\widetilde{\boldsymbol{\mathcal{P}}}_{L,EM}^{\sigma}\tilde{\boldsymbol{S}}_{EM}+\tilde{\boldsymbol{S}}_{EM}^{-1}\widetilde{\boldsymbol{H}}_{EM}^{\sigma}\widetilde{\boldsymbol{\mathcal{P}}}_{EM}^{\sigma}\tilde{\boldsymbol{S}}_{EM}+\tilde{\boldsymbol{S}}_{EM}^{-1}\widetilde{\boldsymbol{V}}_{EM,R}^{\sigma}\widetilde{\boldsymbol{\mathcal{P}}}_{R,EM}^{\sigma}\tilde{\boldsymbol{S}}_{EM} & \tilde{\boldsymbol{S}}_{EM}^{-1}\widetilde{\boldsymbol{V}}_{EM,L}^{\sigma}\widetilde{\boldsymbol{\mathcal{P}}}_{LR}^{\sigma}\boldsymbol{S}_R+\tilde{\boldsymbol{S}}_{EM}^{-1}\widetilde{\boldsymbol{H}}_{EM}^{\sigma}\widetilde{\boldsymbol{\mathcal{P}}}_{EM,L}^{\sigma}\boldsymbol{S}_R+\tilde{\boldsymbol{S}}_{EM}^{-1}\widetilde{\boldsymbol{V}}_{EM,R}^{\sigma}\widetilde{\boldsymbol{\mathcal{P}}}_{R}^{\sigma}\boldsymbol{S}_R\\ \boldsymbol{S}_R^{-1}\widetilde{\boldsymbol{V}}_{R,EM}^{\sigma}\widetilde{\boldsymbol{\mathcal{P}}}_{EM,L}^{\sigma}\boldsymbol{S}_L+\boldsymbol{S}_R^{-1}\boldsymbol{H}_R^{\sigma}\widetilde{\boldsymbol{\mathcal{P}}}_{R,L}^{\sigma}\boldsymbol{S}_L & \boldsymbol{S}_R^{-1}\widetilde{\boldsymbol{V}}_{R,EM}^{\sigma}\widetilde{\boldsymbol{\mathcal{P}}}_{EM}^{\sigma}\tilde{\boldsymbol{S}}_{EM}+\boldsymbol{S}_R^{-1}\boldsymbol{H}_R^{\sigma}\widetilde{\boldsymbol{\mathcal{P}}}_{R,EM}^{\sigma}\tilde{\boldsymbol{S}}_{EM} & \boldsymbol{S}_R^{-1}\widetilde{\boldsymbol{V}}_{R,EM}^{\sigma}\widetilde{\boldsymbol{\mathcal{P}}}_{EM,R}^{\sigma}\boldsymbol{S}_R+\boldsymbol{S}_R^{-1}\boldsymbol{H}_R^{\sigma}\widetilde{\boldsymbol{\mathcal{P}}}_{R}^{\sigma}\boldsymbol{S}_R\end{pmatrix},$$

whose *EM* block is:

$$\left(\tilde{\boldsymbol{\mathcal{S}}}^{-1}\widetilde{\boldsymbol{\mathcal{H}}}_{KS}^{\sigma}\widetilde{\boldsymbol{\mathcal{P}}}^{\sigma}\tilde{\boldsymbol{\mathcal{S}}}\right)=\tilde{\boldsymbol{S}}_{EM}^{-1}\widetilde{\boldsymbol{V}}_{EM,L}^{\sigma}\widetilde{\boldsymbol{\mathcal{P}}}_{L,EM}^{\sigma}\tilde{\boldsymbol{S}}_{EM}+\tilde{\boldsymbol{S}}_{EM}^{-1}\widetilde{\boldsymbol{H}}_{EM}^{\sigma}\widetilde{\boldsymbol{\mathcal{P}}}_{EM}^{\sigma}\tilde{\boldsymbol{S}}_{EM}+\tilde{\boldsymbol{S}}_{EM}^{-1}\widetilde{\boldsymbol{V}}_{EM,R}^{\sigma}\widetilde{\boldsymbol{\mathcal{P}}}_{R,EM}^{\sigma}\tilde{\boldsymbol{S}}_{EM}. \tag{S67}$$

The second term reads:

$$\tilde{\boldsymbol{\mathcal{S}}}\widetilde{\boldsymbol{\mathcal{P}}}^{\sigma}\widetilde{\boldsymbol{\mathcal{H}}}_{KS}^{\sigma}\tilde{\boldsymbol{\mathcal{S}}}^{-1}=$$

$$=\begin{pmatrix}\boldsymbol{S}_L & \boldsymbol{0} & \boldsymbol{0}\\ \boldsymbol{0} & \tilde{\boldsymbol{S}}_{EM} & \boldsymbol{0}\\ \boldsymbol{0} & \boldsymbol{0} & \boldsymbol{S}_R\end{pmatrix}\begin{pmatrix}\widetilde{\boldsymbol{\mathcal{P}}}_{L}^{\sigma} & \widetilde{\boldsymbol{\mathcal{P}}}_{L,EM}^{\sigma} & \widetilde{\boldsymbol{\mathcal{P}}}_{LR}^{\sigma}\\ \widetilde{\boldsymbol{\mathcal{P}}}_{EM,L}^{\sigma} & \widetilde{\boldsymbol{\mathcal{P}}}_{EM}^{\sigma} & \widetilde{\boldsymbol{\mathcal{P}}}_{EM,R}^{\sigma}\\ \widetilde{\boldsymbol{\mathcal{P}}}_{R,L}^{\sigma} & \widetilde{\boldsymbol{\mathcal{P}}}_{R,EM}^{\sigma} & \widetilde{\boldsymbol{\mathcal{P}}}_{R}^{\sigma}\end{pmatrix}\begin{pmatrix}\boldsymbol{H}_L^{\sigma} & \widetilde{\boldsymbol{V}}_{L,EM}^{\sigma} & \boldsymbol{0}\\ \widetilde{\boldsymbol{V}}_{EM,L}^{\sigma} & \widetilde{\boldsymbol{H}}_{EM}^{\sigma} & \widetilde{\boldsymbol{V}}_{EM,R}^{\sigma}\\ \boldsymbol{0} & \widetilde{\boldsymbol{V}}_{R,EM}^{\sigma} & \boldsymbol{H}_R^{\sigma}\end{pmatrix}\begin{pmatrix}\boldsymbol{S}_L^{-1} & \boldsymbol{0} & \boldsymbol{0}\\ \boldsymbol{0} & \tilde{\boldsymbol{S}}_{EM}^{-1} & \boldsymbol{0}\\ \boldsymbol{0} & \boldsymbol{0} & \boldsymbol{S}_R^{-1}\end{pmatrix}=$$

$$=\begin{pmatrix}\boldsymbol{S}_L\widetilde{\boldsymbol{\mathcal{P}}}_{L}^{\sigma} & \boldsymbol{S}_L\widetilde{\boldsymbol{\mathcal{P}}}_{L,EM}^{\sigma} & \boldsymbol{S}_L\widetilde{\boldsymbol{\mathcal{P}}}_{LR}^{\sigma}\\ \tilde{\boldsymbol{S}}_{EM}\widetilde{\boldsymbol{\mathcal{P}}}_{EM,L}^{\sigma} & \tilde{\boldsymbol{S}}_{EM}\widetilde{\boldsymbol{\mathcal{P}}}_{EM}^{\sigma} & \tilde{\boldsymbol{S}}_{EM}\widetilde{\boldsymbol{\mathcal{P}}}_{EM,R}^{\sigma}\\ \boldsymbol{S}_R\widetilde{\boldsymbol{\mathcal{P}}}_{R,L}^{\sigma} & \boldsymbol{S}_R\widetilde{\boldsymbol{\mathcal{P}}}_{R,EM}^{\sigma} & \boldsymbol{S}_R\widetilde{\boldsymbol{\mathcal{P}}}_{R}^{\sigma}\end{pmatrix}\begin{pmatrix}\boldsymbol{H}_L^{\sigma}\boldsymbol{S}_L^{-1} & \widetilde{\boldsymbol{V}}_{L,EM}^{\sigma}\tilde{\boldsymbol{S}}_{EM}^{-1} & \boldsymbol{0}\\ \widetilde{\boldsymbol{V}}_{EM,L}^{\sigma}\boldsymbol{S}_L^{-1} & \widetilde{\boldsymbol{H}}_{EM}^{\sigma}\tilde{\boldsymbol{S}}_{EM}^{-1} & \widetilde{\boldsymbol{V}}_{EM,R}^{\sigma}\boldsymbol{S}_R^{-1}\\ \boldsymbol{0} & \widetilde{\boldsymbol{V}}_{R,EM}^{\sigma}\tilde{\boldsymbol{S}}_{EM}^{-1} & \boldsymbol{H}_R^{\sigma}\boldsymbol{S}_R^{-1}\end{pmatrix}= \tag{S68}$$

$$=\begin{pmatrix}\boldsymbol{S}_L\widetilde{\boldsymbol{\mathcal{P}}}_L^{\sigma}\boldsymbol{H}_L^{\sigma}\boldsymbol{S}_L^{-1}+\boldsymbol{S}_L\widetilde{\boldsymbol{\mathcal{P}}}_{L,EM}^{\sigma}\widetilde{\boldsymbol{V}}_{EM,L}^{\sigma}\boldsymbol{S}_L^{-1} & \boldsymbol{S}_L\widetilde{\boldsymbol{\mathcal{P}}}_L^{\sigma}\widetilde{\boldsymbol{V}}_{L,EM}^{\sigma}\tilde{\boldsymbol{S}}_{EM}^{-1}+\boldsymbol{S}_L\widetilde{\boldsymbol{\mathcal{P}}}_{L,EM}^{\sigma}\widetilde{\boldsymbol{H}}_{EM}^{\sigma}\tilde{\boldsymbol{S}}_{EM}^{-1}+\boldsymbol{S}_L\widetilde{\boldsymbol{\mathcal{P}}}_{LR}^{\sigma}\widetilde{\boldsymbol{V}}_{R,EM}^{\sigma}\tilde{\boldsymbol{S}}_{EM}^{-1} & \boldsymbol{S}_L\widetilde{\boldsymbol{\mathcal{P}}}_{L,EM}^{\sigma}\widetilde{\boldsymbol{V}}_{EM,R}^{\sigma}\boldsymbol{S}_R^{-1}+\boldsymbol{S}_L\widetilde{\boldsymbol{\mathcal{P}}}_{LR}^{\sigma}\boldsymbol{H}_R^{\sigma}\boldsymbol{S}_R^{-1}\\ \tilde{\boldsymbol{S}}_{EM}\widetilde{\boldsymbol{\mathcal{P}}}_{EM,L}^{\sigma}\boldsymbol{H}_L^{\sigma}\boldsymbol{S}_L^{-1}+\tilde{\boldsymbol{S}}_{EM}\widetilde{\boldsymbol{\mathcal{P}}}_{EM}^{\sigma}\widetilde{\boldsymbol{V}}_{EM,L}^{\sigma}\boldsymbol{S}_L^{-1} & \tilde{\boldsymbol{S}}_{EM}\widetilde{\boldsymbol{\mathcal{P}}}_{EM,L}^{\sigma}\widetilde{\boldsymbol{V}}_{L,EM}^{\sigma}\tilde{\boldsymbol{S}}_{EM}^{-1}+\tilde{\boldsymbol{S}}_{EM}\widetilde{\boldsymbol{\mathcal{P}}}_{EM}^{\sigma}\widetilde{\boldsymbol{H}}_{EM}^{\sigma}\tilde{\boldsymbol{S}}_{EM}^{-1}+\tilde{\boldsymbol{S}}_{EM}\widetilde{\boldsymbol{\mathcal{P}}}_{EM,R}^{\sigma}\widetilde{\boldsymbol{V}}_{R,EM}^{\sigma}\tilde{\boldsymbol{S}}_{EM}^{-1} & \tilde{\boldsymbol{S}}_{EM}\widetilde{\boldsymbol{\mathcal{P}}}_{EM}^{\sigma}\widetilde{\boldsymbol{V}}_{EM,R}^{\sigma}\boldsymbol{S}_R^{-1}+\tilde{\boldsymbol{S}}_{EM}\widetilde{\boldsymbol{\mathcal{P}}}_{EM,R}^{\sigma}\boldsymbol{H}_R^{\sigma}\boldsymbol{S}_R^{-1}\\ \boldsymbol{S}_R\widetilde{\boldsymbol{\mathcal{P}}}_{R,L}^{\sigma}\boldsymbol{H}_L^{\sigma}\boldsymbol{S}_L^{-1}+\boldsymbol{S}_R\widetilde{\boldsymbol{\mathcal{P}}}_{R,EM}^{\sigma}\widetilde{\boldsymbol{V}}_{EM,L}^{\sigma}\boldsymbol{S}_L^{-1} & \boldsymbol{S}_R\widetilde{\boldsymbol{\mathcal{P}}}_{R,L}^{\sigma}\widetilde{\boldsymbol{V}}_{L,EM}^{\sigma}\tilde{\boldsymbol{S}}_{EM}^{-1}+\boldsymbol{S}_R\widetilde{\boldsymbol{\mathcal{P}}}_{R,EM}^{\sigma}\widetilde{\boldsymbol{H}}_{EM}^{\sigma}\tilde{\boldsymbol{S}}_{EM}^{-1}+\boldsymbol{S}_R\widetilde{\boldsymbol{\mathcal{P}}}_{R}^{\sigma}\widetilde{\boldsymbol{V}}_{R,EM}^{\sigma}\tilde{\boldsymbol{S}}_{EM}^{-1} & \boldsymbol{S}_R\widetilde{\boldsymbol{\mathcal{P}}}_{R,EM}^{\sigma}\widetilde{\boldsymbol{V}}_{EM,R}^{\sigma}\boldsymbol{S}_R^{-1}+\boldsymbol{S}_R\widetilde{\boldsymbol{\mathcal{P}}}_{R}^{\sigma}\boldsymbol{H}_R^{\sigma}\boldsymbol{S}_R^{-1}\end{pmatrix},$$

whose *EM* block is:

$$\left(\tilde{\boldsymbol{\mathcal{S}}}\widetilde{\boldsymbol{\mathcal{P}}}^{\sigma}\widetilde{\boldsymbol{\mathcal{H}}}_{KS}^{\sigma}\tilde{\boldsymbol{\mathcal{S}}}^{-1}\right)_{EM}=\tilde{\boldsymbol{S}}_{EM}\widetilde{\boldsymbol{\mathcal{P}}}_{EM,L}^{\sigma}\widetilde{\boldsymbol{V}}_{L,EM}^{\sigma}\tilde{\boldsymbol{S}}_{EM}^{-1}+\tilde{\boldsymbol{S}}_{EM}\widetilde{\boldsymbol{\mathcal{P}}}_{EM}^{\sigma}\widetilde{\boldsymbol{H}}_{EM}^{\sigma}\tilde{\boldsymbol{S}}_{EM}^{-1}+\tilde{\boldsymbol{S}}_{EM}\widetilde{\boldsymbol{\mathcal{P}}}_{EM,R}^{\sigma}\widetilde{\boldsymbol{V}}_{R,EM}^{\sigma}\tilde{\boldsymbol{S}}_{EM}^{-1}. \tag{S69}$$

The third term reads:

$$\widetilde{\boldsymbol{\mathcal{H}}}_{KS}^{\sigma}\widetilde{\boldsymbol{\mathcal{P}}}^{\sigma}=\begin{pmatrix}\boldsymbol{H}_L^{\sigma} & \widetilde{\boldsymbol{V}}_{L,EM}^{\sigma} & \boldsymbol{0}\\ \widetilde{\boldsymbol{V}}_{EM,L}^{\sigma} & \widetilde{\boldsymbol{H}}_{EM}^{\sigma} & \widetilde{\boldsymbol{V}}_{EM,R}^{\sigma}\\ \boldsymbol{0} & \widetilde{\boldsymbol{V}}_{R,EM}^{\sigma} & \boldsymbol{H}_R^{\sigma}\end{pmatrix}\begin{pmatrix}\widetilde{\boldsymbol{\mathcal{P}}}_{L}^{\sigma} & \widetilde{\boldsymbol{\mathcal{P}}}_{L,EM}^{\sigma} & \widetilde{\boldsymbol{\mathcal{P}}}_{LR}^{\sigma}\\ \widetilde{\boldsymbol{\mathcal{P}}}_{EM,L}^{\sigma} & \widetilde{\boldsymbol{\mathcal{P}}}_{EM}^{\sigma} & \widetilde{\boldsymbol{\mathcal{P}}}_{EM,R}^{\sigma}\\ \widetilde{\boldsymbol{\mathcal{P}}}_{R,L}^{\sigma} & \widetilde{\boldsymbol{\mathcal{P}}}_{R,EM}^{\sigma} & \widetilde{\boldsymbol{\mathcal{P}}}_{R}^{\sigma}\end{pmatrix}= \tag{S70}$$

$$
= \begin{pmatrix} \boldsymbol{H}_L^\sigma \tilde{\boldsymbol{\mathcal{P}}}_L^\sigma + \tilde{\boldsymbol{V}}_{L,EM}^\sigma \tilde{\boldsymbol{\mathcal{P}}}_{EM,L}^\sigma & \boldsymbol{H}_L^\sigma \tilde{\boldsymbol{\mathcal{P}}}_{L,EM}^\sigma + \tilde{\boldsymbol{V}}_{L,EM}^\sigma \tilde{\boldsymbol{\mathcal{P}}}_{EM}^\sigma & \boldsymbol{H}_L^\sigma \tilde{\boldsymbol{\mathcal{P}}}_{LR}^\sigma + \tilde{\boldsymbol{V}}_{L,EM}^\sigma \tilde{\boldsymbol{\mathcal{P}}}_{EM,R}^\sigma \\ \tilde{\boldsymbol{V}}_{EM,L}^\sigma \tilde{\boldsymbol{\mathcal{P}}}_L^\sigma + \tilde{\boldsymbol{H}}_{EM}^\sigma \tilde{\boldsymbol{\mathcal{P}}}_{EM,L}^\sigma + \tilde{\boldsymbol{V}}_{EM,R}^\sigma \tilde{\boldsymbol{\mathcal{P}}}_{R,L}^\sigma & \tilde{\boldsymbol{V}}_{EM,L}^\sigma \tilde{\boldsymbol{\mathcal{P}}}_{L,EM}^\sigma + \tilde{\boldsymbol{H}}_{EM}^\sigma \tilde{\boldsymbol{\mathcal{P}}}_{EM}^\sigma + \tilde{\boldsymbol{V}}_{EM,R}^\sigma \tilde{\boldsymbol{\mathcal{P}}}_{R,EM}^\sigma & \tilde{\boldsymbol{V}}_{EM,L}^\sigma \tilde{\boldsymbol{\mathcal{P}}}_{LR}^\sigma + \tilde{\boldsymbol{H}}_{EM}^\sigma \tilde{\boldsymbol{\mathcal{P}}}_{EM,R}^\sigma + \tilde{\boldsymbol{V}}_{EM,R}^\sigma \tilde{\boldsymbol{\mathcal{P}}}_R^\sigma \\ \tilde{\boldsymbol{V}}_{R,EM}^\sigma \tilde{\boldsymbol{\mathcal{P}}}_{EM,L}^\sigma + \boldsymbol{H}_R^\sigma \tilde{\boldsymbol{\mathcal{P}}}_{R,L}^\sigma & \tilde{\boldsymbol{V}}_{R,EM}^\sigma \tilde{\boldsymbol{\mathcal{P}}}_{EM}^\sigma + \boldsymbol{H}_R^\sigma \tilde{\boldsymbol{\mathcal{P}}}_{R,EM}^\sigma & \tilde{\boldsymbol{V}}_{R,EM}^\sigma \tilde{\boldsymbol{\mathcal{P}}}_{EM,R}^\sigma + \boldsymbol{H}_R^\sigma \tilde{\boldsymbol{\mathcal{P}}}_R^\sigma \end{pmatrix},
$$

whose EM block is:

$$
\left(\tilde{\boldsymbol{\mathcal{H}}}_{KS}^\sigma \tilde{\boldsymbol{\mathcal{P}}}^\sigma\right)_{EM} = \tilde{\boldsymbol{V}}_{EM,L}^\sigma \tilde{\boldsymbol{\mathcal{P}}}_{L,EM}^\sigma + \tilde{\boldsymbol{H}}_{EM}^\sigma \tilde{\boldsymbol{\mathcal{P}}}_{EM}^\sigma + \tilde{\boldsymbol{V}}_{EM,R}^\sigma \tilde{\boldsymbol{\mathcal{P}}}_{R,EM}^\sigma \tag{S71}
$$

The fourth term reads:

$$
\tilde{\boldsymbol{\mathcal{P}}}^\sigma \tilde{\boldsymbol{\mathcal{H}}}_{KS}^\sigma = \begin{pmatrix} \tilde{\boldsymbol{\mathcal{P}}}_L^\sigma & \tilde{\boldsymbol{\mathcal{P}}}_{L,EM}^\sigma & \tilde{\boldsymbol{\mathcal{P}}}_{LR}^\sigma \\ \tilde{\boldsymbol{\mathcal{P}}}_{EM,L}^\sigma & \tilde{\boldsymbol{\mathcal{P}}}_{EM}^\sigma & \tilde{\boldsymbol{\mathcal{P}}}_{EM,R}^\sigma \\ \tilde{\boldsymbol{\mathcal{P}}}_{R,L}^\sigma & \tilde{\boldsymbol{\mathcal{P}}}_{R,EM}^\sigma & \tilde{\boldsymbol{\mathcal{P}}}_R^\sigma \end{pmatrix} \begin{pmatrix} \boldsymbol{H}_L^\sigma & \tilde{\boldsymbol{V}}_{L,EM}^\sigma & \boldsymbol{0} \\ \tilde{\boldsymbol{V}}_{EM,L}^\sigma & \tilde{\boldsymbol{H}}_{EM}^\sigma & \tilde{\boldsymbol{V}}_{EM,R}^\sigma \\ \boldsymbol{0} & \tilde{\boldsymbol{V}}_{R,EM}^\sigma & \boldsymbol{H}_R^\sigma \end{pmatrix} = \tag{S72}
$$

$$
= \begin{pmatrix} \tilde{\boldsymbol{\mathcal{P}}}_L^\sigma \boldsymbol{H}_L^\sigma + \tilde{\boldsymbol{\mathcal{P}}}_{L,EM}^\sigma \tilde{\boldsymbol{V}}_{EM,L}^\sigma & \tilde{\boldsymbol{\mathcal{P}}}_L^\sigma \tilde{\boldsymbol{V}}_{L,EM}^\sigma + \tilde{\boldsymbol{\mathcal{P}}}_{L,EM}^\sigma \tilde{\boldsymbol{H}}_{EM}^\sigma + \tilde{\boldsymbol{\mathcal{P}}}_{LR}^\sigma \tilde{\boldsymbol{V}}_{R,EM}^\sigma & \tilde{\boldsymbol{\mathcal{P}}}_{L,EM}^\sigma \tilde{\boldsymbol{V}}_{EM,R}^\sigma + \tilde{\boldsymbol{\mathcal{P}}}_{LR}^\sigma \boldsymbol{H}_R^\sigma \\ \tilde{\boldsymbol{\mathcal{P}}}_{EM,L}^\sigma \boldsymbol{H}_L^\sigma + \tilde{\boldsymbol{\mathcal{P}}}_{EM}^\sigma \tilde{\boldsymbol{V}}_{EM,L}^\sigma & \tilde{\boldsymbol{\mathcal{P}}}_{EM,L}^\sigma \tilde{\boldsymbol{V}}_{L,EM}^\sigma + \tilde{\boldsymbol{\mathcal{P}}}_{EM}^\sigma \tilde{\boldsymbol{H}}_{EM}^\sigma + \tilde{\boldsymbol{\mathcal{P}}}_{EM,R}^\sigma \tilde{\boldsymbol{V}}_{R,EM}^\sigma & \tilde{\boldsymbol{\mathcal{P}}}_{EM}^\sigma \tilde{\boldsymbol{V}}_{EM,R}^\sigma + \tilde{\boldsymbol{\mathcal{P}}}_{EM,R}^\sigma \boldsymbol{H}_R^\sigma \\ \tilde{\boldsymbol{\mathcal{P}}}_{R,L}^\sigma \boldsymbol{H}_L^\sigma + \tilde{\boldsymbol{\mathcal{P}}}_{R,EM}^\sigma \tilde{\boldsymbol{V}}_{EM,L}^\sigma & \tilde{\boldsymbol{\mathcal{P}}}_{R,L}^\sigma \tilde{\boldsymbol{V}}_{L,EM}^\sigma + \tilde{\boldsymbol{\mathcal{P}}}_{R,EM}^\sigma \tilde{\boldsymbol{H}}_{EM}^\sigma + \tilde{\boldsymbol{\mathcal{P}}}_R^\sigma \tilde{\boldsymbol{V}}_{R,EM}^\sigma & \tilde{\boldsymbol{\mathcal{P}}}_{R,EM}^\sigma \tilde{\boldsymbol{V}}_{EM,R}^\sigma + \tilde{\boldsymbol{\mathcal{P}}}_R^\sigma \boldsymbol{H}_R^\sigma \end{pmatrix},
$$

whose EM block is:

$$
\left(\tilde{\boldsymbol{\mathcal{P}}}^\sigma \tilde{\boldsymbol{\mathcal{H}}}_{KS}^\sigma\right)_{EM} = \tilde{\boldsymbol{\mathcal{P}}}_{EM,L}^\sigma \tilde{\boldsymbol{V}}_{L,EM}^\sigma + \tilde{\boldsymbol{\mathcal{P}}}_{EM}^\sigma \tilde{\boldsymbol{H}}_{EM}^\sigma + \tilde{\boldsymbol{\mathcal{P}}}_{EM,R}^\sigma \tilde{\boldsymbol{V}}_{R,EM}^\sigma \tag{S73}
$$

Collecting all terms in Eqs. (S67), (S69), (S71), and (S73) we may write the right-hand side of Eq. (S65) as follows:

$$
\begin{aligned}
&-\frac{i}{2} tr_{EM}\left[\tilde{\boldsymbol{\mathcal{S}}}^{-1} \tilde{\boldsymbol{\mathcal{H}}}_{KS}^\sigma \tilde{\boldsymbol{\mathcal{P}}}^\sigma \tilde{\boldsymbol{\mathcal{S}}} - \tilde{\boldsymbol{\mathcal{S}}} \tilde{\boldsymbol{\mathcal{P}}}^\sigma \tilde{\boldsymbol{\mathcal{H}}}_{KS}^\sigma \tilde{\boldsymbol{\mathcal{S}}}^{-1} + \tilde{\boldsymbol{\mathcal{H}}}_{KS}^\sigma \tilde{\boldsymbol{\mathcal{P}}}^\sigma - \tilde{\boldsymbol{\mathcal{P}}}^\sigma \tilde{\boldsymbol{\mathcal{H}}}_{KS}^\sigma\right] = \\
&= -\tfrac{i}{2} tr_{EM}[\tilde{\boldsymbol{S}}_{EM}^{-1} \tilde{\boldsymbol{V}}_{EM,L}^\sigma \tilde{\boldsymbol{\mathcal{P}}}_{L,EM}^\sigma \tilde{\boldsymbol{S}}_{EM} + \tilde{\boldsymbol{S}}_{EM}^{-1} \boldsymbol{H}_{EM}^\sigma \tilde{\boldsymbol{\mathcal{P}}}_{EM}^\sigma \tilde{\boldsymbol{S}}_{EM} + \tilde{\boldsymbol{S}}_{EM}^{-1} \tilde{\boldsymbol{V}}_{EM,L}^\sigma \tilde{\boldsymbol{\mathcal{P}}}_{R,EM}^\sigma \tilde{\boldsymbol{S}}_{EM} - \\
&- \tilde{\boldsymbol{S}}_{EM} \tilde{\boldsymbol{\mathcal{P}}}_{EM,L}^\sigma \tilde{\boldsymbol{V}}_{L,EM}^\sigma \tilde{\boldsymbol{S}}_{EM}^{-1} - \tilde{\boldsymbol{S}}_{EM} \tilde{\boldsymbol{\mathcal{P}}}_{EM}^\sigma \tilde{\boldsymbol{H}}_{EM}^\sigma \tilde{\boldsymbol{S}}_{EM}^{-1} - \tilde{\boldsymbol{S}}_{EM} \tilde{\boldsymbol{\mathcal{P}}}_{EM,R}^\sigma \tilde{\boldsymbol{V}}_{R,EM}^\sigma \tilde{\boldsymbol{S}}_{EM}^{-1} + \\
&+ \tilde{\boldsymbol{V}}_{EM,L}^\sigma \tilde{\boldsymbol{\mathcal{P}}}_{L,EM}^\sigma + \tilde{\boldsymbol{H}}_{EM}^\sigma \tilde{\boldsymbol{\mathcal{P}}}_{EM}^\sigma + \tilde{\boldsymbol{V}}_{EM,R}^\sigma \tilde{\boldsymbol{\mathcal{P}}}_{R,EM}^\sigma - \tilde{\boldsymbol{\mathcal{P}}}_{EM,L}^\sigma \tilde{\boldsymbol{V}}_{L,EM}^\sigma - \tilde{\boldsymbol{\mathcal{P}}}_{EM}^\sigma \tilde{\boldsymbol{H}}_{EM}^\sigma - \tilde{\boldsymbol{\mathcal{P}}}_{EM,R}^\sigma \tilde{\boldsymbol{V}}_{R,EM}^\sigma] =
\end{aligned} \tag{S74}
$$

This can be reordered as follows:

$$
\begin{aligned}
&= -\frac{i}{2} tr_{EM}\left(\tilde{\boldsymbol{S}}_{EM}^{-1} \tilde{\boldsymbol{H}}_{EM}^\sigma \tilde{\boldsymbol{\mathcal{P}}}_{EM}^\sigma \tilde{\boldsymbol{S}}_{EM} - \tilde{\boldsymbol{S}}_{EM} \tilde{\boldsymbol{\mathcal{P}}}_{EM}^\sigma \tilde{\boldsymbol{H}}_{EM}^\sigma \tilde{\boldsymbol{S}}_{EM}^{-1} + \tilde{\boldsymbol{H}}_{EM}^\sigma \tilde{\boldsymbol{\mathcal{P}}}_{EM}^\sigma - \tilde{\boldsymbol{\mathcal{P}}}_{EM}^\sigma \tilde{\boldsymbol{H}}_{EM}^\sigma\right) \\
&- \tfrac{i}{2} tr_{EM}\left(\tilde{\boldsymbol{S}}_{EM}^{-1} \tilde{\boldsymbol{V}}_{EM,L}^\sigma \tilde{\boldsymbol{\mathcal{P}}}_{L,EM}^\sigma \tilde{\boldsymbol{S}}_{EM} - \tilde{\boldsymbol{S}}_{EM} \tilde{\boldsymbol{\mathcal{P}}}_{EM,L}^\sigma \tilde{\boldsymbol{V}}_{L,EM}^\sigma \tilde{\boldsymbol{S}}_{EM}^{-1} + \tilde{\boldsymbol{V}}_{EM,L}^\sigma \tilde{\boldsymbol{\mathcal{P}}}_{L,EM}^\sigma - \tilde{\boldsymbol{\mathcal{P}}}_{EM,L}^\sigma \tilde{\boldsymbol{V}}_{L,EM}^\sigma\right) - \\
&- \tfrac{i}{2} tr_{EM}\left(\tilde{\boldsymbol{S}}_{EM}^{-1} \tilde{\boldsymbol{V}}_{EM,R}^\sigma \tilde{\boldsymbol{\mathcal{P}}}_{R,EM}^\sigma \tilde{\boldsymbol{S}}_{EM} - \tilde{\boldsymbol{S}}_{EM} \tilde{\boldsymbol{\mathcal{P}}}_{EM,R}^\sigma \tilde{\boldsymbol{V}}_{R,EM}^\sigma \tilde{\boldsymbol{S}}_{EM}^{-1} + \tilde{\boldsymbol{V}}_{EM,R}^\sigma \tilde{\boldsymbol{\mathcal{P}}}_{R,EM}^\sigma - \tilde{\boldsymbol{\mathcal{P}}}_{EM,R}^\sigma \tilde{\boldsymbol{V}}_{R,EM}^\sigma\right).
\end{aligned} \tag{S75}
$$

In the first row of Eq. (S75), the partial EM trace obeys the cyclic property, since all the matrices involved are square matrices of dimension EM. Therefore, the contribution of this term vanishes:

$$
\begin{aligned}
&tr_{EM}\left(\tilde{\boldsymbol{S}}_{EM}^{-1} \tilde{\boldsymbol{H}}_{EM}^\sigma \tilde{\boldsymbol{\mathcal{P}}}_{EM}^\sigma \tilde{\boldsymbol{S}}_{EM} - \tilde{\boldsymbol{S}}_{EM} \tilde{\boldsymbol{\mathcal{P}}}_{EM}^\sigma \tilde{\boldsymbol{H}}_{EM}^\sigma \tilde{\boldsymbol{S}}_{EM}^{-1} + \tilde{\boldsymbol{H}}_{EM}^\sigma \tilde{\boldsymbol{\mathcal{P}}}_{EM}^\sigma - \tilde{\boldsymbol{\mathcal{P}}}_{EM}^\sigma \tilde{\boldsymbol{H}}_{EM}^\sigma\right) = \\
&= tr_{EM}\left(\tilde{\boldsymbol{H}}_{EM}^\sigma \tilde{\boldsymbol{\mathcal{P}}}_{EM}^\sigma \tilde{\boldsymbol{S}}_{EM} \tilde{\boldsymbol{S}}_{EM}^{-1} - \tilde{\boldsymbol{\mathcal{P}}}_{EM}^\sigma \tilde{\boldsymbol{H}}_{EM}^\sigma \tilde{\boldsymbol{S}}_{EM}^{-1} \tilde{\boldsymbol{S}}_{EM} + \tilde{\boldsymbol{H}}_{EM}^\sigma \tilde{\boldsymbol{\mathcal{P}}}_{EM}^\sigma - \tilde{\boldsymbol{\mathcal{P}}}_{EM}^\sigma \tilde{\boldsymbol{H}}_{EM}^\sigma\right) = \\
&= tr_{EM}\left(\tilde{\boldsymbol{H}}_{EM}^\sigma \tilde{\boldsymbol{\mathcal{P}}}_{EM}^\sigma - \tilde{\boldsymbol{\mathcal{P}}}_{EM}^\sigma \tilde{\boldsymbol{H}}_{EM}^\sigma + \tilde{\boldsymbol{H}}_{EM}^\sigma \tilde{\boldsymbol{\mathcal{P}}}_{EM}^\sigma - \tilde{\boldsymbol{\mathcal{P}}}_{EM}^\sigma \tilde{\boldsymbol{H}}_{EM}^\sigma\right) = \\
&= 2tr_{EM}\left(\tilde{\boldsymbol{H}}_{EM}^\sigma \tilde{\boldsymbol{\mathcal{P}}}_{EM}^\sigma - \tilde{\boldsymbol{\mathcal{P}}}_{EM}^\sigma \tilde{\boldsymbol{H}}_{EM}^\sigma\right) = 2tr_{EM}\left(\tilde{\boldsymbol{\mathcal{P}}}_{EM}^\sigma \tilde{\boldsymbol{H}}_{EM}^\sigma - \tilde{\boldsymbol{\mathcal{P}}}_{EM}^\sigma \tilde{\boldsymbol{H}}_{EM}^\sigma\right) = 0.
\end{aligned} \tag{S76}
$$

Looking next into the first term on the second row of Eq. (S75) we can write:

$$tr_{EM}\left(\tilde{\boldsymbol{S}}_{EM}^{-1}\widetilde{\boldsymbol{V}}_{EM,L}^{\sigma}\widetilde{\boldsymbol{\mathcal{P}}}_{L,EM}^{\sigma}\tilde{\boldsymbol{S}}_{EM}\right) = \sum_{i\in EM}\sum_{j\in EM}\sum_{k\in L}\sum_{l\in EM}\left(\tilde{\boldsymbol{S}}_{EM}^{-1}\right)_{ij}\left(\widetilde{\boldsymbol{V}}_{EM,L}^{\sigma}\right)_{jk}\left(\widetilde{\boldsymbol{\mathcal{P}}}_{L,EM}^{\sigma}\right)_{kl}\left(\tilde{\boldsymbol{S}}_{EM}\right)_{li} =$$
$$= \sum_{j\in EM}\sum_{k\in L}\sum_{l\in EM}\sum_{i\in EM}\left(\widetilde{\boldsymbol{V}}_{EM,L}^{\sigma}\right)_{jk}\left(\widetilde{\boldsymbol{\mathcal{P}}}_{L,EM}^{\sigma}\right)_{kl}\left(\tilde{\boldsymbol{S}}_{EM}\right)_{li}\left(\tilde{\boldsymbol{S}}_{EM}^{-1}\right)_{ij} = \tag{S77}$$
$$= tr_{EM}\left(\widetilde{\boldsymbol{V}}_{EM,L}^{\sigma}\widetilde{\boldsymbol{\mathcal{P}}}_{L,EM}^{\sigma}\tilde{\boldsymbol{S}}_{EM}\tilde{\boldsymbol{S}}_{EM}^{-1}\right) = tr_{EM}\left(\widetilde{\boldsymbol{V}}_{EM,L}^{\sigma}\widetilde{\boldsymbol{\mathcal{P}}}_{L,EM}^{\sigma}\right),$$

where in the second row we switched the summation order and changed the orders of the summed elements. Similarly, for the second term on the second row of Eq. (S75) we have:

$$tr_{EM}\left(\tilde{\boldsymbol{S}}_{EM}\widetilde{\boldsymbol{\mathcal{P}}}_{EM,L}^{\sigma}\widetilde{\boldsymbol{V}}_{L,EM}^{\sigma}\tilde{\boldsymbol{S}}_{EM}^{-1}\right) = tr_{EM}\left(\widetilde{\boldsymbol{\mathcal{P}}}_{EM,L}^{\sigma}\widetilde{\boldsymbol{V}}_{L,EM}^{\sigma}\right), \tag{S78}$$

and for the two first terms in the third row:

$$tr_{EM}\left(\tilde{\boldsymbol{S}}_{EM}^{-1}\widetilde{\boldsymbol{V}}_{EM,R}^{\sigma}\widetilde{\boldsymbol{\mathcal{P}}}_{R,EM}^{\sigma}\tilde{\boldsymbol{S}}_{EM} - \tilde{\boldsymbol{S}}_{EM}\widetilde{\boldsymbol{\mathcal{P}}}_{EM,R}^{\sigma}\widetilde{\boldsymbol{V}}_{R,EM}^{\sigma}\tilde{\boldsymbol{S}}_{EM}^{-1}\right) = tr_{EM}\left(\widetilde{\boldsymbol{V}}_{EM,R}^{\sigma}\widetilde{\boldsymbol{\mathcal{P}}}_{R,EM}^{\sigma} - \widetilde{\boldsymbol{\mathcal{P}}}_{EM,R}^{\sigma}\widetilde{\boldsymbol{V}}_{R,EM}^{\sigma}\right). \tag{S79}$$

Collecting all terms in Eqs. (S75) - (S79) we have:

$$\dot{N}_{EM}^{\sigma} = -i\cdot tr_{EM}\left(\widetilde{\boldsymbol{V}}_{EM,L}^{\sigma}\widetilde{\boldsymbol{\mathcal{P}}}_{L,EM}^{\sigma} - \widetilde{\boldsymbol{\mathcal{P}}}_{EM,L}^{\sigma}\widetilde{\boldsymbol{V}}_{L,EM}^{\sigma}\right) - i\cdot tr_{EM}\left(\widetilde{\boldsymbol{V}}_{EM,R}^{\sigma}\widetilde{\boldsymbol{\mathcal{P}}}_{R,EM}^{\sigma} - \widetilde{\boldsymbol{\mathcal{P}}}_{EM,R}^{\sigma}\widetilde{\boldsymbol{V}}_{R,EM}^{\sigma}\right). \tag{S80}$$

Using the fact that the density matrix and KS Hamiltonian matrix are Hermitian we can further write:

$$\left[tr_{EM}\left(\widetilde{\boldsymbol{V}}_{EM,L}^{\sigma}\widetilde{\boldsymbol{\mathcal{P}}}_{L,EM}^{\sigma}\right)\right]^{*} = \left[\sum_{i\in EM}\sum_{j\in L}\left(\widetilde{\boldsymbol{V}}_{EM,L}^{\sigma}\right)_{ij}\left(\widetilde{\boldsymbol{\mathcal{P}}}_{L,EM}^{\sigma}\right)_{ji}\right]^{*} = \sum_{i\in EM}\sum_{j\in L}\left(\widetilde{\boldsymbol{V}}_{EM,L}^{\sigma}\right)_{ij}^{*}\left(\widetilde{\boldsymbol{\mathcal{P}}}_{L,EM}^{\sigma}\right)_{ji}^{*} =$$
$$= \sum_{i\in EM}\sum_{j\in L}\left(\widetilde{\boldsymbol{V}}_{L,EM}^{\sigma}\right)_{ji}\left(\widetilde{\boldsymbol{\mathcal{P}}}_{EM,L}^{\sigma}\right)_{ij} = \sum_{i\in EM}\sum_{j\in L}\left(\widetilde{\boldsymbol{\mathcal{P}}}_{EM,L}^{\sigma}\right)_{ij}\left(\widetilde{\boldsymbol{V}}_{L,EM}^{\sigma}\right)_{ji} = tr_{EM}\left(\widetilde{\boldsymbol{\mathcal{P}}}_{EM,L}^{\sigma}\widetilde{\boldsymbol{V}}_{L,EM}^{\sigma}\right). \tag{S81}$$

Similarly, we can write $\left[tr_{EM}\left(\widetilde{\boldsymbol{V}}_{EM,R}^{\sigma}\widetilde{\boldsymbol{\mathcal{P}}}_{R,EM}^{\sigma}\right)\right]^{*} = tr_{EM}\left(\widetilde{\boldsymbol{\mathcal{P}}}_{EM,R}^{\sigma}\widetilde{\boldsymbol{V}}_{R,EM}^{\sigma}\right)$, so that:

$$\dot{N}_{EM}^{\sigma} = -i\cdot tr_{EM}\left(\widetilde{\boldsymbol{V}}_{EM,L}^{\sigma}\widetilde{\boldsymbol{\mathcal{P}}}_{L,EM}^{\sigma} - \widetilde{\boldsymbol{\mathcal{P}}}_{EM,L}^{\sigma}\widetilde{\boldsymbol{V}}_{L,EM}^{\sigma}\right) - i\cdot tr_{EM}\left(\widetilde{\boldsymbol{V}}_{EM,R}^{\sigma}\widetilde{\boldsymbol{\mathcal{P}}}_{R,EM}^{\sigma} - \widetilde{\boldsymbol{\mathcal{P}}}_{EM,R}^{\sigma}\widetilde{\boldsymbol{V}}_{R,EM}^{\sigma}\right) =$$
$$= -i\cdot tr_{EM}\left({\widetilde{\boldsymbol{\mathcal{P}}}_{EM,L}^{\sigma}}^{*}{\widetilde{\boldsymbol{V}}_{L,EM}^{\sigma}}^{*} - \widetilde{\boldsymbol{\mathcal{P}}}_{EM,L}^{\sigma}\widetilde{\boldsymbol{V}}_{L,EM}^{\sigma}\right) - i\cdot tr_{EM}\left({\widetilde{\boldsymbol{\mathcal{P}}}_{EM,R}^{\sigma}}^{*}{\widetilde{\boldsymbol{V}}_{R,EM}^{\sigma}}^{*} - \widetilde{\boldsymbol{\mathcal{P}}}_{EM,R}^{\sigma}\widetilde{\boldsymbol{V}}_{R,EM}^{\sigma}\right) =$$
$$= -i\cdot tr_{EM}\left[2i\cdot Im\left(\widetilde{\boldsymbol{\mathcal{P}}}_{EM,L}^{\sigma}\widetilde{\boldsymbol{V}}_{L,EM}^{\sigma}\right)\right] - i\cdot tr_{EM}\left[2i\cdot Im\left(\widetilde{\boldsymbol{\mathcal{P}}}_{EM,R}^{\sigma}\widetilde{\boldsymbol{V}}_{R,EM}^{\sigma}\right)\right] = \tag{S82}$$
$$= 2\cdot tr_{EM}\left[Im\left(\widetilde{\boldsymbol{\mathcal{P}}}_{EM,L}^{\sigma}\widetilde{\boldsymbol{V}}_{L,EM}^{\sigma}\right)\right] + 2\cdot tr_{EM}\left[Im\left(\widetilde{\boldsymbol{\mathcal{P}}}_{EM,R}^{\sigma}\widetilde{\boldsymbol{V}}_{R,EM}^{\sigma}\right)\right] =$$
$$= 2\cdot Im\left[tr_{EM}\left(\widetilde{\boldsymbol{\mathcal{P}}}_{EM,L}^{\sigma}\widetilde{\boldsymbol{V}}_{L,EM}^{\sigma}\right)\right] + 2\cdot Im\left[tr_{EM}\left(\widetilde{\boldsymbol{\mathcal{P}}}_{EM,R}^{\sigma}\widetilde{\boldsymbol{V}}_{R,EM}^{\sigma}\right)\right].$$

We can thus identify the first term in the last line of Eq. (S82) as the current flowing from the L driven lead into the EM section and the second term as the current flowing from the R driven lead into the EM section:

$$J_{L\to EM}^{\sigma} = 2\cdot Im\left[tr_{EM}\left(\widetilde{\boldsymbol{\mathcal{P}}}_{EM,L}^{\sigma}\widetilde{\boldsymbol{V}}_{L,EM}^{\sigma}\right)\right], \tag{S83}$$

and

$$J_{R\to EM}^{\sigma} = 2\cdot Im\left[tr_{EM}\left(\widetilde{\boldsymbol{\mathcal{P}}}_{EM,R}^{\sigma}\widetilde{\boldsymbol{V}}_{R,EM}^{\sigma}\right)\right]. \tag{S84}$$

Accordingly, the instantaneous average total current flowing through the EM section at time t is:

$$J^{\sigma}(t) = 0.5\big(J^{\sigma}_{L\to EM}(t) + J^{\sigma}_{EM\to R}(t)\big) = Im\big\{tr_{EM}\big[\widetilde{\boldsymbol{\mathcal{P}}}^{\sigma}_{EM,L}(t)\widetilde{\boldsymbol{V}}^{\sigma}_{L,EM}(t)\big]\big\} - Im\big\{tr_{EM}\big[\widetilde{\boldsymbol{\mathcal{P}}}^{\sigma}_{EM,R}(t)\widetilde{\boldsymbol{V}}^{\sigma}_{R,EM}(t)\big]\big\}$$

$$= Im\big\{tr_{EM}\big[\widetilde{\boldsymbol{\mathcal{P}}}^{\sigma}_{EM,L}(t)\widetilde{\boldsymbol{V}}^{\sigma}_{L,EM}(t) - \widetilde{\boldsymbol{\mathcal{P}}}^{\sigma}_{EM,R}(t)\widetilde{\boldsymbol{V}}^{\sigma}_{R,EM}(t)\big]\big\}, \quad \text{(S85)}$$

which is the final expression that we use for the evaluation of the current in the block-diagonal representation.

4. Current dynamics for the H-He-H junction with anti-ferromagnetic spin configuration

In main text Fig. 4 we have provided current traces for the asymmetrically-biased H-He-H bridged junction, at the ferromagnetic state. For completeness, we provide below the spin-resolved current traces (Fig. S4) and the spatially- and spin-resolved steady-state current density heat-maps (Fig. S5) for the anti-ferromagnetic configuration calculated at the same level of theory.

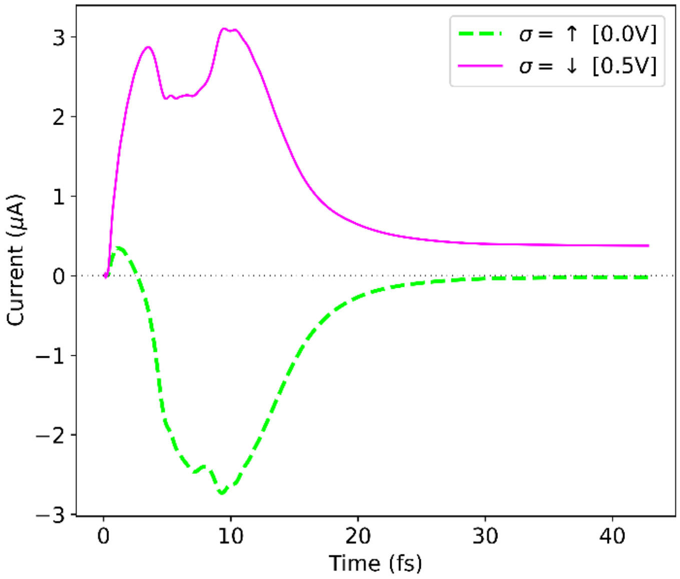

Figure S4: Spin-resolved current traces obtained from the DLvN-TDDFT dynamics of a molecular junction model consisting of two hydrogen chain leads bridged by a perpendicular H-He-H magnetic molecule starting from the antiferromagnetic configuration (Fig. 1(b) of the main text). A bias voltage of 0.5V is applied to the ↓-spin channel while the other channel remains unbiased.

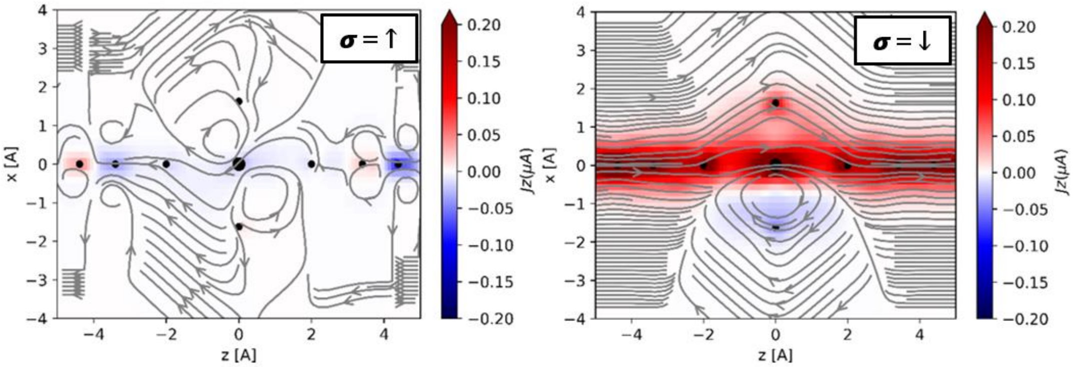

Figure S5: Spatially resolved heatmaps of the ↑-spin (left) and ↓-spin (right) steady-state spin-resolved current densities calculated for the H-He-H based junction (Fig. 1(b) of the main text) at the antiferromagnetic configuration with only the ↓-spin channel biased at 0.5 V.

5. Sensitivity of the transient dynamics of the H-He-H bridged molecular junction towards the driving rate.

In SI section 2 we have provided a density of states analysis justifying the choice of driving rate for the different model systems considered. To evaluate the sensitivity of the calculated current traces towards the choice of driving rate, we performed additional comparative DLvN-TDDFT simulations for the H-He-H based molecular junction over a wide range of driving rates. The current traces for both spin channels, starting from the ferromagnetic configuration, are shown in Figure S6. The demonstrated weak dependence of the current dynamics on the driving rate supports the robustness of our results.

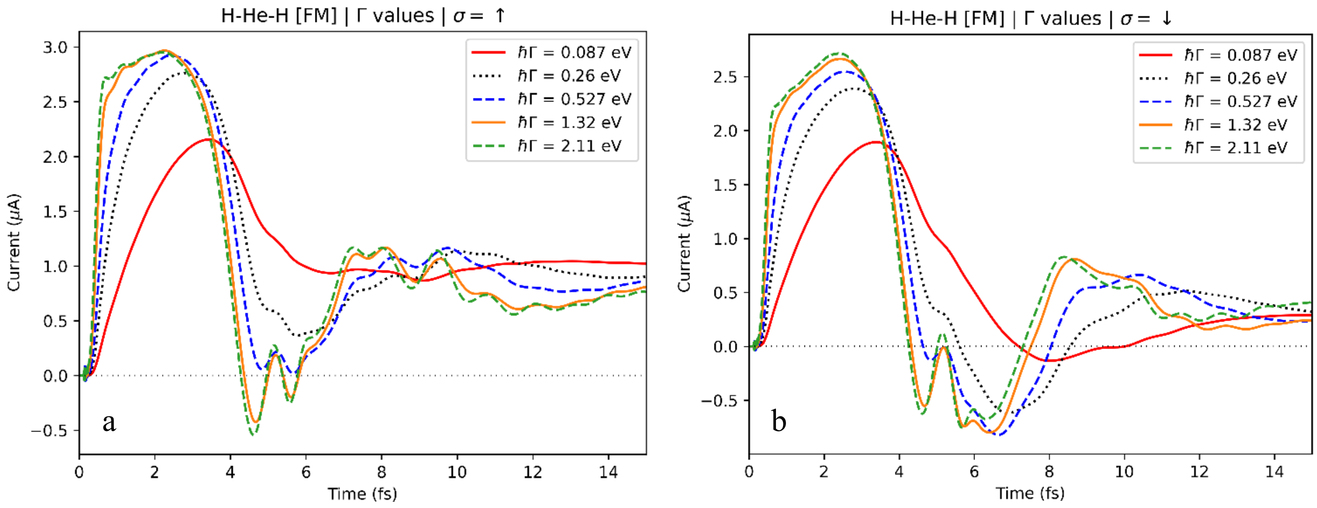

Figure S6: Current traces for (a) ↑-spin and (b) ↓-spin obtained from the DLvN-TDDFT dynamics for various driving rates (Γ) in a molecular junction model consisting of two hydrogen chain leads bridged by a perpendicular H-He-H magnetic molecule starting from the ferromagnetic configuration (Fig. 1(b) of the main text). A bias voltage of 0.5 V is applied to both spin channels. All other run parameters are the same as in main text Figure 3(b).

6. Sensitivity of the transient dynamics of the H-He-H bridged molecular junction towards the driving term switch-on procedure.

To ensure that all quantities (e.g. the density matrix) vary smoothly during the dynamics, the driving rate, Γ, was gradually increased from zero to its full value, Γ_f, using a hyperbolic tangent function of the following form:

$$\Gamma(t) = \frac{1}{2}\left(\tanh\left(\frac{t-t0}{w}\right) + 1\right)\Gamma_f,$$

where t is the time, t_0 = 0.36 fs is the half-rise time, and w = 0.096 fs is the ramp width.

To evaluate the sensitivity of the current trace towards the driving rate ramping procedure, we compare in Figure S7 the time-dependent spin-resolved current traces calculated for the H-He-H based molecular junction model under a bias voltage of 0.5 V, for the cases where Γ is switched on abruptly at $t = 0$ (step function) and gradually (ramp) to its full value with the above mentioned parameters. The abrupt switching-on results in minor high-frequency oscilations in the current during the early-stages of the dynamics and thus it is numerically advantageous to switch on the driving rate gradually. Nonetheless, the overall current dynamics is hardly affected by the driving rate ramping procedure, thus supporting the robustness of our results.

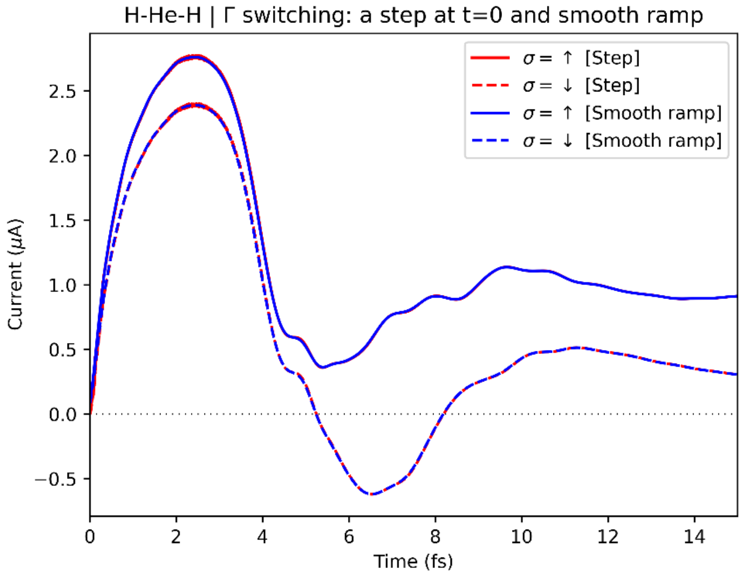

Figure S7: Spin-resolved current traces obtained from the DLvN-TDDFT dynamics for abrupt (step, red lines) and gradual (ramp, blue lines) switching-on the driving rate (Γ) in a molecular junction model consisting of two hydrogen chain leads bridged by a perpendicular H-He-H magnetic molecule, starting from the ferromagnetic configuration (Fig. 1(b) of the main text). A bias voltage of 0.5 V is applied to both spin channels. All other run parameters are the same as in main text Figure 3(b). Note that the current trace of the gradual Γ ramping simulation was shifted to the left to make it coinside with the current trace of the abrupt switch-on couterpart.

7. Effect of the explicit lead model dimensions on the transient current traces of the H-He-H bridged molecular junction

To evaluate the convergence of our results with respect to the choice of explicit lead model dimensions, we present in Figure S8 results of comparative simulations performed for the ferromagnetic H-He-H based molecular junctions with hydrogen chain lead dimensions of 200, 300 (same as in the main text), and 400 hydrogen atoms. For each lead size, the driving rate was obtained by fitting the Lorentizan broadened density of states of the finite lead model to that of the corresponding infinite lead. The results clearly demonstrate weak dependence of the current traces on the finite lead model dimensions with the same qualitative features and only minor quantitative differences.

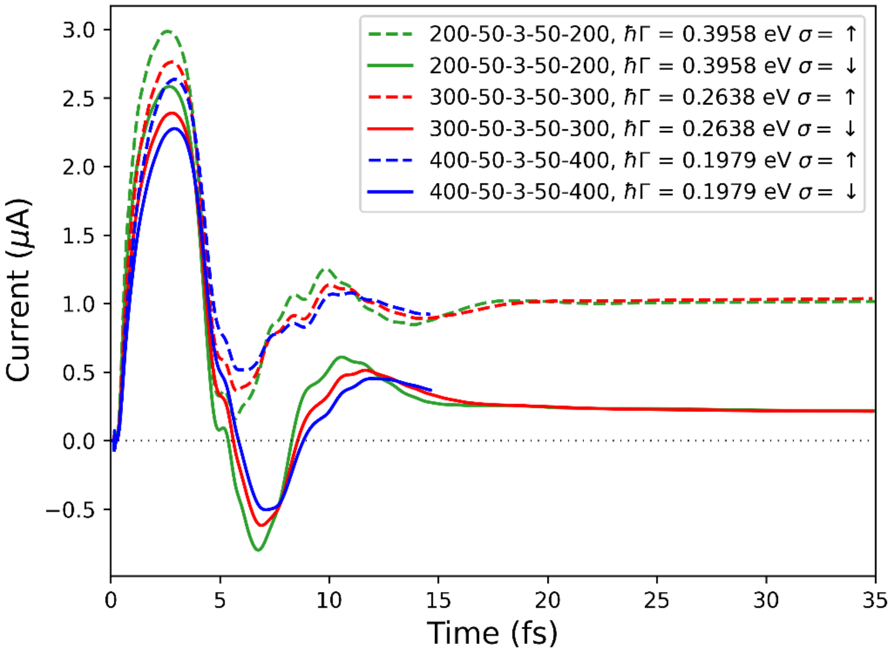

Figure S8: Spin-resolved current traces obtained for a molecular junction model composed of two hydrogen-chain leads, bridged by a perpendicular H-He-H ferromagnetic molecule. Hydrogen-chain leads of lengths 200, 300, and 400 atoms were used with driving rates of 0.3958 eV, 0.2638 eV, and 0.1979 eV, respectively, obtained using DOS fitting, as explained in Section 2 above. The dynamics was performed under a bias voltage of 0.5 V applied to both spin channels, starting from the ferromagnetic configuration shown in Fig. 1(b) of the main text. All other simulation parameters match those used in Fig. 3(b) of the main text.

8. Sensitivity of the transient dynamics of the H-He-H bridged molecular junction towards the convergence tolerance used in the implicit Euler propagator

In the present study, the integration of the DLvN equation of motion is performed using the implicit Euler propagator. In our implementation we use the norm of the density matrix variation as the convergence parameter for the interactive process. To validate our choice of convergence criterion we have tightened it from 10^{-5} (used for all the calculations in the main text) to 10^{-7}. As can be seen in Figure S9, the comparison of the current traces with these two different settings demonstrates full convergence of our results with respect to this parameter.

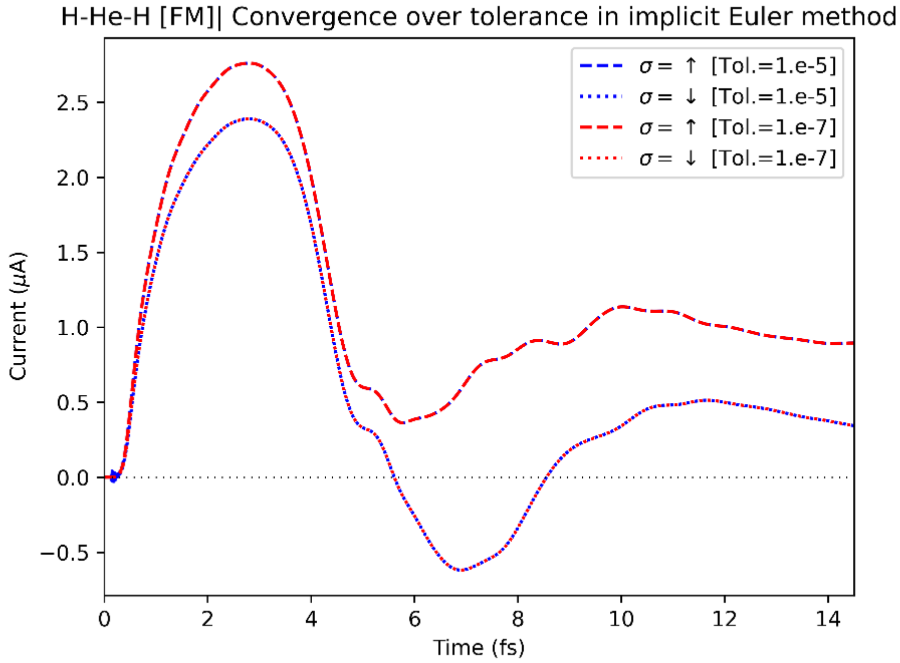

Figure S9: Spin resolved current traces obtained from the DLvN-TDDFT dynamics of a molecular junction model consisting of two hydrogen chain leads bridged by a perpendicular H-He-H magnetic molecule for two different convergence criteria of the implicit Euler propagator. The dynamics is performed under a bias voltage of 0.5 V, applied to the both spin channels, starting from the ferromagnetic configuration (Fig. 1(b) of the main text). All other run parameters are the same as in main text Figure 3(b).

For completeness we report the typical time-step data of the implicit Euler DLvN propagation for the H-He-H based molecular junction presented in the main text to be: minimal time-step - 2.41×10^{-4} fs; mean time-step - 4.24×10^{-3} fs; median time-step - 4.08×10^{-3} fs; maximal time-step - 6.92×10^{-3} fs.

9. Estimation of the effect of ghost currents

Due to the partitioning scheme implemented in the DLvN-TDDFT methodology the obtained current traces are in jeopardy of being contaminated by ghost currents.[7,8]To assess the level of this contamination, we performed dynamical simulations of the hydrogen chain junction model, while replacing the atoms in the extended molecule by their corresponding ghost atoms (namely, removing all the electrons and nuclear changes while keeping only the basis functions; see Figure S10). These calculations were performed for different basis sets (STO-3G, 3-21G, 6-31G (d, p)) applied uniformly to the entire junction mode, as well as the mixed basis set used in the main text. The results, presented in Figures S11 and S12, demonstrate that the contribution of ghost currents is significantly smaller than the physical current for all cases considered. For the mixed basis set the ghost current contribution is of the order of ~0.01 μA (Figure S11a), compared to the physical current of 4.4 μA (see Figure S12). For the uniform basis sets, even lower ghost currents of the order of $0.001 - 0.003$ μA were recorded (see Figure S11b). We attribute these relatively small values of the ghost current both to the large size of the extended molecule section used to describe the molecular junction (21.57 Å in length, see Figure 1a in the main text), and to the intermediate block orthogonalization step that we implement as part of the DLvN scheme. Altogether, we conclude that ghost current contamination of our results is negligible.

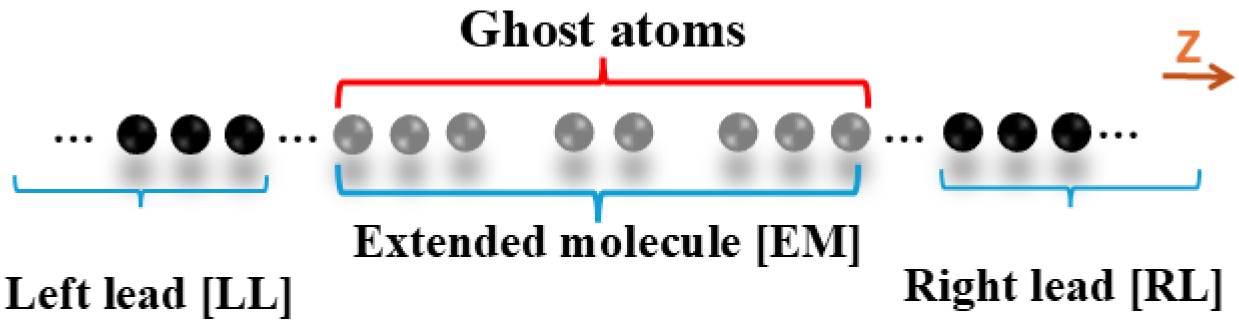

Figure S10: A weakly coupled H-H molecule sandwitched between the two uniform hydrogen chain lead models as described in Figure 1(a) of the main text. In this section, the hydrogen atoms in the extended molecule section are treated as ghost atoms.

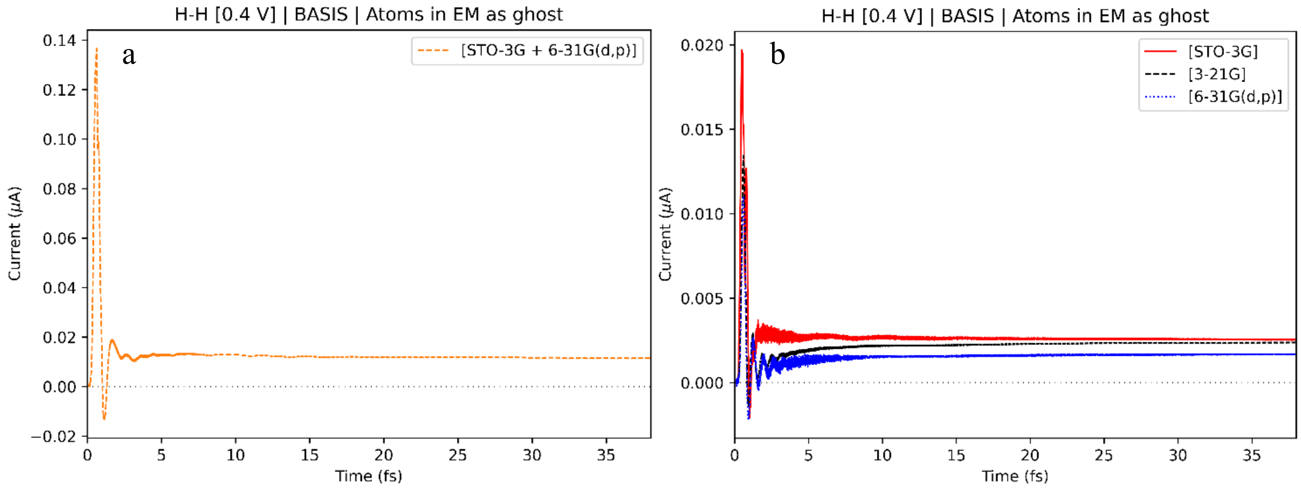

(a) (b)

Figure S11: DLvN-TDDFT current traces obtained for the hydrogen chain molecular junctions with the atoms in the extended molecul section treated as ghost atoms with (a) a mixed basis set of STO-3G (leads)/6-31G(d,p) (extended molecule); and (b) different uniform basis sets of STO-3G, 3-21G, 6-31G(d,p) used across the entire junction model. All run parameters are the same as in main text Figure 2.

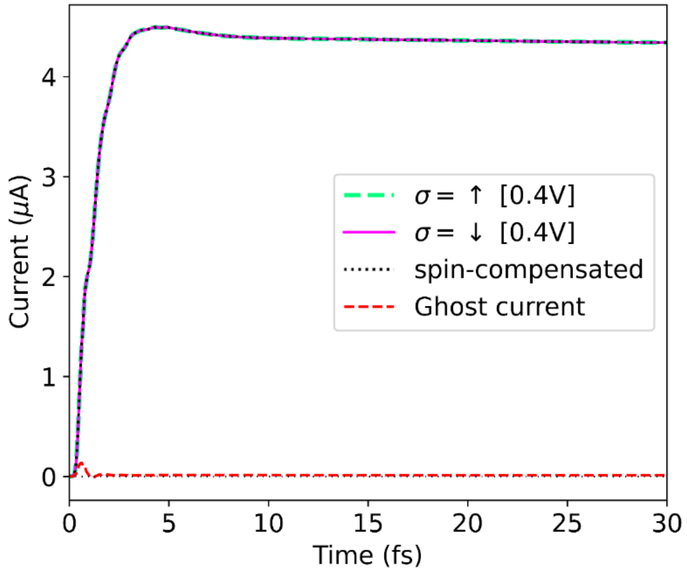

Figure S12: DLvN-TDDFT physical current traces obtained for the hydrogen chain molecular junction with the mixed basis set (STO-3G on the lead models and 6-31G(d,p) on the extended molecule) for the spin compensated (dotted black line) and spin polariozed (purple and dashed green lines) cases, as compared to the results obtained for the ghosted extended molecule section with the same basis set (dashed red line). All run parameters are the same as in main text Figure 2.

10. Determination of the electric field strength for the zigzag GNR calculation

In the main text, we applied an in-plane electric field transverse to the zigzag graphene nanoribbon (GNR) main axis to manipulate the spin current of the junction. To determine the strength of electric field that is required to turn the corresponding finite zigzag GNR (see Fig. S13) half-metallic[9-10], we plot its HOMO-LUMO spin-dependent gap as a function of electric field strength in Fig. S14. A field strength of 0.617 V/Å is adopted to obtain the results presented in main text Fig. 5, for which the ↓-spin gap nearly vanishes, whereas for the ↑-spins the gap is ~0.9 eV.

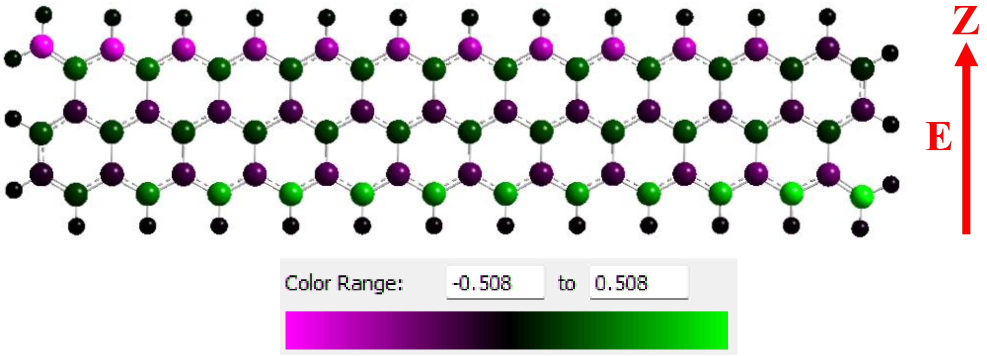

Figure S13: The Mulliken spin population of a zigzag graphene nanoribbon similar to the one used as a molecular bridge in main text Fig. 1 (c), where green and purple colors represent the ↑- and ↓-spins, respectively. The in-plane electric field is applied along the direction indicated by the red arrow.

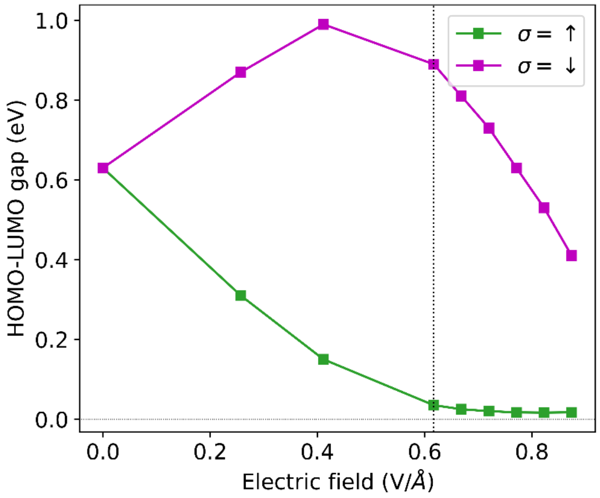

Figure S14: The HOMO-LUMO gap of the ↑-spin (green) and ↓-spin (purple) electrons for different applied electric field strengths.

11. Cartesian coordinates of the H-H-based molecular junction model

Number of Atoms:

**Left Lead:**	**180**
**Left Extended Molecule:**	**09**
**Molecule:**	**2**
**Right Extended Molecule:**	**09**
**Right Lead:**	**180**

1	H	0.000000	0.000000	-187.638000
2	H	0.000000	0.000000	-186.650000
3	H	0.000000	0.000000	-185.662000
4	H	0.000000	0.000000	-184.674000
5	H	0.000000	0.000000	-183.686000
6	H	0.000000	0.000000	-182.698000
7	H	0.000000	0.000000	-181.710000
8	H	0.000000	0.000000	-180.722000
9	H	0.000000	0.000000	-179.734000
10	H	0.000000	0.000000	-178.746000
11	H	0.000000	0.000000	-177.758000
12	H	0.000000	0.000000	-176.770000
13	H	0.000000	0.000000	-175.782000
14	H	0.000000	0.000000	-174.794000
15	H	0.000000	0.000000	-173.806000
16	H	0.000000	0.000000	-172.818000
17	H	0.000000	0.000000	-171.830000
18	H	0.000000	0.000000	-170.842000
19	H	0.000000	0.000000	-169.854000
20	H	0.000000	0.000000	-168.866000
21	H	0.000000	0.000000	-167.878000
22	H	0.000000	0.000000	-166.890000
23	H	0.000000	0.000000	-165.902000
24	H	0.000000	0.000000	-164.914000
25	H	0.000000	0.000000	-163.926000
26	H	0.000000	0.000000	-162.938000
27	H	0.000000	0.000000	-161.950000
28	H	0.000000	0.000000	-160.962000
29	H	0.000000	0.000000	-159.974000
30	H	0.000000	0.000000	-158.986000
31	H	0.000000	0.000000	-157.998000
32	H	0.000000	0.000000	-157.010000
33	H	0.000000	0.000000	-156.022000
34	H	0.000000	0.000000	-155.034000
35	H	0.000000	0.000000	-154.046000
36	H	0.000000	0.000000	-153.058000
37	H	0.000000	0.000000	-152.070000
38	H	0.000000	0.000000	-151.082000
39	H	0.000000	0.000000	-150.094000
40	H	0.000000	0.000000	-149.106000
41	H	0.000000	0.000000	-148.118000
42	H	0.000000	0.000000	-147.130000
43	H	0.000000	0.000000	-146.142000
44	H	0.000000	0.000000	-145.154000
45	H	0.000000	0.000000	-144.166000
46	H	0.000000	0.000000	-143.178000

47	H	0.000000	0.000000	-142.190000
48	H	0.000000	0.000000	-141.202000
49	H	0.000000	0.000000	-140.214000
50	H	0.000000	0.000000	-139.226000
51	H	0.000000	0.000000	-138.238000
52	H	0.000000	0.000000	-137.250000
53	H	0.000000	0.000000	-136.262000
54	H	0.000000	0.000000	-135.274000
55	H	0.000000	0.000000	-134.286000
56	H	0.000000	0.000000	-133.298000
57	H	0.000000	0.000000	-132.310000
58	H	0.000000	0.000000	-131.322000
59	H	0.000000	0.000000	-130.334000
60	H	0.000000	0.000000	-129.346000
61	H	0.000000	0.000000	-128.358000
62	H	0.000000	0.000000	-127.370000
63	H	0.000000	0.000000	-126.382000
64	H	0.000000	0.000000	-125.394000
65	H	0.000000	0.000000	-124.406000
66	H	0.000000	0.000000	-123.418000
67	H	0.000000	0.000000	-122.430000
68	H	0.000000	0.000000	-121.442000
69	H	0.000000	0.000000	-120.454000
70	H	0.000000	0.000000	-119.466000
71	H	0.000000	0.000000	-118.478000
72	H	0.000000	0.000000	-117.490000
73	H	0.000000	0.000000	-116.502000
74	H	0.000000	0.000000	-115.514000
75	H	0.000000	0.000000	-114.526000
76	H	0.000000	0.000000	-113.538000
77	H	0.000000	0.000000	-112.550000
78	H	0.000000	0.000000	-111.562000
79	H	0.000000	0.000000	-110.574000
80	H	0.000000	0.000000	-109.586000
81	H	0.000000	0.000000	-108.598000
82	H	0.000000	0.000000	-107.610000
83	H	0.000000	0.000000	-106.622000
84	H	0.000000	0.000000	-105.634000
85	H	0.000000	0.000000	-104.646000
86	H	0.000000	0.000000	-103.658000
87	H	0.000000	0.000000	-102.670000
88	H	0.000000	0.000000	-101.682000
89	H	0.000000	0.000000	-100.694000
90	H	0.000000	0.000000	-99.706000
91	H	0.000000	0.000000	-98.718000
92	H	0.000000	0.000000	-97.730000
93	H	0.000000	0.000000	-96.742000
94	H	0.000000	0.000000	-95.754000
95	H	0.000000	0.000000	-94.766000
96	H	0.000000	0.000000	-93.778000
97	H	0.000000	0.000000	-92.790000
98	H	0.000000	0.000000	-91.802000
99	H	0.000000	0.000000	-90.814000
100	H	0.000000	0.000000	-89.826000
101	H	0.000000	0.000000	-88.838000
102	H	0.000000	0.000000	-87.850000
103	H	0.000000	0.000000	-86.862000
104	H	0.000000	0.000000	-85.874000

105	H	0.000000	0.000000	-84.886000
106	H	0.000000	0.000000	-83.898000
107	H	0.000000	0.000000	-82.910000
108	H	0.000000	0.000000	-81.922000
109	H	0.000000	0.000000	-80.934000
110	H	0.000000	0.000000	-79.946000
111	H	0.000000	0.000000	-78.958000
112	H	0.000000	0.000000	-77.970000
113	H	0.000000	0.000000	-76.982000
114	H	0.000000	0.000000	-75.994000
115	H	0.000000	0.000000	-75.006000
116	H	0.000000	0.000000	-74.018000
117	H	0.000000	0.000000	-73.030000
118	H	0.000000	0.000000	-72.042000
119	H	0.000000	0.000000	-71.054000
120	H	0.000000	0.000000	-70.066000
121	H	0.000000	0.000000	-69.078000
122	H	0.000000	0.000000	-68.090000
123	H	0.000000	0.000000	-67.102000
124	H	0.000000	0.000000	-66.114000
125	H	0.000000	0.000000	-65.126000
126	H	0.000000	0.000000	-64.138000
127	H	0.000000	0.000000	-63.150000
128	H	0.000000	0.000000	-62.162000
129	H	0.000000	0.000000	-61.174000
130	H	0.000000	0.000000	-60.186000
131	H	0.000000	0.000000	-59.198000
132	H	0.000000	0.000000	-58.210000
133	H	0.000000	0.000000	-57.222000
134	H	0.000000	0.000000	-56.234000
135	H	0.000000	0.000000	-55.246000
136	H	0.000000	0.000000	-54.258000
137	H	0.000000	0.000000	-53.270000
138	H	0.000000	0.000000	-52.282000
139	H	0.000000	0.000000	-51.294000
140	H	0.000000	0.000000	-50.306000
141	H	0.000000	0.000000	-49.318000
142	H	0.000000	0.000000	-48.330000
143	H	0.000000	0.000000	-47.342000
144	H	0.000000	0.000000	-46.354000
145	H	0.000000	0.000000	-45.366000
146	H	0.000000	0.000000	-44.378000
147	H	0.000000	0.000000	-43.390000
148	H	0.000000	0.000000	-42.402000
149	H	0.000000	0.000000	-41.414000
150	H	0.000000	0.000000	-40.426000
151	H	0.000000	0.000000	-39.438000
152	H	0.000000	0.000000	-38.450000
153	H	0.000000	0.000000	-37.462000
154	H	0.000000	0.000000	-36.474000
155	H	0.000000	0.000000	-35.486000
156	H	0.000000	0.000000	-34.498000
157	H	0.000000	0.000000	-33.510000
158	H	0.000000	0.000000	-32.522000
159	H	0.000000	0.000000	-31.534000
160	H	0.000000	0.000000	-30.546000
161	H	0.000000	0.000000	-29.558000
162	H	0.000000	0.000000	-28.570000

163	H	0.000000	0.000000	-27.582000
164	H	0.000000	0.000000	-26.594000
165	H	0.000000	0.000000	-25.606000
166	H	0.000000	0.000000	-24.618000
167	H	0.000000	0.000000	-23.630000
168	H	0.000000	0.000000	-22.642000
169	H	0.000000	0.000000	-21.654000
170	H	0.000000	0.000000	-20.666000
171	H	0.000000	0.000000	-19.678000
172	H	0.000000	0.000000	-18.690000
173	H	0.000000	0.000000	-17.702000
174	H	0.000000	0.000000	-16.714000
175	H	0.000000	0.000000	-15.726000
176	H	0.000000	0.000000	-14.738000
177	H	0.000000	0.000000	-13.750000
178	H	0.000000	0.000000	-12.762000
179	H	0.000000	0.000000	-11.774000
180	H	0.000000	0.000000	-10.786000
181	H	0.000000	0.000000	-9.798000
182	H	0.000000	0.000000	-8.810000
183	H	0.000000	0.000000	-7.822000
184	H	0.000000	0.000000	-6.834000
185	H	0.000000	0.000000	-5.846000
186	H	0.000000	0.000000	-4.858000
187	H	0.000000	0.000000	-3.870000
188	H	0.000000	0.000000	-2.882000
189	H	0.000000	0.000000	-1.894000
190	H	0.000000	0.000000	-0.494000
191	H	0.000000	0.000000	0.494000
192	H	0.000000	0.000000	1.894000
193	H	0.000000	0.000000	2.882000
194	H	0.000000	0.000000	3.870000
195	H	0.000000	0.000000	4.858000
196	H	0.000000	0.000000	5.846000
197	H	0.000000	0.000000	6.834000
198	H	0.000000	0.000000	7.822000
199	H	0.000000	0.000000	8.810000
200	H	0.000000	0.000000	9.798000
201	H	0.000000	0.000000	10.786000
202	H	0.000000	0.000000	11.774000
203	H	0.000000	0.000000	12.762000
204	H	0.000000	0.000000	13.750000
205	H	0.000000	0.000000	14.738000
206	H	0.000000	0.000000	15.726000
207	H	0.000000	0.000000	16.714000
208	H	0.000000	0.000000	17.702000
209	H	0.000000	0.000000	18.690000
210	H	0.000000	0.000000	19.678000
211	H	0.000000	0.000000	20.666000
212	H	0.000000	0.000000	21.654000
213	H	0.000000	0.000000	22.642000
214	H	0.000000	0.000000	23.630000
215	H	0.000000	0.000000	24.618000
216	H	0.000000	0.000000	25.606000
217	H	0.000000	0.000000	26.594000
218	H	0.000000	0.000000	27.582000
219	H	0.000000	0.000000	28.570000
220	H	0.000000	0.000000	29.558000

221	H	0.000000	0.000000	30.546000
222	H	0.000000	0.000000	31.534000
223	H	0.000000	0.000000	32.522000
224	H	0.000000	0.000000	33.510000
225	H	0.000000	0.000000	34.498000
226	H	0.000000	0.000000	35.486000
227	H	0.000000	0.000000	36.474000
228	H	0.000000	0.000000	37.462000
229	H	0.000000	0.000000	38.450000
230	H	0.000000	0.000000	39.438000
231	H	0.000000	0.000000	40.426000
232	H	0.000000	0.000000	41.414000
233	H	0.000000	0.000000	42.402000
234	H	0.000000	0.000000	43.390000
235	H	0.000000	0.000000	44.378000
236	H	0.000000	0.000000	45.366000
237	H	0.000000	0.000000	46.354000
238	H	0.000000	0.000000	47.342000
239	H	0.000000	0.000000	48.330000
240	H	0.000000	0.000000	49.318000
241	H	0.000000	0.000000	50.306000
242	H	0.000000	0.000000	51.294000
243	H	0.000000	0.000000	52.282000
244	H	0.000000	0.000000	53.270000
245	H	0.000000	0.000000	54.258000
246	H	0.000000	0.000000	55.246000
247	H	0.000000	0.000000	56.234000
248	H	0.000000	0.000000	57.222000
249	H	0.000000	0.000000	58.210000
250	H	0.000000	0.000000	59.198000
251	H	0.000000	0.000000	60.186000
252	H	0.000000	0.000000	61.174000
253	H	0.000000	0.000000	62.162000
254	H	0.000000	0.000000	63.150000
255	H	0.000000	0.000000	64.138000
256	H	0.000000	0.000000	65.126000
257	H	0.000000	0.000000	66.114000
258	H	0.000000	0.000000	67.102000
259	H	0.000000	0.000000	68.090000
260	H	0.000000	0.000000	69.078000
261	H	0.000000	0.000000	70.066000
262	H	0.000000	0.000000	71.054000
263	H	0.000000	0.000000	72.042000
264	H	0.000000	0.000000	73.030000
265	H	0.000000	0.000000	74.018000
266	H	0.000000	0.000000	75.006000
267	H	0.000000	0.000000	75.994000
268	H	0.000000	0.000000	76.982000
269	H	0.000000	0.000000	77.970000
270	H	0.000000	0.000000	78.958000
271	H	0.000000	0.000000	79.946000
272	H	0.000000	0.000000	80.934000
273	H	0.000000	0.000000	81.922000
274	H	0.000000	0.000000	82.910000
275	H	0.000000	0.000000	83.898000
276	H	0.000000	0.000000	84.886000
277	H	0.000000	0.000000	85.874000
278	H	0.000000	0.000000	86.862000

279	H	0.000000	0.000000	87.850000
280	H	0.000000	0.000000	88.838000
281	H	0.000000	0.000000	89.826000
282	H	0.000000	0.000000	90.814000
283	H	0.000000	0.000000	91.802000
284	H	0.000000	0.000000	92.790000
285	H	0.000000	0.000000	93.778000
286	H	0.000000	0.000000	94.766000
287	H	0.000000	0.000000	95.754000
288	H	0.000000	0.000000	96.742000
289	H	0.000000	0.000000	97.730000
290	H	0.000000	0.000000	98.718000
291	H	0.000000	0.000000	99.706000
292	H	0.000000	0.000000	100.694000
293	H	0.000000	0.000000	101.682000
294	H	0.000000	0.000000	102.670000
295	H	0.000000	0.000000	103.658000
296	H	0.000000	0.000000	104.646000
297	H	0.000000	0.000000	105.634000
298	H	0.000000	0.000000	106.622000
299	H	0.000000	0.000000	107.610000
300	H	0.000000	0.000000	108.598000
301	H	0.000000	0.000000	109.586000
302	H	0.000000	0.000000	110.574000
303	H	0.000000	0.000000	111.562000
304	H	0.000000	0.000000	112.550000
305	H	0.000000	0.000000	113.538000
306	H	0.000000	0.000000	114.526000
307	H	0.000000	0.000000	115.514000
308	H	0.000000	0.000000	116.502000
309	H	0.000000	0.000000	117.490000
310	H	0.000000	0.000000	118.478000
311	H	0.000000	0.000000	119.466000
312	H	0.000000	0.000000	120.454000
313	H	0.000000	0.000000	121.442000
314	H	0.000000	0.000000	122.430000
315	H	0.000000	0.000000	123.418000
316	H	0.000000	0.000000	124.406000
317	H	0.000000	0.000000	125.394000
318	H	0.000000	0.000000	126.382000
319	H	0.000000	0.000000	127.370000
320	H	0.000000	0.000000	128.358000
321	H	0.000000	0.000000	129.346000
322	H	0.000000	0.000000	130.334000
323	H	0.000000	0.000000	131.322000
324	H	0.000000	0.000000	132.310000
325	H	0.000000	0.000000	133.298000
326	H	0.000000	0.000000	134.286000
327	H	0.000000	0.000000	135.274000
328	H	0.000000	0.000000	136.262000
329	H	0.000000	0.000000	137.250000
330	H	0.000000	0.000000	138.238000
331	H	0.000000	0.000000	139.226000
332	H	0.000000	0.000000	140.214000
333	H	0.000000	0.000000	141.202000
334	H	0.000000	0.000000	142.190000
335	H	0.000000	0.000000	143.178000
336	H	0.000000	0.000000	144.166000

337	H	0.000000	0.000000	145.154000
338	H	0.000000	0.000000	146.142000
339	H	0.000000	0.000000	147.130000
340	H	0.000000	0.000000	148.118000
341	H	0.000000	0.000000	149.106000
342	H	0.000000	0.000000	150.094000
343	H	0.000000	0.000000	151.082000
344	H	0.000000	0.000000	152.070000
345	H	0.000000	0.000000	153.058000
346	H	0.000000	0.000000	154.046000
347	H	0.000000	0.000000	155.034000
348	H	0.000000	0.000000	156.022000
349	H	0.000000	0.000000	157.010000
350	H	0.000000	0.000000	157.998000
351	H	0.000000	0.000000	158.986000
352	H	0.000000	0.000000	159.974000
353	H	0.000000	0.000000	160.962000
354	H	0.000000	0.000000	161.950000
355	H	0.000000	0.000000	162.938000
356	H	0.000000	0.000000	163.926000
357	H	0.000000	0.000000	164.914000
358	H	0.000000	0.000000	165.902000
359	H	0.000000	0.000000	166.890000
360	H	0.000000	0.000000	167.878000
361	H	0.000000	0.000000	168.866000
362	H	0.000000	0.000000	169.854000
363	H	0.000000	0.000000	170.842000
364	H	0.000000	0.000000	171.830000
365	H	0.000000	0.000000	172.818000
366	H	0.000000	0.000000	173.806000
367	H	0.000000	0.000000	174.794000
368	H	0.000000	0.000000	175.782000
369	H	0.000000	0.000000	176.770000
370	H	0.000000	0.000000	177.758000
371	H	0.000000	0.000000	178.746000
372	H	0.000000	0.000000	179.734000
373	H	0.000000	0.000000	180.722000
374	H	0.000000	0.000000	181.710000
375	H	0.000000	0.000000	182.698000
376	H	0.000000	0.000000	183.686000
377	H	0.000000	0.000000	184.674000
378	H	0.000000	0.000000	185.662000
379	H	0.000000	0.000000	186.650000
380	H	0.000000	0.000000	187.638000

12. Cartesian coordinates of the H-He-H-based molecular junction model

Number of Atoms:

**Left Lead:**	**300**
**Left Extended Molecule:**	**50**
**Molecule:**	**3**
**Right Extended Molecule:**	**50**
**Right Lead:**	**300**

1	H	0.000000	0.000000	-347.224000
2	H	0.000000	0.000000	-346.236000
3	H	0.000000	0.000000	-345.248000
4	H	0.000000	0.000000	-344.260000
5	H	0.000000	0.000000	-343.272000
6	H	0.000000	0.000000	-342.284000
7	H	0.000000	0.000000	-341.296000
8	H	0.000000	0.000000	-340.308000
9	H	0.000000	0.000000	-339.320000
10	H	0.000000	0.000000	-338.332000
11	H	0.000000	0.000000	-337.344000
12	H	0.000000	0.000000	-336.356000
13	H	0.000000	0.000000	-335.368000
14	H	0.000000	0.000000	-334.380000
15	H	0.000000	0.000000	-333.392000
16	H	0.000000	0.000000	-332.404000
17	H	0.000000	0.000000	-331.416000
18	H	0.000000	0.000000	-330.428000
19	H	0.000000	0.000000	-329.440000
20	H	0.000000	0.000000	-328.452000
21	H	0.000000	0.000000	-327.464000
22	H	0.000000	0.000000	-326.476000
23	H	0.000000	0.000000	-325.488000
24	H	0.000000	0.000000	-324.500000
25	H	0.000000	0.000000	-323.512000
26	H	0.000000	0.000000	-322.524000
27	H	0.000000	0.000000	-321.536000
28	H	0.000000	0.000000	-320.548000
29	H	0.000000	0.000000	-319.560000
30	H	0.000000	0.000000	-318.572000
31	H	0.000000	0.000000	-317.584000
32	H	0.000000	0.000000	-316.596000
33	H	0.000000	0.000000	-315.608000
34	H	0.000000	0.000000	-314.620000
35	H	0.000000	0.000000	-313.632000
36	H	0.000000	0.000000	-312.644000
37	H	0.000000	0.000000	-311.656000
38	H	0.000000	0.000000	-310.668000
39	H	0.000000	0.000000	-309.680000
40	H	0.000000	0.000000	-308.692000
41	H	0.000000	0.000000	-307.704000
42	H	0.000000	0.000000	-306.716000
43	H	0.000000	0.000000	-305.728000
44	H	0.000000	0.000000	-304.740000
45	H	0.000000	0.000000	-303.752000
46	H	0.000000	0.000000	-302.764000
47	H	0.000000	0.000000	-301.776000

48	H	0.000000	0.000000	-300.788000
49	H	0.000000	0.000000	-299.800000
50	H	0.000000	0.000000	-298.812000
51	H	0.000000	0.000000	-297.824000
52	H	0.000000	0.000000	-296.836000
53	H	0.000000	0.000000	-295.848000
54	H	0.000000	0.000000	-294.860000
55	H	0.000000	0.000000	-293.872000
56	H	0.000000	0.000000	-292.884000
57	H	0.000000	0.000000	-291.896000
58	H	0.000000	0.000000	-290.908000
59	H	0.000000	0.000000	-289.920000
60	H	0.000000	0.000000	-288.932000
61	H	0.000000	0.000000	-287.944000
62	H	0.000000	0.000000	-286.956000
63	H	0.000000	0.000000	-285.968000
64	H	0.000000	0.000000	-284.980000
65	H	0.000000	0.000000	-283.992000
66	H	0.000000	0.000000	-283.004000
67	H	0.000000	0.000000	-282.016000
68	H	0.000000	0.000000	-281.028000
69	H	0.000000	0.000000	-280.040000
70	H	0.000000	0.000000	-279.052000
71	H	0.000000	0.000000	-278.064000
72	H	0.000000	0.000000	-277.076000
73	H	0.000000	0.000000	-276.088000
74	H	0.000000	0.000000	-275.100000
75	H	0.000000	0.000000	-274.112000
76	H	0.000000	0.000000	-273.124000
77	H	0.000000	0.000000	-272.136000
78	H	0.000000	0.000000	-271.148000
79	H	0.000000	0.000000	-270.160000
80	H	0.000000	0.000000	-269.172000
81	H	0.000000	0.000000	-268.184000
82	H	0.000000	0.000000	-267.196000
83	H	0.000000	0.000000	-266.208000
84	H	0.000000	0.000000	-265.220000
85	H	0.000000	0.000000	-264.232000
86	H	0.000000	0.000000	-263.244000
87	H	0.000000	0.000000	-262.256000
88	H	0.000000	0.000000	-261.268000
89	H	0.000000	0.000000	-260.280000
90	H	0.000000	0.000000	-259.292000
91	H	0.000000	0.000000	-258.304000
92	H	0.000000	0.000000	-257.316000
93	H	0.000000	0.000000	-256.328000
94	H	0.000000	0.000000	-255.340000
95	H	0.000000	0.000000	-254.352000
96	H	0.000000	0.000000	-253.364000
97	H	0.000000	0.000000	-252.376000
98	H	0.000000	0.000000	-251.388000
99	H	0.000000	0.000000	-250.400000
100	H	0.000000	0.000000	-249.412000
101	H	0.000000	0.000000	-248.424000
102	H	0.000000	0.000000	-247.436000
103	H	0.000000	0.000000	-246.448000
104	H	0.000000	0.000000	-245.460000
105	H	0.000000	0.000000	-244.472000

106	H	0.000000	0.000000	-243.484000
107	H	0.000000	0.000000	-242.496000
108	H	0.000000	0.000000	-241.508000
109	H	0.000000	0.000000	-240.520000
110	H	0.000000	0.000000	-239.532000
111	H	0.000000	0.000000	-238.544000
112	H	0.000000	0.000000	-237.556000
113	H	0.000000	0.000000	-236.568000
114	H	0.000000	0.000000	-235.580000
115	H	0.000000	0.000000	-234.592000
116	H	0.000000	0.000000	-233.604000
117	H	0.000000	0.000000	-232.616000
118	H	0.000000	0.000000	-231.628000
119	H	0.000000	0.000000	-230.640000
120	H	0.000000	0.000000	-229.652000
121	H	0.000000	0.000000	-228.664000
122	H	0.000000	0.000000	-227.676000
123	H	0.000000	0.000000	-226.688000
124	H	0.000000	0.000000	-225.700000
125	H	0.000000	0.000000	-224.712000
126	H	0.000000	0.000000	-223.724000
127	H	0.000000	0.000000	-222.736000
128	H	0.000000	0.000000	-221.748000
129	H	0.000000	0.000000	-220.760000
130	H	0.000000	0.000000	-219.772000
131	H	0.000000	0.000000	-218.784000
132	H	0.000000	0.000000	-217.796000
133	H	0.000000	0.000000	-216.808000
134	H	0.000000	0.000000	-215.820000
135	H	0.000000	0.000000	-214.832000
136	H	0.000000	0.000000	-213.844000
137	H	0.000000	0.000000	-212.856000
138	H	0.000000	0.000000	-211.868000
139	H	0.000000	0.000000	-210.880000
140	H	0.000000	0.000000	-209.892000
141	H	0.000000	0.000000	-208.904000
142	H	0.000000	0.000000	-207.916000
143	H	0.000000	0.000000	-206.928000
144	H	0.000000	0.000000	-205.940000
145	H	0.000000	0.000000	-204.952000
146	H	0.000000	0.000000	-203.964000
147	H	0.000000	0.000000	-202.976000
148	H	0.000000	0.000000	-201.988000
149	H	0.000000	0.000000	-201.000000
150	H	0.000000	0.000000	-200.012000
151	H	0.000000	0.000000	-199.024000
152	H	0.000000	0.000000	-198.036000
153	H	0.000000	0.000000	-197.048000
154	H	0.000000	0.000000	-196.060000
155	H	0.000000	0.000000	-195.072000
156	H	0.000000	0.000000	-194.084000
157	H	0.000000	0.000000	-193.096000
158	H	0.000000	0.000000	-192.108000
159	H	0.000000	0.000000	-191.120000
160	H	0.000000	0.000000	-190.132000
161	H	0.000000	0.000000	-189.144000
162	H	0.000000	0.000000	-188.156000
163	H	0.000000	0.000000	-187.168000

164	H	0.000000	0.000000	-186.180000
165	H	0.000000	0.000000	-185.192000
166	H	0.000000	0.000000	-184.204000
167	H	0.000000	0.000000	-183.216000
168	H	0.000000	0.000000	-182.228000
169	H	0.000000	0.000000	-181.240000
170	H	0.000000	0.000000	-180.252000
171	H	0.000000	0.000000	-179.264000
172	H	0.000000	0.000000	-178.276000
173	H	0.000000	0.000000	-177.288000
174	H	0.000000	0.000000	-176.300000
175	H	0.000000	0.000000	-175.312000
176	H	0.000000	0.000000	-174.324000
177	H	0.000000	0.000000	-173.336000
178	H	0.000000	0.000000	-172.348000
179	H	0.000000	0.000000	-171.360000
180	H	0.000000	0.000000	-170.372000
181	H	0.000000	0.000000	-169.384000
182	H	0.000000	0.000000	-168.396000
183	H	0.000000	0.000000	-167.408000
184	H	0.000000	0.000000	-166.420000
185	H	0.000000	0.000000	-165.432000
186	H	0.000000	0.000000	-164.444000
187	H	0.000000	0.000000	-163.456000
188	H	0.000000	0.000000	-162.468000
189	H	0.000000	0.000000	-161.480000
190	H	0.000000	0.000000	-160.492000
191	H	0.000000	0.000000	-159.504000
192	H	0.000000	0.000000	-158.516000
193	H	0.000000	0.000000	-157.528000
194	H	0.000000	0.000000	-156.540000
195	H	0.000000	0.000000	-155.552000
196	H	0.000000	0.000000	-154.564000
197	H	0.000000	0.000000	-153.576000
198	H	0.000000	0.000000	-152.588000
199	H	0.000000	0.000000	-151.600000
200	H	0.000000	0.000000	-150.612000
201	H	0.000000	0.000000	-149.624000
202	H	0.000000	0.000000	-148.636000
203	H	0.000000	0.000000	-147.648000
204	H	0.000000	0.000000	-146.660000
205	H	0.000000	0.000000	-145.672000
206	H	0.000000	0.000000	-144.684000
207	H	0.000000	0.000000	-143.696000
208	H	0.000000	0.000000	-142.708000
209	H	0.000000	0.000000	-141.720000
210	H	0.000000	0.000000	-140.732000
211	H	0.000000	0.000000	-139.744000
212	H	0.000000	0.000000	-138.756000
213	H	0.000000	0.000000	-137.768000
214	H	0.000000	0.000000	-136.780000
215	H	0.000000	0.000000	-135.792000
216	H	0.000000	0.000000	-134.804000
217	H	0.000000	0.000000	-133.816000
218	H	0.000000	0.000000	-132.828000
219	H	0.000000	0.000000	-131.840000
220	H	0.000000	0.000000	-130.852000
221	H	0.000000	0.000000	-129.864000

222	H	0.000000	0.000000	-128.876000
223	H	0.000000	0.000000	-127.888000
224	H	0.000000	0.000000	-126.900000
225	H	0.000000	0.000000	-125.912000
226	H	0.000000	0.000000	-124.924000
227	H	0.000000	0.000000	-123.936000
228	H	0.000000	0.000000	-122.948000
229	H	0.000000	0.000000	-121.960000
230	H	0.000000	0.000000	-120.972000
231	H	0.000000	0.000000	-119.984000
232	H	0.000000	0.000000	-118.996000
233	H	0.000000	0.000000	-118.008000
234	H	0.000000	0.000000	-117.020000
235	H	0.000000	0.000000	-116.032000
236	H	0.000000	0.000000	-115.044000
237	H	0.000000	0.000000	-114.056000
238	H	0.000000	0.000000	-113.068000
239	H	0.000000	0.000000	-112.080000
240	H	0.000000	0.000000	-111.092000
241	H	0.000000	0.000000	-110.104000
242	H	0.000000	0.000000	-109.116000
243	H	0.000000	0.000000	-108.128000
244	H	0.000000	0.000000	-107.140000
245	H	0.000000	0.000000	-106.152000
246	H	0.000000	0.000000	-105.164000
247	H	0.000000	0.000000	-104.176000
248	H	0.000000	0.000000	-103.188000
249	H	0.000000	0.000000	-102.200000
250	H	0.000000	0.000000	-101.212000
251	H	0.000000	0.000000	-100.224000
252	H	0.000000	0.000000	-99.236000
253	H	0.000000	0.000000	-98.248000
254	H	0.000000	0.000000	-97.260000
255	H	0.000000	0.000000	-96.272000
256	H	0.000000	0.000000	-95.284000
257	H	0.000000	0.000000	-94.296000
258	H	0.000000	0.000000	-93.308000
259	H	0.000000	0.000000	-92.320000
260	H	0.000000	0.000000	-91.332000
261	H	0.000000	0.000000	-90.344000
262	H	0.000000	0.000000	-89.356000
263	H	0.000000	0.000000	-88.368000
264	H	0.000000	0.000000	-87.380000
265	H	0.000000	0.000000	-86.392000
266	H	0.000000	0.000000	-85.404000
267	H	0.000000	0.000000	-84.416000
268	H	0.000000	0.000000	-83.428000
269	H	0.000000	0.000000	-82.440000
270	H	0.000000	0.000000	-81.452000
271	H	0.000000	0.000000	-80.464000
272	H	0.000000	0.000000	-79.476000
273	H	0.000000	0.000000	-78.488000
274	H	0.000000	0.000000	-77.500000
275	H	0.000000	0.000000	-76.512000
276	H	0.000000	0.000000	-75.524000
277	H	0.000000	0.000000	-74.536000
278	H	0.000000	0.000000	-73.548000
279	H	0.000000	0.000000	-72.560000

280	H	0.000000	0.000000	-71.572000
281	H	0.000000	0.000000	-70.584000
282	H	0.000000	0.000000	-69.596000
283	H	0.000000	0.000000	-68.608000
284	H	0.000000	0.000000	-67.620000
285	H	0.000000	0.000000	-66.632000
286	H	0.000000	0.000000	-65.644000
287	H	0.000000	0.000000	-64.656000
288	H	0.000000	0.000000	-63.668000
289	H	0.000000	0.000000	-62.680000
290	H	0.000000	0.000000	-61.692000
291	H	0.000000	0.000000	-60.704000
292	H	0.000000	0.000000	-59.716000
293	H	0.000000	0.000000	-58.728000
294	H	0.000000	0.000000	-57.740000
295	H	0.000000	0.000000	-56.752000
296	H	0.000000	0.000000	-55.764000
297	H	0.000000	0.000000	-54.776000
298	H	0.000000	0.000000	-53.788000
299	H	0.000000	0.000000	-52.800000
300	H	0.000000	0.000000	-51.812000
301	H	0.000000	0.000000	-50.824000
302	H	0.000000	0.000000	-49.836000
303	H	0.000000	0.000000	-48.848000
304	H	0.000000	0.000000	-47.860000
305	H	0.000000	0.000000	-46.872000
306	H	0.000000	0.000000	-45.884000
307	H	0.000000	0.000000	-44.896000
308	H	0.000000	0.000000	-43.908000
309	H	0.000000	0.000000	-42.920000
310	H	0.000000	0.000000	-41.932000
311	H	0.000000	0.000000	-40.944000
312	H	0.000000	0.000000	-39.956000
313	H	0.000000	0.000000	-38.968000
314	H	0.000000	0.000000	-37.980000
315	H	0.000000	0.000000	-36.992000
316	H	0.000000	0.000000	-36.004000
317	H	0.000000	0.000000	-35.016000
318	H	0.000000	0.000000	-34.028000
319	H	0.000000	0.000000	-33.040000
320	H	0.000000	0.000000	-32.052000
321	H	0.000000	0.000000	-31.064000
322	H	0.000000	0.000000	-30.076000
323	H	0.000000	0.000000	-29.088000
324	H	0.000000	0.000000	-28.100000
325	H	0.000000	0.000000	-27.112000
326	H	0.000000	0.000000	-26.124000
327	H	0.000000	0.000000	-25.136000
328	H	0.000000	0.000000	-24.148000
329	H	0.000000	0.000000	-23.160000
330	H	0.000000	0.000000	-22.172000
331	H	0.000000	0.000000	-21.184000
332	H	0.000000	0.000000	-20.196000
333	H	0.000000	0.000000	-19.208000
334	H	0.000000	0.000000	-18.220000
335	H	0.000000	0.000000	-17.232000
336	H	0.000000	0.000000	-16.244000
337	H	0.000000	0.000000	-15.256000

338	H	0.000000	0.000000	-14.268000
339	H	0.000000	0.000000	-13.280000
340	H	0.000000	0.000000	-12.292000
341	H	0.000000	0.000000	-11.304000
342	H	0.000000	0.000000	-10.316000
343	H	0.000000	0.000000	-9.328000
344	H	0.000000	0.000000	-8.340000
345	H	0.000000	0.000000	-7.352000
346	H	0.000000	0.000000	-6.364000
347	H	0.000000	0.000000	-5.376000
348	H	0.000000	0.000000	-4.388000
349	H	0.000000	0.000000	-3.400000
350	H	0.000000	0.000000	-2.000000
351	H	-1.625000	0.000000	0.000000
352	He	0.000000	0.000000	0.000000
353	H	1.625000	0.000000	0.000000
354	H	0.000000	0.000000	2.000000
355	H	0.000000	0.000000	3.400000
356	H	0.000000	0.000000	4.388000
357	H	0.000000	0.000000	5.376000
358	H	0.000000	0.000000	6.364000
359	H	0.000000	0.000000	7.352000
360	H	0.000000	0.000000	8.340000
361	H	0.000000	0.000000	9.328000
362	H	0.000000	0.000000	10.316000
363	H	0.000000	0.000000	11.304000
364	H	0.000000	0.000000	12.292000
365	H	0.000000	0.000000	13.280000
366	H	0.000000	0.000000	14.268000
367	H	0.000000	0.000000	15.256000
368	H	0.000000	0.000000	16.244000
369	H	0.000000	0.000000	17.232000
370	H	0.000000	0.000000	18.220000
371	H	0.000000	0.000000	19.208000
372	H	0.000000	0.000000	20.196000
373	H	0.000000	0.000000	21.184000
374	H	0.000000	0.000000	22.172000
375	H	0.000000	0.000000	23.160000
376	H	0.000000	0.000000	24.148000
377	H	0.000000	0.000000	25.136000
378	H	0.000000	0.000000	26.124000
379	H	0.000000	0.000000	27.112000
380	H	0.000000	0.000000	28.100000
381	H	0.000000	0.000000	29.088000
382	H	0.000000	0.000000	30.076000
383	H	0.000000	0.000000	31.064000
384	H	0.000000	0.000000	32.052000
385	H	0.000000	0.000000	33.040000
386	H	0.000000	0.000000	34.028000
387	H	0.000000	0.000000	35.016000
388	H	0.000000	0.000000	36.004000
389	H	0.000000	0.000000	36.992000
390	H	0.000000	0.000000	37.980000
391	H	0.000000	0.000000	38.968000
392	H	0.000000	0.000000	39.956000
393	H	0.000000	0.000000	40.944000
394	H	0.000000	0.000000	41.932000
395	H	0.000000	0.000000	42.920000

396	H	0.000000	0.000000	43.908000
397	H	0.000000	0.000000	44.896000
398	H	0.000000	0.000000	45.884000
399	H	0.000000	0.000000	46.872000
400	H	0.000000	0.000000	47.860000
401	H	0.000000	0.000000	48.848000
402	H	0.000000	0.000000	49.836000
403	H	0.000000	0.000000	50.824000
404	H	0.000000	0.000000	51.812000
405	H	0.000000	0.000000	52.800000
406	H	0.000000	0.000000	53.788000
407	H	0.000000	0.000000	54.776000
408	H	0.000000	0.000000	55.764000
409	H	0.000000	0.000000	56.752000
410	H	0.000000	0.000000	57.740000
411	H	0.000000	0.000000	58.728000
412	H	0.000000	0.000000	59.716000
413	H	0.000000	0.000000	60.704000
414	H	0.000000	0.000000	61.692000
415	H	0.000000	0.000000	62.680000
416	H	0.000000	0.000000	63.668000
417	H	0.000000	0.000000	64.656000
418	H	0.000000	0.000000	65.644000
419	H	0.000000	0.000000	66.632000
420	H	0.000000	0.000000	67.620000
421	H	0.000000	0.000000	68.608000
422	H	0.000000	0.000000	69.596000
423	H	0.000000	0.000000	70.584000
424	H	0.000000	0.000000	71.572000
425	H	0.000000	0.000000	72.560000
426	H	0.000000	0.000000	73.548000
427	H	0.000000	0.000000	74.536000
428	H	0.000000	0.000000	75.524000
429	H	0.000000	0.000000	76.512000
430	H	0.000000	0.000000	77.500000
431	H	0.000000	0.000000	78.488000
432	H	0.000000	0.000000	79.476000
433	H	0.000000	0.000000	80.464000
434	H	0.000000	0.000000	81.452000
435	H	0.000000	0.000000	82.440000
436	H	0.000000	0.000000	83.428000
437	H	0.000000	0.000000	84.416000
438	H	0.000000	0.000000	85.404000
439	H	0.000000	0.000000	86.392000
440	H	0.000000	0.000000	87.380000
441	H	0.000000	0.000000	88.368000
442	H	0.000000	0.000000	89.356000
443	H	0.000000	0.000000	90.344000
444	H	0.000000	0.000000	91.332000
445	H	0.000000	0.000000	92.320000
446	H	0.000000	0.000000	93.308000
447	H	0.000000	0.000000	94.296000
448	H	0.000000	0.000000	95.284000
449	H	0.000000	0.000000	96.272000
450	H	0.000000	0.000000	97.260000
451	H	0.000000	0.000000	98.248000
452	H	0.000000	0.000000	99.236000
453	H	0.000000	0.000000	100.224000

454	H	0.000000	0.000000	101.212000
455	H	0.000000	0.000000	102.200000
456	H	0.000000	0.000000	103.188000
457	H	0.000000	0.000000	104.176000
458	H	0.000000	0.000000	105.164000
459	H	0.000000	0.000000	106.152000
460	H	0.000000	0.000000	107.140000
461	H	0.000000	0.000000	108.128000
462	H	0.000000	0.000000	109.116000
463	H	0.000000	0.000000	110.104000
464	H	0.000000	0.000000	111.092000
465	H	0.000000	0.000000	112.080000
466	H	0.000000	0.000000	113.068000
467	H	0.000000	0.000000	114.056000
468	H	0.000000	0.000000	115.044000
469	H	0.000000	0.000000	116.032000
470	H	0.000000	0.000000	117.020000
471	H	0.000000	0.000000	118.008000
472	H	0.000000	0.000000	118.996000
473	H	0.000000	0.000000	119.984000
474	H	0.000000	0.000000	120.972000
475	H	0.000000	0.000000	121.960000
476	H	0.000000	0.000000	122.948000
477	H	0.000000	0.000000	123.936000
478	H	0.000000	0.000000	124.924000
479	H	0.000000	0.000000	125.912000
480	H	0.000000	0.000000	126.900000
481	H	0.000000	0.000000	127.888000
482	H	0.000000	0.000000	128.876000
483	H	0.000000	0.000000	129.864000
484	H	0.000000	0.000000	130.852000
485	H	0.000000	0.000000	131.840000
486	H	0.000000	0.000000	132.828000
487	H	0.000000	0.000000	133.816000
488	H	0.000000	0.000000	134.804000
489	H	0.000000	0.000000	135.792000
490	H	0.000000	0.000000	136.780000
491	H	0.000000	0.000000	137.768000
492	H	0.000000	0.000000	138.756000
493	H	0.000000	0.000000	139.744000
494	H	0.000000	0.000000	140.732000
495	H	0.000000	0.000000	141.720000
496	H	0.000000	0.000000	142.708000
497	H	0.000000	0.000000	143.696000
498	H	0.000000	0.000000	144.684000
499	H	0.000000	0.000000	145.672000
500	H	0.000000	0.000000	146.660000
501	H	0.000000	0.000000	147.648000
502	H	0.000000	0.000000	148.636000
503	H	0.000000	0.000000	149.624000
504	H	0.000000	0.000000	150.612000
505	H	0.000000	0.000000	151.600000
506	H	0.000000	0.000000	152.588000
507	H	0.000000	0.000000	153.576000
508	H	0.000000	0.000000	154.564000
509	H	0.000000	0.000000	155.552000
510	H	0.000000	0.000000	156.540000
511	H	0.000000	0.000000	157.528000

512	H	0.000000	0.000000	158.516000
513	H	0.000000	0.000000	159.504000
514	H	0.000000	0.000000	160.492000
515	H	0.000000	0.000000	161.480000
516	H	0.000000	0.000000	162.468000
517	H	0.000000	0.000000	163.456000
518	H	0.000000	0.000000	164.444000
519	H	0.000000	0.000000	165.432000
520	H	0.000000	0.000000	166.420000
521	H	0.000000	0.000000	167.408000
522	H	0.000000	0.000000	168.396000
523	H	0.000000	0.000000	169.384000
524	H	0.000000	0.000000	170.372000
525	H	0.000000	0.000000	171.360000
526	H	0.000000	0.000000	172.348000
527	H	0.000000	0.000000	173.336000
528	H	0.000000	0.000000	174.324000
529	H	0.000000	0.000000	175.312000
530	H	0.000000	0.000000	176.300000
531	H	0.000000	0.000000	177.288000
532	H	0.000000	0.000000	178.276000
533	H	0.000000	0.000000	179.264000
534	H	0.000000	0.000000	180.252000
535	H	0.000000	0.000000	181.240000
536	H	0.000000	0.000000	182.228000
537	H	0.000000	0.000000	183.216000
538	H	0.000000	0.000000	184.204000
539	H	0.000000	0.000000	185.192000
540	H	0.000000	0.000000	186.180000
541	H	0.000000	0.000000	187.168000
542	H	0.000000	0.000000	188.156000
543	H	0.000000	0.000000	189.144000
544	H	0.000000	0.000000	190.132000
545	H	0.000000	0.000000	191.120000
546	H	0.000000	0.000000	192.108000
547	H	0.000000	0.000000	193.096000
548	H	0.000000	0.000000	194.084000
549	H	0.000000	0.000000	195.072000
550	H	0.000000	0.000000	196.060000
551	H	0.000000	0.000000	197.048000
552	H	0.000000	0.000000	198.036000
553	H	0.000000	0.000000	199.024000
554	H	0.000000	0.000000	200.012000
555	H	0.000000	0.000000	201.000000
556	H	0.000000	0.000000	201.988000
557	H	0.000000	0.000000	202.976000
558	H	0.000000	0.000000	203.964000
559	H	0.000000	0.000000	204.952000
560	H	0.000000	0.000000	205.940000
561	H	0.000000	0.000000	206.928000
562	H	0.000000	0.000000	207.916000
563	H	0.000000	0.000000	208.904000
564	H	0.000000	0.000000	209.892000
565	H	0.000000	0.000000	210.880000
566	H	0.000000	0.000000	211.868000
567	H	0.000000	0.000000	212.856000
568	H	0.000000	0.000000	213.844000
569	H	0.000000	0.000000	214.832000

570	H	0.000000	0.000000	215.820000
571	H	0.000000	0.000000	216.808000
572	H	0.000000	0.000000	217.796000
573	H	0.000000	0.000000	218.784000
574	H	0.000000	0.000000	219.772000
575	H	0.000000	0.000000	220.760000
576	H	0.000000	0.000000	221.748000
577	H	0.000000	0.000000	222.736000
578	H	0.000000	0.000000	223.724000
579	H	0.000000	0.000000	224.712000
580	H	0.000000	0.000000	225.700000
581	H	0.000000	0.000000	226.688000
582	H	0.000000	0.000000	227.676000
583	H	0.000000	0.000000	228.664000
584	H	0.000000	0.000000	229.652000
585	H	0.000000	0.000000	230.640000
586	H	0.000000	0.000000	231.628000
587	H	0.000000	0.000000	232.616000
588	H	0.000000	0.000000	233.604000
589	H	0.000000	0.000000	234.592000
590	H	0.000000	0.000000	235.580000
591	H	0.000000	0.000000	236.568000
592	H	0.000000	0.000000	237.556000
593	H	0.000000	0.000000	238.544000
594	H	0.000000	0.000000	239.532000
595	H	0.000000	0.000000	240.520000
596	H	0.000000	0.000000	241.508000
597	H	0.000000	0.000000	242.496000
598	H	0.000000	0.000000	243.484000
599	H	0.000000	0.000000	244.472000
600	H	0.000000	0.000000	245.460000
601	H	0.000000	0.000000	246.448000
602	H	0.000000	0.000000	247.436000
603	H	0.000000	0.000000	248.424000
604	H	0.000000	0.000000	249.412000
605	H	0.000000	0.000000	250.400000
606	H	0.000000	0.000000	251.388000
607	H	0.000000	0.000000	252.376000
608	H	0.000000	0.000000	253.364000
609	H	0.000000	0.000000	254.352000
610	H	0.000000	0.000000	255.340000
611	H	0.000000	0.000000	256.328000
612	H	0.000000	0.000000	257.316000
613	H	0.000000	0.000000	258.304000
614	H	0.000000	0.000000	259.292000
615	H	0.000000	0.000000	260.280000
616	H	0.000000	0.000000	261.268000
617	H	0.000000	0.000000	262.256000
618	H	0.000000	0.000000	263.244000
619	H	0.000000	0.000000	264.232000
620	H	0.000000	0.000000	265.220000
621	H	0.000000	0.000000	266.208000
622	H	0.000000	0.000000	267.196000
623	H	0.000000	0.000000	268.184000
624	H	0.000000	0.000000	269.172000
625	H	0.000000	0.000000	270.160000
626	H	0.000000	0.000000	271.148000
627	H	0.000000	0.000000	272.136000

628	H	0.000000	0.000000	273.124000
629	H	0.000000	0.000000	274.112000
630	H	0.000000	0.000000	275.100000
631	H	0.000000	0.000000	276.088000
632	H	0.000000	0.000000	277.076000
633	H	0.000000	0.000000	278.064000
634	H	0.000000	0.000000	279.052000
635	H	0.000000	0.000000	280.040000
636	H	0.000000	0.000000	281.028000
637	H	0.000000	0.000000	282.016000
638	H	0.000000	0.000000	283.004000
639	H	0.000000	0.000000	283.992000
640	H	0.000000	0.000000	284.980000
641	H	0.000000	0.000000	285.968000
642	H	0.000000	0.000000	286.956000
643	H	0.000000	0.000000	287.944000
644	H	0.000000	0.000000	288.932000
645	H	0.000000	0.000000	289.920000
646	H	0.000000	0.000000	290.908000
647	H	0.000000	0.000000	291.896000
648	H	0.000000	0.000000	292.884000
649	H	0.000000	0.000000	293.872000
650	H	0.000000	0.000000	294.860000
651	H	0.000000	0.000000	295.848000
652	H	0.000000	0.000000	296.836000
653	H	0.000000	0.000000	297.824000
654	H	0.000000	0.000000	298.812000
655	H	0.000000	0.000000	299.800000
656	H	0.000000	0.000000	300.788000
657	H	0.000000	0.000000	301.776000
658	H	0.000000	0.000000	302.764000
659	H	0.000000	0.000000	303.752000
660	H	0.000000	0.000000	304.740000
661	H	0.000000	0.000000	305.728000
662	H	0.000000	0.000000	306.716000
663	H	0.000000	0.000000	307.704000
664	H	0.000000	0.000000	308.692000
665	H	0.000000	0.000000	309.680000
666	H	0.000000	0.000000	310.668000
667	H	0.000000	0.000000	311.656000
668	H	0.000000	0.000000	312.644000
669	H	0.000000	0.000000	313.632000
670	H	0.000000	0.000000	314.620000
671	H	0.000000	0.000000	315.608000
672	H	0.000000	0.000000	316.596000
673	H	0.000000	0.000000	317.584000
674	H	0.000000	0.000000	318.572000
675	H	0.000000	0.000000	319.560000
676	H	0.000000	0.000000	320.548000
677	H	0.000000	0.000000	321.536000
678	H	0.000000	0.000000	322.524000
679	H	0.000000	0.000000	323.512000
680	H	0.000000	0.000000	324.500000
681	H	0.000000	0.000000	325.488000
682	H	0.000000	0.000000	326.476000
683	H	0.000000	0.000000	327.464000
684	H	0.000000	0.000000	328.452000
685	H	0.000000	0.000000	329.440000

686	H	0.000000	0.000000	330.428000
687	H	0.000000	0.000000	331.416000
688	H	0.000000	0.000000	332.404000
689	H	0.000000	0.000000	333.392000
690	H	0.000000	0.000000	334.380000
691	H	0.000000	0.000000	335.368000
692	H	0.000000	0.000000	336.356000
693	H	0.000000	0.000000	337.344000
694	H	0.000000	0.000000	338.332000
695	H	0.000000	0.000000	339.320000
696	H	0.000000	0.000000	340.308000
697	H	0.000000	0.000000	341.296000
698	H	0.000000	0.000000	342.284000
699	H	0.000000	0.000000	343.272000
700	H	0.000000	0.000000	344.260000
701	H	0.000000	0.000000	345.248000
702	H	0.000000	0.000000	346.236000
703	H	0.000000	0.000000	347.224000

13. Cartesian coordinates of the zigzag GNR-based molecular junction model

Number of Atoms:

**Left Lead:**	**140**
**Left Extended Molecule:**	**65**
**Molecule:**	**24**
**Right Extended Molecule:**	**65**
**Right Lead:**	**140**

1	H	40.818694	0.000000	-13.196077
2	H	38.372687	0.000000	-17.516738
3	H	39.588001	0.000000	-15.352053
4	C	39.872013	0.000000	-12.647006
5	C	37.414740	0.000000	-16.987565
6	C	38.634799	0.000000	-14.812433
7	H	40.802671	0.000000	-10.711085
8	H	36.233721	0.000000	-18.781745
9	C	39.863272	0.000000	-11.272459
10	C	36.231658	0.000000	-17.687392
11	C	38.645023	0.000000	-13.403231
12	C	37.431767	0.000000	-15.546348
13	C	38.626599	0.000000	-10.531005
14	C	34.959516	0.000000	-17.008574
15	C	37.392844	0.000000	-12.688685
16	C	36.174721	0.000000	-14.840388
17	H	39.540321	0.000000	-8.569435
18	H	33.747802	0.000000	-18.801436
19	C	38.596967	0.000000	-9.125873
20	C	33.739474	0.000000	-17.706231
21	C	37.381210	0.000000	-11.258841
22	C	34.942733	0.000000	-15.566193
23	C	36.160089	0.000000	-13.411454
24	C	37.379906	0.000000	-8.409345
25	C	32.498792	0.000000	-17.031402
26	C	36.137709	0.000000	-10.551576
27	C	33.696408	0.000000	-14.863932
28	C	34.909355	0.000000	-12.703399
29	H	38.293839	0.000000	-6.431513
30	H	31.273237	0.000000	-18.832819
31	C	37.345762	0.000000	-6.977945
32	C	31.253903	0.000000	-17.738714
33	C	36.128729	0.000000	-9.123332
34	C	32.467155	0.000000	-15.591196
35	C	34.896544	0.000000	-11.274503
36	C	33.677588	0.000000	-13.427705
37	C	36.149865	0.000000	-6.293762
38	C	30.051858	0.000000	-17.065380
39	C	34.884648	0.000000	-8.406990
40	C	31.212744	0.000000	-14.893113
41	C	33.652303	0.000000	-10.566663
42	C	32.430370	0.000000	-12.725102
43	H	36.175326	0.000000	-5.203119
44	H	29.129793	0.000000	-17.648424
45	C	34.887192	0.000000	-6.975765
46	C	29.986891	0.000000	-15.631792

47	C	33.641227	0.000000	-9.131825
48	C	31.194399	0.000000	-13.453960
49	C	32.416322	0.000000	-11.292054
50	C	33.630516	0.000000	-6.266276
51	C	28.731849	0.000000	-14.919386
52	C	32.397705	0.000000	-8.427413
53	C	29.950510	0.000000	-12.750224
54	C	31.168014	0.000000	-10.585379
55	H	34.498208	0.000000	-4.259993
56	H	27.458127	0.000000	-16.695817
57	C	32.390841	0.000000	-6.991053
58	C	28.715389	0.000000	-13.483491
59	C	31.156634	0.000000	-9.151904
60	C	29.933075	0.000000	-11.313277
61	C	33.575359	0.000000	-4.841411
62	C	27.481775	0.000000	-15.605335
63	C	32.375007	0.000000	-4.164220
64	C	26.283421	0.000000	-14.924582
65	C	31.137558	0.000000	-6.283313
66	C	27.463626	0.000000	-12.773112
67	C	29.908861	0.000000	-8.445995
68	C	28.685680	0.000000	-10.606659
69	H	32.397799	0.000000	-3.073929
70	H	25.360282	0.000000	-15.505153
71	C	31.125967	0.000000	-4.852409
72	C	26.230792	0.000000	-13.499516
73	C	28.672005	0.000000	-9.172364
74	C	29.894877	0.000000	-7.010671
75	C	27.447817	0.000000	-11.333282
76	C	28.649166	0.000000	-6.306482
77	C	26.202994	0.000000	-10.627573
78	C	29.869337	0.000000	-4.142272
79	C	24.975203	0.000000	-12.787484
80	C	27.422206	0.000000	-8.464863
81	H	30.740295	0.000000	-2.137295
82	H	23.704304	0.000000	-14.566040
83	C	28.635166	0.000000	-4.868489
84	C	24.962801	0.000000	-11.355566
85	C	26.185700	0.000000	-9.191374
86	C	27.408805	0.000000	-7.030730
87	C	29.817463	0.000000	-2.718236
88	C	23.727560	0.000000	-13.475817
89	C	28.618382	0.000000	-2.039287
90	C	22.528345	0.000000	-12.797047
91	C	26.160048	0.000000	-6.323836
92	C	24.936901	0.000000	-8.484436
93	C	23.710727	0.000000	-10.646690
94	C	27.382985	0.000000	-4.159709
95	H	28.641789	0.000000	-0.949083
96	H	21.605615	0.000000	-13.378122
97	C	22.476443	0.000000	-11.372931
98	C	27.370515	0.000000	-2.727607
99	C	24.923408	0.000000	-7.050308
100	C	26.142786	0.000000	-4.887634
101	C	23.696611	0.000000	-9.208667
102	C	24.897891	0.000000	-4.181990
103	C	22.450942	0.000000	-8.504434
104	C	23.673739	0.000000	-6.342938

105	C	26.115316	0.000000	-2.015935
106	C	21.220250	0.000000	-10.662916
107	H	19.948865	0.000000	-12.441837
108	H	26.986053	0.000000	-0.010200
109	C	23.659898	0.000000	-4.908429
110	C	22.436674	0.000000	-7.069200
111	C	24.882075	0.000000	-2.742230
112	C	21.208218	0.000000	-9.231686
113	C	19.971216	0.000000	-11.351545
114	C	26.062744	0.000000	-0.590485
115	C	18.771021	0.000000	-10.675076
116	C	24.865064	0.000000	0.090412
117	C	22.412738	0.000000	-4.201996
118	C	21.188928	0.000000	-6.363407
119	C	23.630663	0.000000	-2.032264
120	C	19.955516	0.000000	-8.524137
121	H	17.848524	0.000000	-11.256914
122	H	24.889107	0.000000	1.180824
123	C	21.177096	0.000000	-4.929688
124	C	22.395378	0.000000	-2.765129
125	C	19.947787	0.000000	-7.087526
126	C	18.715255	0.000000	-9.249443
127	C	23.613952	0.000000	-0.595487
128	C	19.929552	0.000000	-4.223547
129	C	21.151424	0.000000	-2.061584
130	C	18.704296	0.000000	-6.382943
131	C	17.460379	0.000000	-8.541622
132	C	22.361027	0.000000	0.115917
133	H	16.174543	0.000000	-10.316726
134	H	23.220103	0.000000	2.132607
135	C	19.915191	0.000000	-2.789878
136	C	18.692279	0.000000	-4.948027
137	C	21.132803	0.000000	-0.622293
138	C	17.460484	0.000000	-7.108212
139	C	16.197850	0.000000	-9.226093
140	C	22.297229	0.000000	1.550930
141	C	18.669134	0.000000	-2.087689
142	C	17.448341	0.000000	-4.240129
143	C	15.002240	0.000000	-8.544950
144	C	16.218953	0.000000	-6.393936
145	C	19.880463	0.000000	0.074364
146	C	21.097578	0.000000	2.224906
147	H	14.054492	0.000000	-9.091820
148	H	21.078245	0.000000	3.318952
149	C	17.433380	0.000000	-2.808792
150	C	18.649477	0.000000	-0.653013
151	C	16.207826	0.000000	-4.963018
152	C	14.965349	0.000000	-7.110983
153	C	19.849476	0.000000	1.517821
154	C	17.403541	0.000000	0.046357
155	C	13.752732	0.000000	-6.402069
156	C	14.967561	0.000000	-4.255623
157	C	16.186740	0.000000	-2.101353
158	C	18.615956	0.000000	2.190161
159	H	12.808876	0.000000	-6.957222
160	H	18.603838	0.000000	3.285216
161	C	14.953042	0.000000	-2.824782
162	C	17.386022	0.000000	1.487480

163	C	16.164251	0.000000	-0.667269
164	C	13.717467	0.000000	-4.984906
165	C	16.139514	0.000000	2.164398
166	C	12.499909	0.000000	-4.262730
167	C	13.711169	0.000000	-2.114912
168	C	14.917220	0.000000	0.042368
169	H	16.171292	0.000000	3.255871
170	H	11.554587	0.000000	-4.815432
171	C	12.471332	0.000000	-2.852183
172	C	13.680608	0.000000	-0.685824
173	C	14.909085	0.000000	1.486806
174	C	11.245525	0.000000	-2.131368
175	C	12.422385	0.000000	0.013253
176	C	13.630014	0.000000	2.190335
177	H	14.493767	0.000000	4.200584
178	H	10.326861	0.000000	-2.722575
179	C	11.187131	0.000000	-0.735424
180	C	12.406347	0.000000	1.450785
181	C	13.568878	0.000000	3.621786
182	C	12.370794	0.000000	4.300968
183	C	11.140256	0.000000	2.151298
184	C	9.897568	0.000000	-0.015777
185	H	8.633981	0.000000	-1.768835
186	H	12.355641	0.000000	5.395058
187	C	9.908279	0.000000	1.438682
188	C	11.121103	0.000000	3.600408
189	C	8.674715	0.000000	-0.676785
190	C	9.898995	0.000000	4.291152
191	C	8.664020	0.000000	2.154178
192	C	7.419946	0.000000	0.008258
193	H	6.199411	0.000000	-1.775728
194	H	9.905360	0.000000	5.386222
195	C	8.657403	0.000000	3.609887
196	C	7.423660	0.000000	1.458071
197	C	6.197572	0.000000	-0.680447
198	C	7.425003	0.000000	4.301568
199	C	6.182779	0.000000	2.166935
200	C	4.949311	0.000000	0.002155
201	H	3.718029	0.000000	-1.778481
202	H	7.433214	0.000000	5.396644
203	C	6.183020	0.000000	3.624419
204	C	4.949418	0.000000	1.459002
205	C	3.717990	0.000000	-0.683314
206	C	4.945750	0.000000	4.311029
207	C	3.707511	0.000000	2.167194
208	C	2.475526	0.000000	0.001057
209	H	1.240784	0.000000	-1.779404
210	H	4.946740	0.000000	5.406132
211	C	3.707853	0.000000	3.626662
212	C	2.475068	0.000000	1.459421
213	C	1.240486	0.000000	-0.684260
214	C	2.468610	0.000000	4.311005
215	C	1.232151	0.000000	2.166734
216	C	0.000275	0.000000	-0.000080
217	H	-1.236149	0.000000	-1.780351
218	H	2.468349	0.000000	5.406133
219	C	1.231925	0.000000	3.625861
220	C	0.000050	0.000000	1.459047

221	C	-1.236409	0.000000	-0.685223
222	C	-0.008286	0.000000	4.310042
223	C	-1.242868	0.000000	2.166360
224	C	-2.475653	0.000000	-0.000880
225	H	-3.714539	0.000000	-1.780350
226	H	-0.008583	0.000000	5.405186
227	C	-1.243326	0.000000	3.624724
228	C	-2.475311	0.000000	1.458587
229	C	-3.713550	0.000000	-0.685248
230	C	-2.485790	0.000000	4.309095
231	C	-3.717217	0.000000	2.166779
232	C	-4.950819	0.000000	0.001362
233	H	-6.201013	0.000000	-1.770862
234	H	-2.485828	0.000000	5.404262
235	C	-3.717111	0.000000	3.623626
236	C	-4.950578	0.000000	1.458846
237	C	-6.192802	0.000000	-0.675787
238	C	-4.965371	0.000000	4.306228
239	C	-6.191459	0.000000	2.167710
240	C	-7.425202	0.000000	0.015895
241	H	-8.673158	0.000000	-1.760440
242	H	-4.967211	0.000000	5.401508
243	C	-6.187745	0.000000	3.617523
244	C	-7.431819	0.000000	1.471603
245	C	-8.666793	0.000000	-0.665371
246	C	-7.442514	0.000000	4.302565
247	C	-9.888901	0.000000	0.025374
248	C	-8.676077	0.000000	2.187099
249	H	-11.123437	0.000000	-1.769276
250	H	-7.401780	0.000000	5.394615
251	C	-8.665367	0.000000	3.641557
252	C	-9.908054	0.000000	1.474483
253	C	-11.138591	0.000000	-0.675187
254	C	-12.336675	0.000000	0.003995
255	C	-11.174145	0.000000	2.174995
256	C	-9.954930	0.000000	4.361205
257	H	-9.094660	0.000000	6.348354
258	H	-13.261564	0.000000	-0.574803
259	C	-12.397811	0.000000	1.435445
260	C	-11.190182	0.000000	3.612527
261	C	-10.013323	0.000000	5.757147
262	C	-13.676881	0.000000	2.138974
263	C	-12.448405	0.000000	4.311603
264	C	-11.239130	0.000000	6.477961
265	H	-10.322386	0.000000	8.441210
266	H	-14.939087	0.000000	0.369909
267	C	-13.685016	0.000000	3.583412
268	C	-12.478966	0.000000	5.740690
269	C	-11.267707	0.000000	7.888508
270	C	-14.907309	0.000000	1.461382
271	C	-12.485264	0.000000	8.610684
272	C	-14.932046	0.000000	4.293048
273	C	-16.153816	0.000000	2.138300
274	C	-13.720839	0.000000	6.450560
275	H	-17.371631	0.000000	0.340564
276	H	-11.576674	0.000000	10.582999
277	C	-17.383750	0.000000	1.435618
278	C	-14.954536	0.000000	5.727131

279	C	-13.735358	0.000000	7.881400
280	C	-12.520530	0.000000	10.027846
281	C	-16.171336	0.000000	3.579422
282	C	-18.617270	0.000000	2.107957
283	C	-13.733146	0.000000	10.736759
284	C	-14.975622	0.000000	8.588795
285	C	-17.417272	0.000000	4.278791
286	C	-16.201176	0.000000	6.434569
287	H	-19.846038	0.000000	0.306826
288	H	-12.822289	0.000000	12.717595
289	C	-19.865370	0.000000	1.400873
290	C	-18.648257	0.000000	3.551414
291	C	-14.986749	0.000000	10.019712
292	C	-13.770037	0.000000	12.170726
293	C	-16.216137	0.000000	7.865906
294	C	-17.436929	0.000000	5.713467
295	C	-21.065022	0.000000	2.074847
296	C	-14.965646	0.000000	12.851869
297	C	-16.228280	0.000000	10.733987
298	C	-19.900597	0.000000	4.248071
299	C	-17.460074	0.000000	8.573804
300	C	-18.682986	0.000000	6.415655
301	C	-21.987895	0.000000	1.493171
302	C	-14.942340	0.000000	13.942501
303	C	-21.128820	0.000000	3.509860
304	C	-16.228175	0.000000	12.167398
305	C	-17.472092	0.000000	10.008719
306	C	-19.919218	0.000000	5.687361
307	C	-18.697347	0.000000	7.849324
308	C	-22.381745	0.000000	4.221264
309	C	-17.483051	0.000000	12.875219
310	C	-18.715582	0.000000	10.713301
311	C	-21.163173	0.000000	6.390905
312	C	-19.944891	0.000000	8.555464
313	H	-23.656899	0.000000	2.444953
314	H	-16.616320	0.000000	14.882689
315	C	-18.723311	0.000000	12.149912
316	C	-22.398456	0.000000	5.658039
317	C	-19.956722	0.000000	9.989183
318	C	-21.180532	0.000000	7.827772
319	C	-23.632856	0.000000	3.535364
320	C	-17.538817	0.000000	14.300851
321	C	-24.830536	0.000000	4.216261
322	C	-18.739011	0.000000	14.977320
323	C	-19.976013	0.000000	12.857461
324	C	-23.649869	0.000000	6.368005
325	C	-21.204469	0.000000	10.694976
326	C	-22.427692	0.000000	8.534204
327	H	-25.753845	0.000000	3.635975
328	H	-18.716660	0.000000	16.067611
329	C	-24.883109	0.000000	5.641710
330	C	-19.988045	0.000000	14.288691
331	C	-22.441533	0.000000	9.968713
332	C	-21.218736	0.000000	12.130209
333	C	-23.665684	0.000000	7.807765
334	C	-22.464406	0.000000	12.834441
335	C	-24.910580	0.000000	8.513409
336	C	-23.691202	0.000000	10.676083

337	C	-26.138308	0.000000	6.353381
338	C	-21.244238	0.000000	14.998706
339	H	-20.373410	0.000000	17.003896
340	H	-27.409581	0.000000	4.574858
341	C	-26.150778	0.000000	7.785483
342	C	-22.478521	0.000000	14.272465
343	C	-23.704695	0.000000	12.110210
344	C	-24.927842	0.000000	9.949610
345	C	-21.296140	0.000000	16.422821
346	C	-27.386175	0.000000	5.665061
347	C	-22.495355	0.000000	17.101591
348	C	-28.585255	0.000000	6.344010
349	C	-26.176599	0.000000	10.656505
350	C	-24.953494	0.000000	12.817148
351	C	-23.730596	0.000000	14.981340
352	C	-27.402960	0.000000	8.494263
353	H	-22.472099	0.000000	18.191814
354	H	-29.508088	0.000000	5.763069
355	C	-26.190000	0.000000	12.090637
356	C	-23.742998	0.000000	16.413258
357	C	-28.637130	0.000000	7.768046
358	C	-24.970788	0.000000	14.253347
359	C	-27.416960	0.000000	9.932256
360	C	-26.215612	0.000000	14.959056
361	C	-28.662670	0.000000	10.636445
362	C	-27.439799	0.000000	12.798137
363	C	-24.998587	0.000000	17.125290
364	C	-29.893760	0.000000	8.478183
365	H	-24.128077	0.000000	19.130926
366	H	-31.165590	0.000000	6.699703
367	C	-27.453474	0.000000	14.232433
368	C	-28.676655	0.000000	12.071769
369	C	-26.231420	0.000000	16.398886
370	C	-29.905351	0.000000	9.909087
371	C	-25.051216	0.000000	18.550356
372	C	-31.142799	0.000000	7.789993
373	C	-26.249570	0.000000	19.231109
374	C	-32.343152	0.000000	8.467184
375	C	-28.700870	0.000000	14.939050
376	C	-29.924427	0.000000	12.777677
377	C	-27.483184	0.000000	17.109265
378	C	-31.158634	0.000000	10.616827
379	H	-26.225922	0.000000	20.321590
380	H	-33.266001	0.000000	7.885766
381	C	-29.935808	0.000000	14.211152
382	C	-28.718304	0.000000	16.375998
383	C	-31.165499	0.000000	12.053186
384	C	-27.499644	0.000000	18.545159
385	C	-32.398309	0.000000	9.892049
386	C	-31.184116	0.000000	14.917827
387	C	-29.962193	0.000000	17.079733
388	C	-32.409020	0.000000	12.757598
389	C	-28.754686	0.000000	19.257566
390	C	-33.654985	0.000000	10.601538
391	H	-27.897588	0.000000	21.274197
392	H	-34.943119	0.000000	8.828891
393	C	-31.198164	0.000000	16.350874
394	C	-32.420097	0.000000	14.192436

395	C	-29.980539	0.000000	18.518886
396	C	-33.652441	0.000000	12.032763
397	C	-28.819653	0.000000	20.691153
398	C	-34.917658	0.000000	9.919534
399	C	-32.445382	0.000000	17.053478
400	C	-33.664338	0.000000	14.900276
401	C	-31.234949	0.000000	19.216969
402	C	-34.896522	0.000000	12.749104
403	C	-30.021698	0.000000	21.364487
404	C	-36.113555	0.000000	10.603717
405	H	-30.041033	0.000000	22.458591
406	H	-37.061631	0.000000	10.057285
407	C	-33.677149	0.000000	16.329171
408	C	-32.464202	0.000000	18.489705
409	C	-34.905503	0.000000	14.177348
410	C	-31.266587	0.000000	20.657174
411	C	-36.147699	0.000000	12.035117
412	C	-34.927883	0.000000	17.037226
413	C	-33.710527	0.000000	19.191965
414	C	-36.149004	0.000000	14.884613
415	C	-32.507268	0.000000	21.332003
416	C	-37.364760	0.000000	12.751645
417	H	-32.515597	0.000000	22.427208
418	H	-38.308114	0.000000	12.195207
419	C	-34.942515	0.000000	18.466160
420	C	-36.160638	0.000000	16.314457
421	C	-33.727311	0.000000	20.634346
422	C	-37.394393	0.000000	14.156776
423	C	-36.199562	0.000000	19.172120
424	C	-37.412817	0.000000	17.029002
425	C	-34.999453	0.000000	21.313164
426	C	-38.631066	0.000000	14.898230
427	H	-35.001516	0.000000	22.407516
428	H	-39.570464	0.000000	14.336857
429	C	-37.402593	0.000000	18.438204
430	C	-36.182535	0.000000	20.613337
431	C	-38.639806	0.000000	16.272777
432	H	-38.355796	0.000000	18.977824
433	H	-37.140482	0.000000	21.142509
434	H	-39.586488	0.000000	16.821848

14. References

(1) Kwok, Y. H.; Xie, H.; Yam, C. Y.; Zheng, X.; Chen, G. H. Time-Dependent Density Functional Theory Quantum Transport Simulation in Non-Orthogonal Basis. *J. Chem. Phys.* **2013**, *139* (22), 224111.

(2) Zelovich, T.; Hansen, T.; Liu, Z.-F.; Neaton, J. B.; Kronik, L.; Hod, O. Parameter-Free Driven Liouville-von Neumann Approach for Time-Dependent Electronic Transport Simulations in Open Quantum Systems. *J. Chem. Phys.* **2017**, *146* (9), 092331.

(3) Elenewski, J. E.; Gruss, D.; Zwolak, M. Communication: Master Equations for Electron Transport: The Limits of the Markovian Limit. *J. Chem. Phys.* **2017**, *147* (15), 151101.

(4) Gruss, D.; Velizhanin, K. A.; Zwolak, M. Landauer's Formula with Finite-Time Relaxation: Kramers' Crossover in Electronic Transport. *Sci. Rep.* **2016**, *6* (1), 24514.

(5) Elenewski, J. E.; Wójtowicz, G.; Rams, M. M.; Zwolak, M. Performance of Reservoir Discretizations in Quantum Transport Simulations. *J. Chem. Phys.* **2021**, *155* (12), 124117.

(6) Oz, A; Nitzan, A; Hod, O; Peralta, JE. Electron Dynamics in Open Quantum Systems: The Driven Liouville-von Neumann Methodology within Time-Dependent Density Functional Theory. *J. Chem. Theory Comput.* **2023**, 19, 7496−7504.

(7) Herrmann, C.; Solomon, G. C.; Subotnik, J. E.; Mujica, V.; Ratner, M. A. Ghost transmission: How large basis sets can make electron transport calculations worse. J. Chem. Phys. 2010, 132.

(8) Reuter, M. G.; Harrison, R. J. Rethinking first-principles electron transport theories with projection operators: The problems caused by partitioning the basis set. J. Chem. Phys. 2013, 139.

(9) Son, Y.-W.; Cohen, M. L.; Louie, S. G. Half-metallic graphene nanoribbons. nature **2006**, 444, 347–349.

(10) Hod, O.; Barone, V.; Peralta, J. E.; Scuseria, G. E. Enhanced half-metallicity in edge-oxidized zigzag graphene nanoribbons. Nano letters **2007**, 7, 2295–2299.
